# Synthesis and bio-molecular study of (+)-N-Acetyl-α-amino acid dehydroabietylamine derivative for the selective therapy of hepatocellular carcinoma

**DOI:** 10.1186/s12885-016-2942-5

**Published:** 2016-11-14

**Authors:** Muhammad Ayaz Mustufa, Cigdem Ozen, Imran Ali Hashmi, Afshan Aslam, Jameel Ahmed Baig, Gokhan Yildiz, Shoaib Muhammad, Imam Bakhsh Solangi, Naim ul Hasan Naqvi, Mehmet Ozturk, Firdous Imran Ali

**Affiliations:** 1 5th Floor, PHRC Specialized Research Centre on Child Health, National Institute of Child Health, Karachi, 75500 Pakistan; 2 Baqai Institute of Pharmaceutical Sciences (BIPS), Baqai Medical University, Karachi, 74600 Pakistan; 3 Department of Chemistry, University of Karachi, Karachi, 75270 Pakistan; 4 National Center of Excellence in Analytical Chemistry, University of Sindh, Jamshoro, 76080 Pakistan; 5 Dr. M. A. Kazi Institute of Chemistry, University of Sindh, Jamshoro, 76080 Pakistan; 6 Department of Molecular, Biology and Genetics, BilGen Genetics and Biotechnology Center, Bilkent University, Ankara, 06800 Turkey; 7 Izmir International Biomedicine and Genome Institute, iBG-izmir, Dokuz Eylül University, 35340 Balcova, Izmir Turkey; 8 Department of Medical Biology, Erzincan University Faculty of Medicine, Erzincan, 24100 Turkey

**Keywords:** Dehydroabietylamine derivatives, Hepatocellular carcinoma, Cytotoxicity, Connectivity map, Apoptosis

## Abstract

**Background:**

The purpose of present work is to synthesize novel (+)-Dehydroabietylamine derivatives (DAAD) using *N*-acetyl-α-amino acid conjugates and determine its cytotoxic effects on hepatocellular carcinoma cells.

**Methods:**

An analytical study was conducted to explore cytotoxic activity of DAAD on hepatocellular carcinoma cell lines. The cytotoxicity effect was recorded using sulforhodamine B technique. Cell cycle analysis was performed using Propidium Iodide (PI) staining. Based on cell morphology, anti growth activity and microarray findings of DAAD2 treatment, Comet assay, Annexin V/PI staining, Immunoperoxidase assay and western blots were performed accoringly.

**Results:**

Hep3B cells were found to be the most sensitive with IC_50_ of 2.00 ± 0.4 μM against (+)-N-(N-Acetyl-L-Cysteine)-dehydroabietylamine as DAAD2. In compliance to time dependent morphological changes of low cellular confluence, detachment and rounding of DAAD2 treated cells; noticeable changes in G_2_/M phase were recorded may be leading to cell cycle cessation. Up-regulation (5folds) of TUBA1A gene in Hep3B cells was determined in microarray experiments. Apoptotic mode of cell death was evaluated using standardized staining procedures including comet assay and annexin V/PI staining, Immuno-peroxidase assay. Using western blotting technique, caspase dependant apoptotic mode of cell death was recorded against Hep3B cell line.

**Conclusion:**

It is concluded that a novel DAAD2 with IC_50_ values less than 8 μM can induce massive cell attenuation following caspase dependent apoptotic cell death in Hep3B cells. Moreover, the corelation study indicated that DAAD2 may have vital influence on cell prolifration properties.

**Electronic supplementary material:**

The online version of this article (doi:10.1186/s12885-016-2942-5) contains supplementary material, which is available to authorized users.

## Background

Hepatocellular carcinoma (HCC) is the most common primary liver cancer. It is the fifth most frequent neoplasm and the third largest mortality causing cancer [1, 2]. The average life of HCC is 6 months after diagnosis including surgical treatment options, suggesting poor prognosis of the disease [1–3]. However, the highest incidence of HCC reported in Asia and Africa [4]. Thus, it is an alarming situation that annually around about 600,000 individuals lost their lives due to HCC and almost the same number of new cases registered every year [5, 6]. The HCC in humans can be caused by multi-factors such as: high intake of alcohol and Aflatoxin, hereditary disorders and chronic infections with HCV and HBV [7, 8]. These factors have direct impact on patient characteristics as well as the tumor progression [9]. The traditionally available therapies (chemotherapy or surgery) for the treatment of HCC are still not very successful in terms of quality and survival. However, Sorafenib is the first oral multikinase inhibitor and only the approved drug by Food and Drug Administration (FDA) for the treatment of HCC [10, 11]. These disappointing facts and figures suggested a dire need of effective drugs for the treatments of HCC.

Considering that structural manipulation is still remarkable and effective technique for the enhancement of drug efficacy and associated with the finding of novel drugs with potentially broad bioactive spectra. The (+)-Dehydroabietylamines (DAA) and its derivatives reported for biological activities including cytotoxic effects [12–15], endo-peptidase inhibitory activity, antibacterial [16, 17], antitumoral [18], and anti-carcinogenesis activity [19].

*N*-acetyl amino acids may have a fundamental role in several physiological and biochemical functions of the human body [20]. Therefore, currently seven N-acetyl amino acids were used to synthesize seven derivatives of DAA through the association of peptide linkage between primary amine of DAA and carboxylic acid of *N*-acetyl amino acids. Meanwhile, the proposed drugs may have greater than 25% carboxamide functionality [20]. However, the carbodiimide mediated peptide coupling method remains to be the most frequently used technique, as a major advantage, the carbodiimide did not require prior activation of carboxylic acid [20]. Thus, the purpose of this study was first to synthesize seven derivatives of DAA using seven *N*-acetyl amino acids and their characterization by different analytical techniques. Moreover, the proposed novel (+)-Dehydroabietylamine analogues were used to examine the in vitro cytotoxicity of one breast cancer and ten HCC cell lines in detail.

## Methods

### Reagents and apparatus

All reagents and chemicals used during experimental work were analytical grade. The N- *N*-acetyl amino acids, (+)-Dehydroabietylamine, dehydroabietylamine, dichloromethane, dicyclohexyl carbodiimide, 4-dimethyl amino pyridine, ethanol, methanol and ethyl acetate purchased from Sigma–Aldrich (St. Louis, MO, USA). DMSO was obtained from Applichem Biochemica (Darmstadt, Germany). Analytical thin-layer chromatography was performed on precoated silica gel plates (Merck PF254, Darmstadt, Germany) and detection of spots were made by UV light and/or iodine vapors. Silica gel 60 (Merck, particle, size 0.040–0.063 mm, 230–240 mesh) was used for preparative column chromatography.

IR spectra were recorded on a Thermo-Nicolet 5700 Fourier transform infrared (FTIR) spectrometer (Fitchburg, WI) with KBr pellets. NMR spectra were recorded at room temperature on Bruker AM instrument operating at 400 and 500 MHz (^1^H). Residual solvent signals are internally referenced. Chemical shifts *δ* is referred in terms of ppm, coupling constants *J* are given in Hz. Following abbreviations classify the multiplicity: s = singlet, d = doublet, t = triplet, q = quartet, m = multiplet or unresolved, br = broad signal. Infrared spectra were recorded on a Schimadzu system and reported in cm^−1^. Samples were prepared in thin film technique. Mass spectra were done using the facilities in laboratories of HEJ Research institute of Chemistry, University of Karachi.

### Synthesis

#### General procedure for the preparation of Amides from amino acids (DAAD 1–7)

To a solution of dehydroabietylamine (1 mol) in dichloromethane, dicyclohexyl carbodiimide (DCC, 1 mol), 4-dimethyl amino pyridine (DMAP, 0.5 mol) and N-acetyl amino acids (1 mol) were added, the resulting mixture was stirred at room temperature for 2 h. After completion of reaction 50 ml of ethyl acetate was added and filter. Filtrate washed with water (20 ml×2), and dried over anhydrous Na_2_SO_4_ and the solvent removed by evaporation. The formations of DAAD (1–7) were monitored by TLC; the compound was extracted from reaction mixture and characterized by applying different spectroscopic techniques. Formation of amide was completed in 2 to 2.5 h. N-acetyl-α-amino acid conjugates of (+)-Dehydroabietylamine (DAA) were prepared using Glycine, *L-*Cysteine, *L-*Methionine, *L-*Tyrosine, *L-*Aspartic acid, *L-*Phenylalanine and *L-*Alanine to furnish amides 1–7 respectively. All amides were obtained in good yields. The structure confirmation of compounds 1–7 was carried out (Additional file 1: Suppl. data Chemistry and Additional file 2: Characterization and Synthesis)​ using spectroscopic techniques including ESI-MS, FTIR, ^1^H-NMR, 1D & 2D, ^13^C-NMR, COSY, HMBC, HSQC and NOESY spectroscopy.

### Cell lines care and cytotoxicity screening protocol

Ten HCC cell lines including Huh7, Hep3B, Hep3B-TR, HepG2, Hep40, SNU449, Mahlavu, PLC/PRF/5, SNU387, SNU475 and one breast cancer line MCF7 cryogenically stored in a liquid nitrogen Tank. Sulforhodamine B (SRB) method has been implemented for in vitro primary screening [21, 22]. Initially, a HCC cell line Huh7 and one breast cancer line MCF7 were used for primary screening of trial drugs (seven derivatives of synthesized DAA). For both Huh7 and MCF7 cell lines, 2000 cells/well were cultured in 96 well plate in incubator at 37 °C with 5% CO_2_ in complete medium (DMEM, 10% FBS, 1% NEA, 1% *L-*Glutamine and 1% P/S) and incubated for 24 h. All stock solutions were prepared in 100% DMSO with a concentration of 20 mM. Further dilutions were made with the help of respective media for each cell line. After 24 h, all trial drugs were introduced in two different (50 μM and 10 μM) concentrations in triplicate for each sample and plates were further incubated for next 72 h. After 72 h, media discarded and cells were washed once by using 1_X_PBS.

For fixation, 50 μL of ice cold 10% TCA was added into each well and kept in dark at 4 °C for 1 h. After fixation, the TCA was removed by tapping and plates were washed 4–5 times with ~200 μL dH_2_O. Plates were left over night for drying under hood. Finally, 50 μL of 0.4% SRB in 1% acetic acid solution was added to each well and left at room temperature for 10 min. Excessive SRB dye was removed and the plates washed 4–5 times with 1% acetic acid before air drying. Bound SRB dye was solubilized with 100 μL of 10 mM un-buffered chilled tris-base solution and plates were left on a plate shaker for at least 1–2 min. Absorbance was recorded using μ-Quant microplate reader with a wave length range of 405–515 nm. The test OD values were defined as the absorbance of each sample. Mean values were determined and standard deviation was found satisfactory ranging in between 0.001 to 0.25 in all respective samples from triplicates wells which were calculated automatically using MS Office Excel 2007 (v14.0) software for Windows.

### Measurement of cell morphology

Six-well plates were used for photographs and 3 × 10^5^ cells were maintained in each well, after 24 h drug was administered. Photographs of most sensitive (Hep3B) cell line were taken during three consecutive intervals i.e., 24, 48 and 72 h and compared with DMSO controls. All photographs were taken at 10X and 20X using a light microscope.

### Cell proliferation assay

Cells were plated in 10 cm^2^ Petri plates at 3–4 × 10^5^ per plate. After drug treatment, cells were harvested in different intervals by trypsinization and washed with PBS. Cells were fixed in ice-cold 70% ethanol, washed, and resuspended in 3 mL of 70% ethanol for storage at 4 °C; fixed cells treated with RNase A; and stained with propidium iodide (PI) for 45 min at room temperature. The stained cells were analyzed by flow cytometry using BD FACScalibur™.

### Comet assay [23]

Hep3B cell line was used and around 200,000 cells/well were seeded in six well plates. Adriamycin (1 μM), and Camptothecin (5 μM) were used as positive controls. DAAD2 was administered in two different concentrations (5uM and 10 μM). Drugs were given to cells after 24 h of seeding. Both treated and untreated samples were collected after 24,48 and 72 h from plates through scraper with whole media after centrifugation at 1000 rpm for 10 min at +4 °C.

Freshly prepared 100 mL 1% normal melting agarose poured into a jar and kept in a water bath at 50–55 °C. The slides were placed into jar vertically and kept for about 15–20 min at room temperature. 15 ml of 0.7% low melting agarose (LMA) was also prepared and kept it at 4 °C. Both controls and treated cells were taken from incubator and washed with 1X cold PBS once. 2 mL of 1XPBS for each well of 6 wells plate added and cells were transferred directly in to 15 mL falcon tubes. The cells were centrifuged at 1000 rpm for 6 min at 4 °C. Supernatant was discarded and pallet of the cells re-suspend with cold PBS (700 μL) without bubbling. 5–10 μL cell suspension was put on already prepared 1% agarose coated slide, and coated with 70–80 μL LMA with cover slip (24 × 50 mm) onto the cells. Cells were counted under the microscope to validate the desired cell number and stored for 12–15 min at 4 °C to allow LMA layer to soluble. For third layer, again 70–80 μL of LMA covered with cover slip was used for coating, and the slides were kept at 4 °C for about 12–15 min. Slides were pour in to tank for at least 1 h containing lysis buffer and importantly temperature was maintained at 4 °C. Slides were taken out and washed with neutralization buffer thrice. In the next step, freshly prepared electrophoresis buffer (0.555 g Na-EDTA + 10 g NaOH cold 1.5 L dH_2_O maintained at 4 °C) was poured into electrophoresis tank and the tank was covered with ice to maintain the temperature before use. The washed slides were placed without any gap into the tank as writing place of slides on positive side of the electrode. Power supply for electrolysis tank was maintained on 25 V and maximum up to 280 A. Incubation of the slides was done in dark with electrophoresis buffer for 20 min to denature DNA and then DNA was run at 25 V per 260–280 A per 20 min. At last, slides were taken out and washed with neutralization buffer thrice gently and kept slides into the buffer until DAPI staining. For DAPI staining, 45 μL, (5 μg/mL DAPI) with 24 × 50 mm coverslip onto the slides was applied and kept into the humidity chamber at (papers with water) 4 °C before taking the pictures on fluorescent microscope.

### Propidium iodide staining [24]

Hep3B cells were maintained in 5 cm^2^ Petri plates and cover slips were added at the time of splitting. After 24 h, DAAD2 (2 μM & 5 μM) was supplemented. While, media with less than 0.1% DMSO were also changed for negative controls. After 72 h of incubation, cover slips from both control and sample were taken out and as per given protocol cover slips were covered with annexin V/PI staining solution for 15 min. Photographs were taken at fluorescent microscope with detection range of 515–565 nm (green).

### Microarray gene-expression analyses [25, 26]

Affymetrix Human Genome U133 Plus 2.0 Gene-Chips were used for whole-genome gene expression profiling experiments. Isolated total RNAs of treated and non-treated DAAD2-sensitive HEP3B and DAAD2-resistant SNU449 cells processed according to manufacturer’s instructions. Quality control analyses of microarray data performed using the BRB Array Tools V 4.2.0 (http://linus.nci.nih.gov/BRB-ArrayTools.html). Triplicate samples from each sample type (12 samples in total) were used for the rest of the analyses. Normalizations of the raw data obtained from Gene-Chip Operating Software performed using the BRB Array Tools.

Lists of differentially expressed genes were determined using the Class Comparison Tool of the BRB Array Tools software. Genes differing more than 1.5 fold between control and DAAD-2 treated Hep3B cells, as well as control and DAAD-2 treated SNU449 cells were determined in separate lists.

Lists of differentially expressed genes were further analyzed via Connectivity Map (cmap) Tool [27] to further investigate molecular mechanisms responsible for resistance and sensitivity to DAAD-2 treatments. Gene expression signatures of drug-sensitive and drug-resistant HCC cells were used to determine similarities with signatures of previously characterized chemicals in the cmap database.

### Immuno-peroxidase assay [28]

Hep3B cells were plated for 24 h in 12 well/plates at 50,000 cells on cover-slips for per each well. After DAAD2 treatment (2 μM & 5 μM) of 48 h, medium was aspirated. Cells were fixed with acetone and methanol mix solution (1:1) at −20 °C for 10 min. After aspiration of fixation solution, three times washing with 1XPBS performed for each plate. Hydrogen peroxide was added and hold for 10 min at room temperature in dark to block endogenous per oxidase activity. Washing step was repeated thrice again. Blocking was done using 10% FBS and 0.3% Triton X-100 in 1X PBS solution for 1 h in dark at room temperature. Anti human Caspase-3 (cleaved) was diluted in blocking solution (1:1000) and 100 μl of primary antibody was added on top of the cover slip drop by drop. After overnight incubation at 4 °C, cover-slips were washed with 1X PBS-T solution. 80 mL of Dako Envision secondary antibody was added on the top of the coverslips drop by drop and incubated in dark for 1 h at room temperature. Washing with 1X PBS-T solution performed twice. DAB solution was prepared and used as per manufacture guidelines. Counterstaining with hematoxylin solution was done for 5 min. at room temperature. After vigorous washing, coverslips were stained with blueing solution (0.1% Sodium Bicarbonate in distilled water) for 1 min at room temperature and rinsed with water. Finally, 85% of glycerol was added on top of each coverslip and put upside down on glass slide.

### Western blotting [29]

To determine the protein level of differentially expressed genes of interest, cells were treated with 2 μM dose of DAAD2 for 72 h and the lysed with a Radioimmunoprecipitation Assay (RIPA) Buffer. Concentrations of protein lysates were measured by the conventional Bradford assay utilizing spectrophotometer at 595 nm. Sample protein concentrations were normalized in accordance with bovine serum albumin (BSA) standard curve. Around 30 μg of total proteins were subjected to gel electrophoresis using NuPAGE system with MES and/or MOPS buffers. Proteins were wet-transferred onto HyBond ECL nitrocellulose membranes. The membranes were blocked for 1 h at room temperature with 5% BSA in TBS-T. Membranes were incubated with the primary antibodies either at room temperature or at 4 °C for overnight. Following primary antibody incubations and extensive washing with TBS-T, secondary antibodies conjugated with horse-radish peroxidase (HRP) incubated 1 h at room temperature. After an additional wash of half an hour with TBSt-T, chemiluminescent reaction was recorded using ECL prime western blot detection kit (Thermoscientific), as per manufacturer’s guidelines. X-ray films were exposed to the emitted chemiluminescence.

### Statistical analysis

The 19th version of SPSS (SPSS, Chicago, IL, USA) and Microsoft Excel 2007 (Roselle, IL, USA) were used for the statistical and graphical evaluations. Data were collected and expressed as the mean ± standard deviation of three independent experiments. Statistical analysis was performed by correlation of determination (R^2^) and probability test.

## Results and discussion

### Growth attenuation experiments and IC_50_ determination

**Fig. 1:**
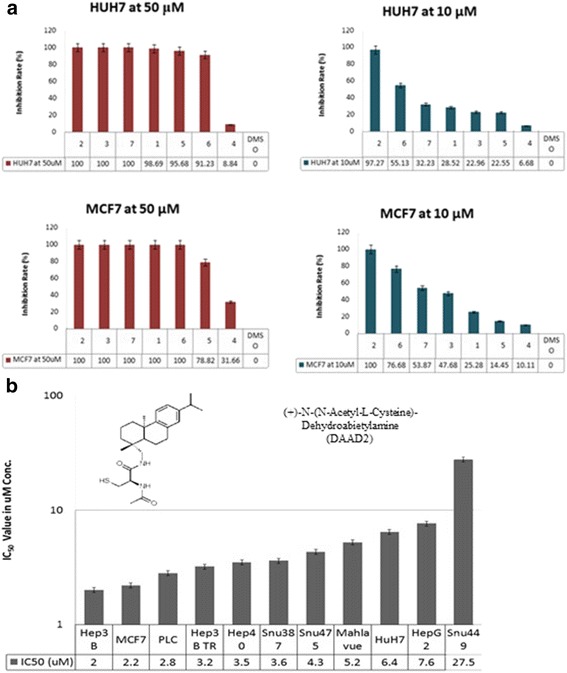
**a** Primary SRB (sulforhodamine B) screening results: Cytotoxic effects of seven novel compounds labeled as 1–7 (concentration of 50 μM and 10 μM), on Huh7 and MCF7 cell lines. **b** Molecular structure and cytotoxic activity with IC_50_ values of DAAD2 on 10 HCC and one breast cancer cell lines

In recent study, synthesized seven DAA derivatives (DAAD 1–7) were screened for cytotoxic effect on breast cancer and HCC cell lines by SRB technique as described in experimental part. Based on primary screening results (Fig. 1 and Additional file 3: Figure S1), DAAD 2 with a functional site of *N*-acetyl cysteine group at C-18 (2R) was found to be the most potent with 100% growth attenuation at 50 μM and 10 μM concentration on both Huh7 and MCF7 cells. Thus, it was selected for further investigation of cytotoxic effect and its mechanism. In continuation, IC_50_ values of DAAD2 were determined for all cell lines. All cells were treated with DAAD2 at different concentrations in triplicates ranging from 10 μM to 0.313 μM; for controls up to 0.1% DMSO final concentration was maintained with respective media for each cell line; camptothecin (CPT) as a positive control was used in triplicate at 5 μM concentration for all experiment. The IC_50_ values against all cell lines calculated using OD values against each drug concentration. Camptothecin showed around 80% growth attenuation at 5 μM concentration on Hep3B cells. While, inhibition rate of camptothecin found more than 90% on rest of cell cells lines.

As presented in Table 1, except SNU449 cell line with IC_50_ value more than 10 μM; all other cell lines showed 95–100% growth attenuation at 10 μM drug concentration of DAAD2, with IC_50_ range from 2 to 8 μM; indicating that this drug might have comparatively extensive therapeutic index than other trial drugs of the study. According to Table 1 and graph curve (Fig. 1b), Hep3B cells found to be the most sensitive cell line with the IC_50_ value of 2.0 ± 0.4 μM and reported around 90% inhibition at 5 μM drug treatment after 72 h.

**Table 1:** IC_50_ Values of 11 cell lines using Trial Drug (DAAD2)

Cell Lines	IC_50_ Value of DAAD2 (μM conc.)	R^2^
Hep3B	2.0 ± 0.4	0.9
MCF7	2.2 ± 0.8	0.8
PLC	2.8 ± 0.6	0.9
Hep3B-TR	3.2 ± 0.8	0.8
Hep40	3.5 ± 1	0.8
SNU387	3.6 ± 0.6	0.8
SNU475	4.4 ± 0.6	0.7
Mahlavu	5.2 ± 0.9	0.6
Huh7	6.4 ± 2	0.6
HepG2	7.6 ± 0.8	0.5
SNU449	27.5 ± 5	0.7

### Morphology using light microscopy

**Fig. 2:**
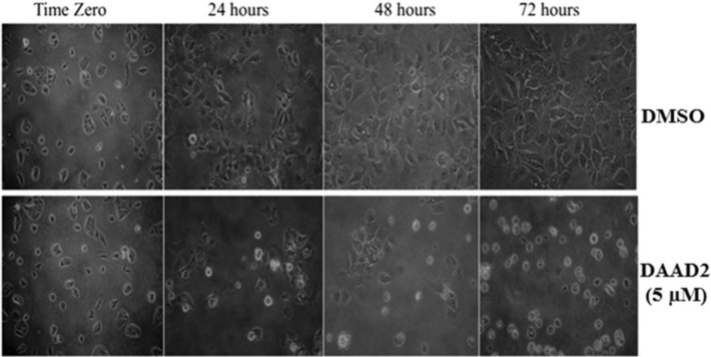
Cell Morphology of DAAD2 treated and untreated cells: Noticable morphological changes resulting from low cell confluency and loss of cellular attachment with rounding of cells is depicted. The time dependent pattern of these morphological changes from 24 to 72 h can be seen

Hep3B cells were treated with DAAD2 on 10 cm^2^ Petri plates; using a light microscope the differences in cell morphology was observed in DAAD2 treated and untreated cells. Photographs were taken at 20X after 24, 48 and 72 h consecutively. Noticeable morphological changes resulting from low cell confluence, enlarged nuclei, cell rounding and loss of cellular attachment were observed at 24 h. The extent of these morphological changes gradually increased from 24 to 72 h of drug treatment. These morphological changes suggested further biochemical and molecular investigation to rationalize these morphological outcomes (Fig. 2).

### G_2_/M phase arrest after 48 and 72 hours

**Fig. 3:**
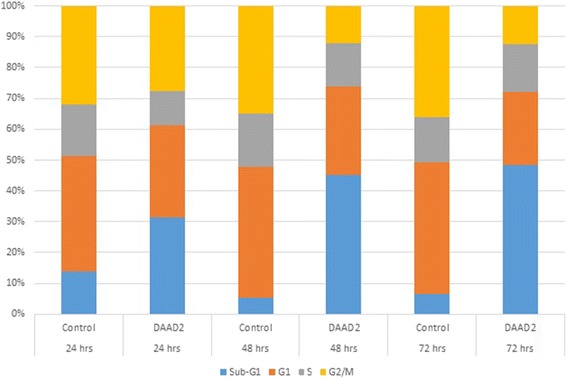
Cell cycle results after 24, 48 and 72 h of DAAD2 (5 μM concentration) treated Hep3B cells and DMSO controls (up to 0.1%) as untreated cells. Comparative to un-treated Hep3B cells, noticeable changes in sub-G1/G0 and G2/M phase of DAAD2 treated Hep3B cells are shown

To further validate inhibitory influence of our most potent trial drug DAAD2; cell cycle proliferation technique was adopted for quantitative analysis of cell progression in comparison with DMSO control. As described in methods part, Hep3B cells were studied accordingly. The percentages of cells in each phase of the cell cycle (Sub G1/G0, G1, S and G2/M) were determined 24, 48 and 72 h after treatment. As shown in Fig. 3, after 24 h of DAAD2 treatment although there was no significant change reported in G2/M phase. But after 48 and 72 h of DAAD2 treatment, significant (p < 0.001) decrease in the proportion of cells in G2/M phase; and nine-fold increase (p < 0.001) was recorded in sub-G1 phase after 48 and 72 h. In contrast, there was no noticeable change recorded (p > 0.05) in S phase after 48 and 72 h, whereas the percentage of G1 phase cells also decreased (p < 0.05) in DAAD2 treated cells (Fig. 3).

### Alkaline comet assay

**Fig. 4:**
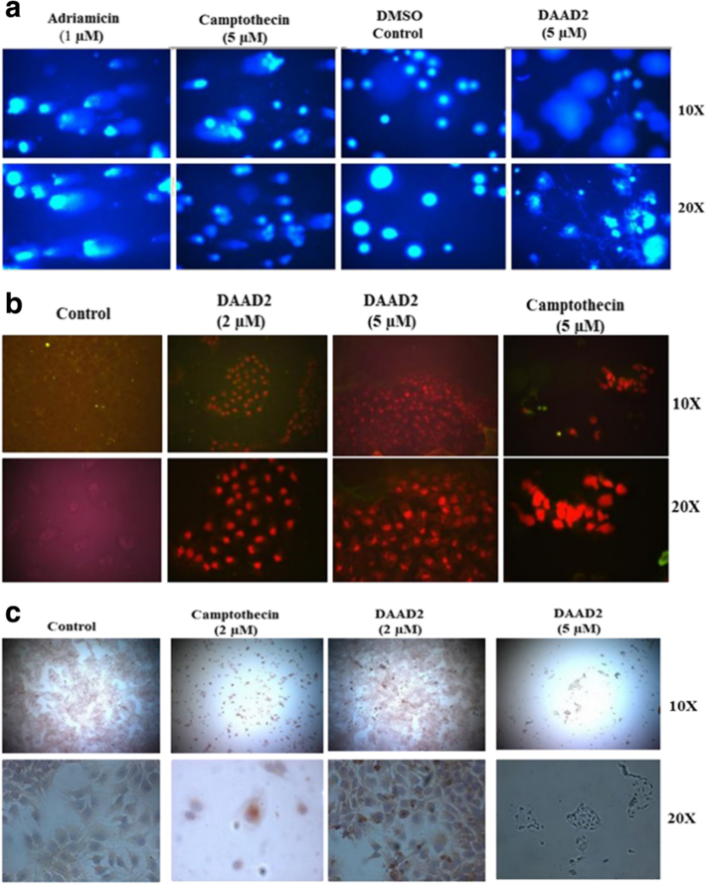
**a** Comet assay for DNA breaks: DAAD2 (5 μM), adriamycine (1 μM), camptothecin (5 μM) and DMSO controls (up to 0.1%). Photographs were taken after 72 h; obvious alkaline comets are shown in Hep3B cells treated with adriamycine and camptothecin. **b** Annexin V/PI Staining: DAAD2 (2 & 5 μM), camptothecin (5 μM) and DMSO controls (up to 0.1%). Photographs were taken after 72 h. DAAD2 treated Hep3B cells showed the apoptotic mode of cell death. **c** Immunoperoxidase Assay: Compound DAAD2 (2 & 5 μM), camptothecin (5 μM) and DMSO controls (upto 0.1%). Photographs were taken after 48 h. DAAD2 (2 μM) treated cells showed weak activation of caspase-3 cleaved

We performed alkaline comet assay to see the tail movement of single stranded DNA breaks. Hep3B cell line was used and around 200,000 cells/well were seeded in six well plates. Adriamycin (1 μM), and Camptothecin (5 μM) were used as positive controls. Trial drug DAAD2 was administered in two different concentrations (5 μM and 10 μM). Photographs were taken after 24, 48 and 72 h. No obvious alkaline comets were seen by applying trial drug DAAD2 on Hep3B cells (Fig. 4a). At 10 μM of DAAD2 drug administration, 24 and 48 h photographs were taken. But DAPI stained cells were not seen after 72 h and therefore photographs were not taken. The rapid growth attenuation was observed at 10 μM DAAD2 drug administration’s on Hep3B cells. Both Adriamycin and Camptothecin showed obvious alkaline comets. The DAAD2 treated cells (after 48 and 72 h) at 5 μM showed neutral comets may be of double stranded DNA breaks. (Fig. 4a)

### Class comparison and connectivity Map approach

In order to be able to determine molecular effects of DAAD2 treatment to liver cells, we determined whole-genome transcriptome profiles of DAAD2 treated and untreated (control) Hep3B and SNU 449 cells. Class comparison analyses using microarray data of DAAD2-sensitive Hep3B cell line (Additional file 4: Table S1), and DAAD2-resistant SNU449 cell line (Additional file 5: Table S2) provided the lists of genes differentially expressed as a result of DAAD2 treatments. Using differentially expressed gene results of drug-sensitive Hep3B and drug-resistant SNU 449 cells the connectivity map (cmap) analyses employed to determine the lists of previously characterized drugs that show similar and different gene expression profiles as a result of DAAD2 treatment. The Additional file 6: Figure S3 showed top 20 drugs with similar gene expression changes. Cmap analyses suggests that DAAD2 treatment triggers similar gene expression alterations as vinburnine does [30], since vinburnine and DAAD-2 treatments caused similar gene expression changes in drug-sensitive Hep3B cells, but highly different gene expression alterations in drug-resistant SNU449 cells (Additional file 7: Figures S2 and Additional file 6: Figures S3). In addition, we recorded more than 5-fold up regulation of TUBA1A gene in Hep3B treated cells, which has significant role in spindle formation at mitotic stage of cell cycle (Additional file 4: Table S1). To validate that DAAD2 may have some significant role to diverge the G2/M phase of cell proliferation, conformational studies were required.

### Apoptosis study of Hep3B cells

In recent era, Annexin V/Propidium iodide (PI) protocol is considered to be one of the commonly used approach for identification of apoptotic cells [24]. Annexin V in combination with PI is widely used to determine cell viability, apoptosis, or necrosis through deviations in permeability and integrity of plasma membrane. Due to stable, inexpensive, ability to not enter into the nucleus of live cells and believe to be a good indicator for cell viability than other available nuclear stains, PI is more commonly used. As shown in Fig. 4b, DAAD2 treated Hep3B cells showed high apoptotic mode of cell death at two different concentrations (2 μM & 5 μM).

### Caspase dependent apoptotic mode of cell death

Cysteine aspartate proteases commonly known as caspases have very vital role in initiation and execution of apoptotic process inside the cell. Therefore, based on above mentioned cell progression results [31], we performed Immuno-peroxidase assay on DAAD2 treated cells to determine the role of caspase-3 cleaved. It is reported that after initial activation of acaspase-9, ROS production is inhibited by caspase-3 [28] essentially needed for programmed cell death. As presented in Fig. 4c, low activation of caspase-3 cleaved was observed at 2 μM, while at 5 μM after 48 h of DAAD2 treatment all cells were unstained and mortal; unable us to identify the role and conclude the activation of caspase-3.

**Fig. 5:**
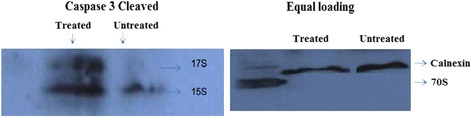
Western Blotting: DAAD2 (2 μM) and DMSO controls (up to 0.1%). Findings after 72 h of DAAD2 treatments. DAAD2 treated cells showed activation of caspase-3 (cleaved). In addition, calnexin was used as an equal loading control

Because of weak positive findings of caspase-3 cleaved through Immuno-peroxidase assay; we further evaluated role of caspase-3 cleaved using western blotting technique. Results were also validated through equal loading of calnexin [29]. (Figure 5) Our findings significantly highlight the activation of caspase-3 cleaved (17kda) in DAAD2 treated cells, while no sign of activation of same enzyme were observed in controls (untreated).

## Conclusions

In the first part of this study seven new (+)-Dehydroabietylamine derivatives with *N*-acetyl-α-amino acids at C-18 on ring A were synthesized, purified and characterized. In the second part, cytotoxic effects were recorded in a dose dependent manner for each new derivative. DAAD2 ((+)-Dehydroabietylamine with *N*-acetyl cysteine in ring A at C-18) was found to be the most potent among all trial drugs of the study; subjected for cytotoxic effects on ten HCCs and one breast cancer cell line. Hep3B cells were the most sensitive with IC_50_ of 2.00 ± 0.4 μM against DAAD2. Similarly, obvious morphological deviations and significant time dependent changes in sub-G1 (8–9 fold increase) and G2/M phase (2 fold decrease) in Hep3B cells after 48 and 72 h of DAAD2 treatments were recorded.

Whole-genome gene expression microarray data of DAAD2-sensitive and DAAD2-resistant HCC cells revealed that DAAD2 treatment cause similar gene expression alterations in HCC cells as vinburnine does to PC3 cells [Additional file 4: Table S1 and Additional file 5: Table S2]. Unfortunately, there are no comprehensive data on molecular functions of vinburnine to help us understand the molecular effects of DAAD2 on HCC cells. The DAAD2 showed caspase dependent apoptotic mode of cell death. The present outcomes exhibit the structural significance of our trial novel drug, its vital influence and correlation on cytotoxic and anti cancer properties.

## Additional files

Additional file 1: Suppl. data Chemistry. Original spectra. Original spectra of ESI-MS, FTIR, 1H-NMR, 1D & 2D, 13C-NMR, COSY, HMBC, HSQC and NOESY spectroscopy of compounds 1–7. (PDF 5506 kb)
Additional file 2: Characterization and Synthesis. Spectroscopic data. Characterization of compounds through spectral data. (PDF 366 kb)
Additional file 3:
**Figure S1.** Inhibition Curves against DAAD-2. Inhibition curves (ranging from 0.313 to 10 μM concentration) of Hep3B, Hep3B-TR, PLC and MCF7 cell lines against DAAD-2. (PDF 144 kb)
Additional file 4:
**Table S1.** Microarray data of DAAD2-sensitive Hep3B cell line. Class comparison analyses using microarray data of DAAD2-sensitive Hep3B cell line provided the lists of genes differentially expressed as a result of DAAD2 treatments. (XLS 229 kb)
Additional file 5:
**Table S2.** Microarray data of DAAD2-resistance SNU449 cell line. Class comparison analyses using microarray data of DAAD2-resistant SNU449 cell line provided the lists of genes differentially expressed as a result of DAAD2 treatments. (XLS 446 kb)
Additional file 6:
**Figure S3.** Connectivity map of DAAD2 treated SNU449 cells. DAAD-2 treatments caused highly different gene expression alterations in drug-resistant SNU449 cells. (PDF 242 kb)
Additional file 7:
**Figure S2.** Connectivity map of DAAD2 treated Hep3B cells. DAAD-2 treatments caused gene expression changes in drug-sensitive Hep3B cells similar to other drug molecules. (PDF 163 kb)

## Abbreviations

CPT: Camptothecin
DAA: (+)-Dehydroabietylamine
DCC: Dicyclohexylcarbodiimide
DMAP: N,N-Dimethyl amino pyridine
DMEM: Dulbecco’s modified eagle medium
FBS: Fetal Bovine serum
FITC: Fluorescein isothiocyanate
HPLC: High performance liquid chromatography
IC_50_: The half maximal growth inhibitory concentration
NMR: Nuclear magnetic resonance
OD: Optical density
P/S: Penicillin/Streptomycin
PBS: Phosphate buffer saline
RPMI: Roswell Park Memorial Institute medium
SD: Standard deviation
SRB: Sulforhodamine B
TBS: Tris-buffered saline
TCA: Tri-chloroacetic acid
UV: Ultra violet
