# Supplementary material for: Synthesis and bio-molecular study of (+)-N-Acetyl-α-amino acid dehydroabietylamine derivative for the selective therapy of hepatocellular carcinoma
Source: BMC Cancer. 2016 Nov 14;16:883. doi: 10.1186/s12885-016-2942-5 (PMC5109647; doi:10.1186/s12885-016-2942-5)

Table of Contents

^{1}H-NMR, ^{13}C-NMR BB, ^{13}C-NMR DEPT-90, ^{13}C-NMR DEPT-135, FT-IR, ESI^{+}-MS, COSY-NMR, HMBC-NMR, HSQC-NMR, NOESY-NMR Spectra of Compounds **1-7** **S2-S36**

Compound 1 1H NMR

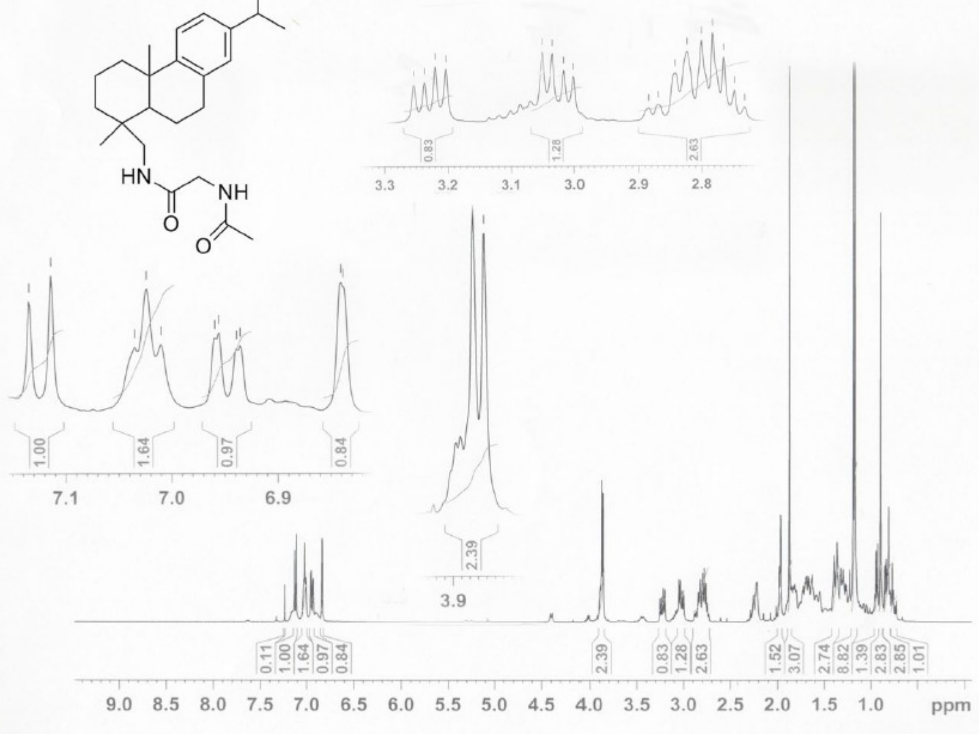

Compound 1 13C BB

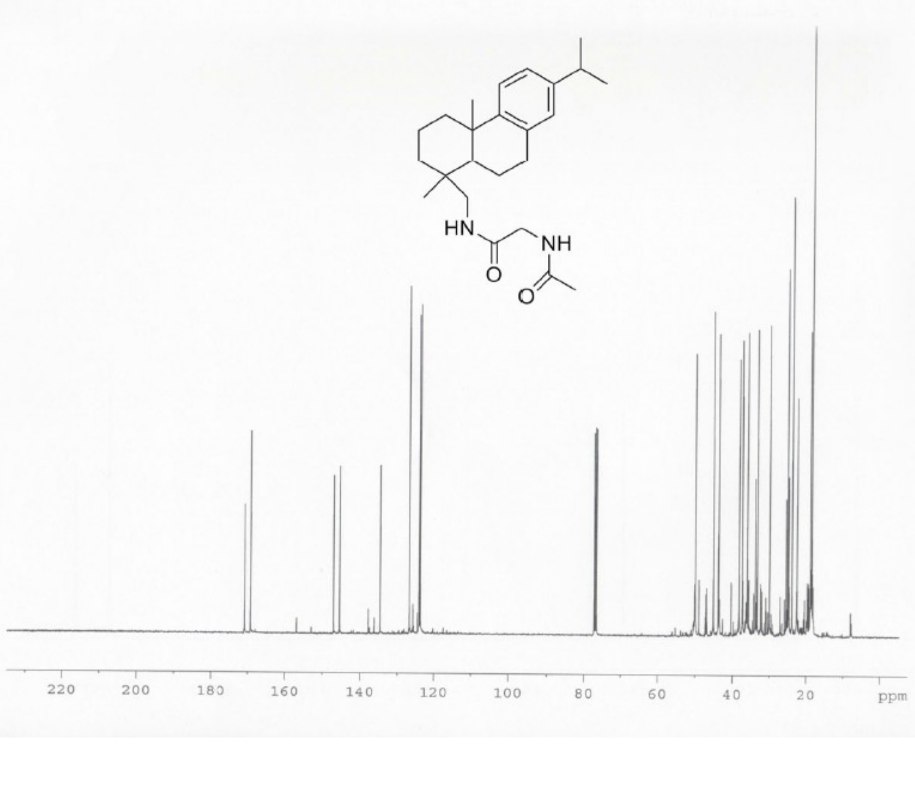

Compound 1 DEPT 90

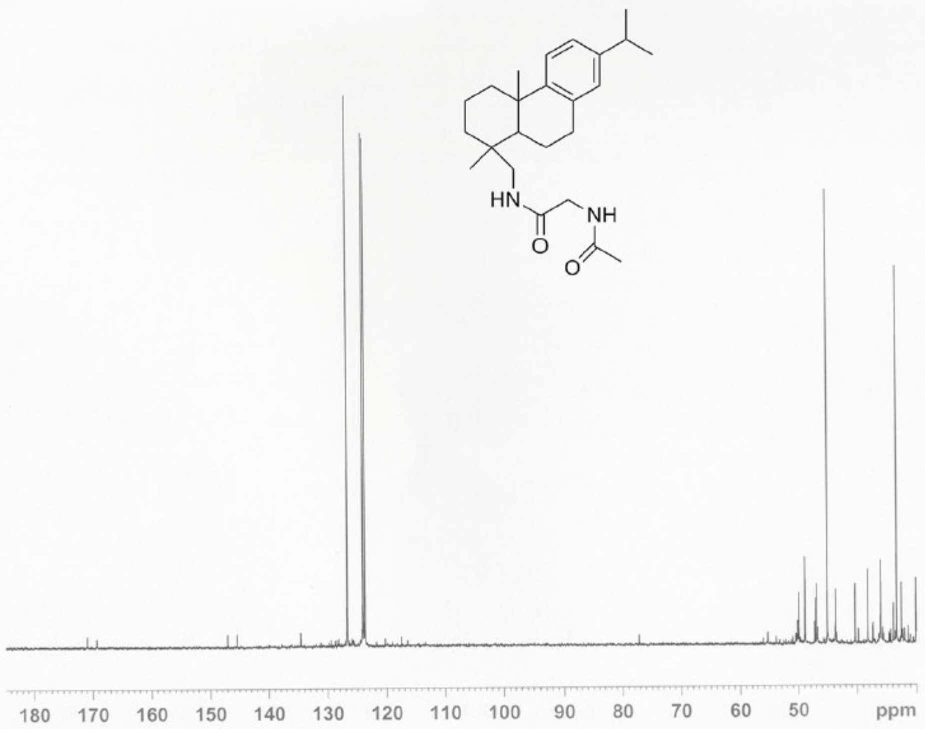

Compound 1 DEPT 135

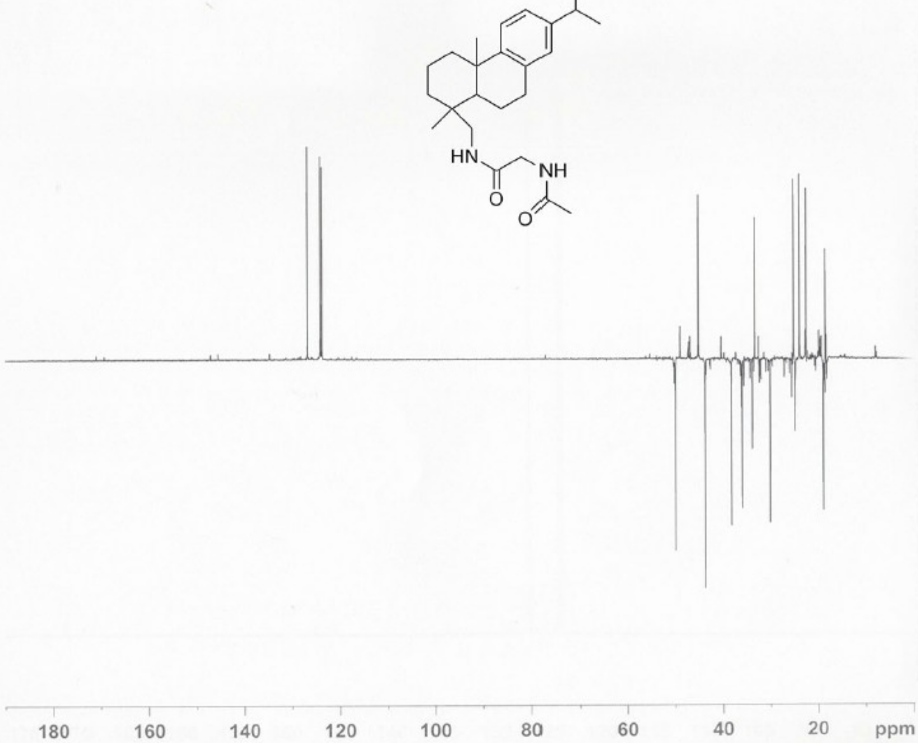

Compound 1 FTIR

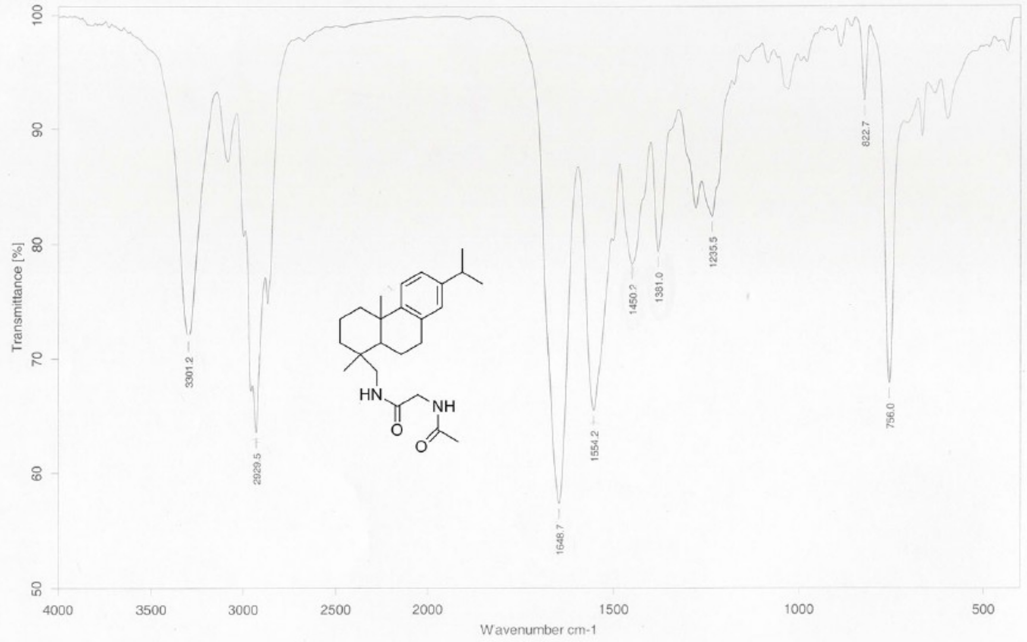

Compound 1 +TOF MS

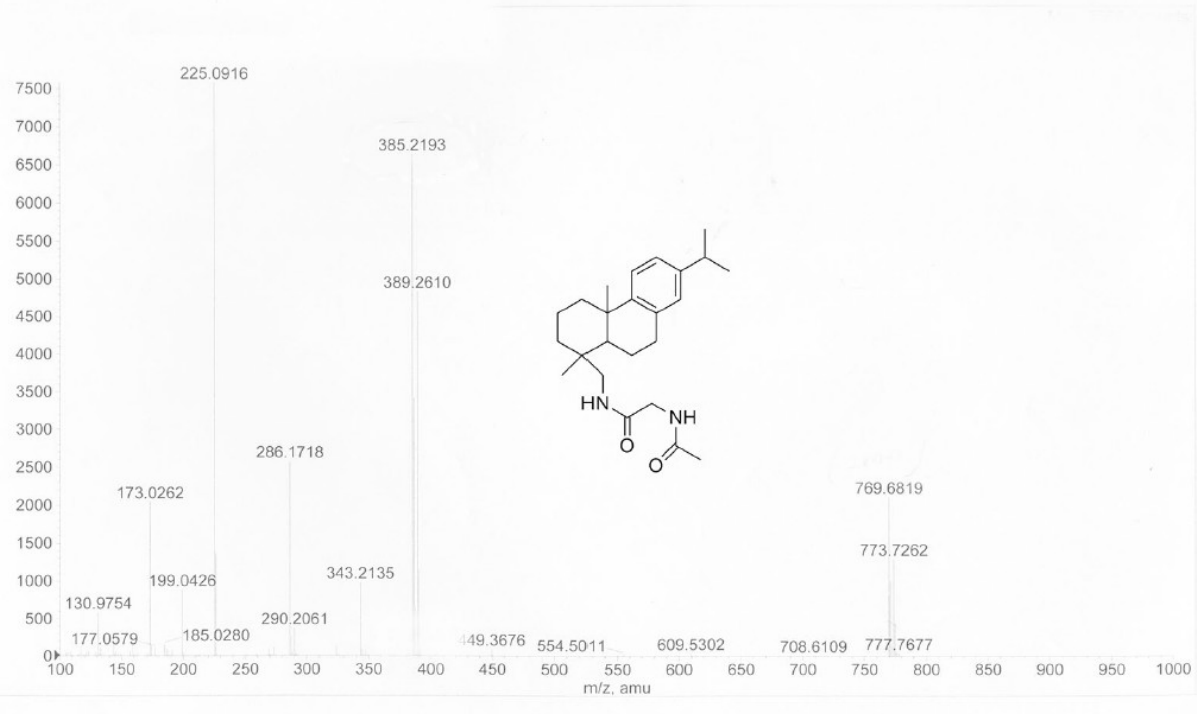

Compound 1 COSY

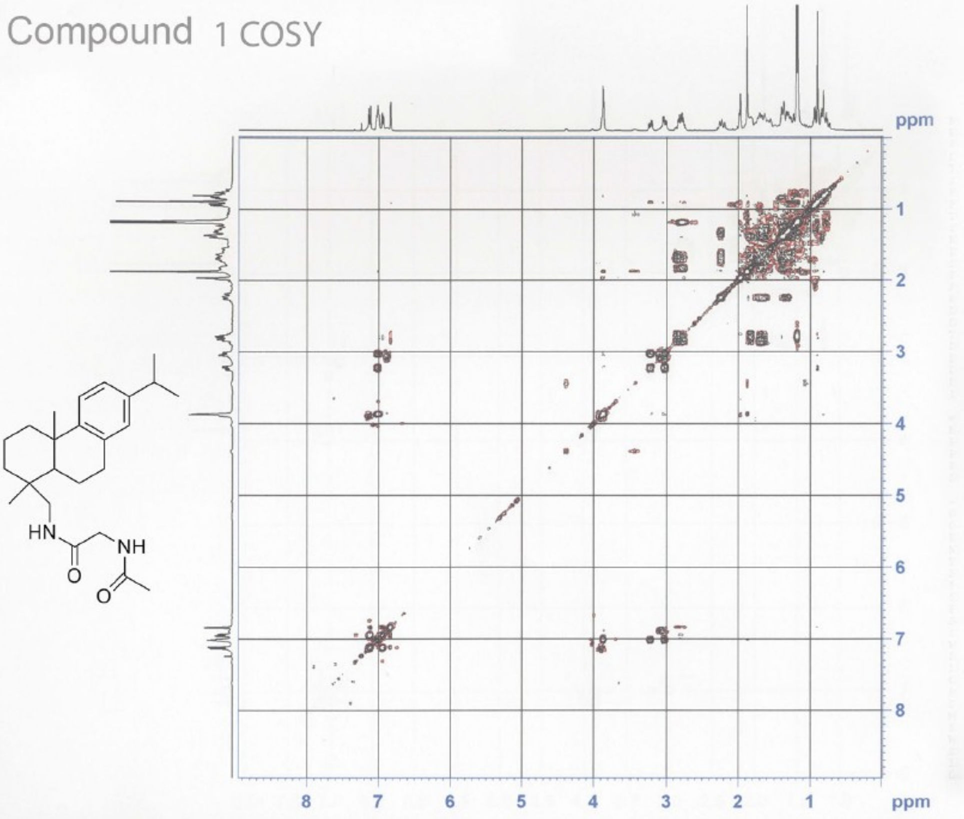

Compound 1 HMBC

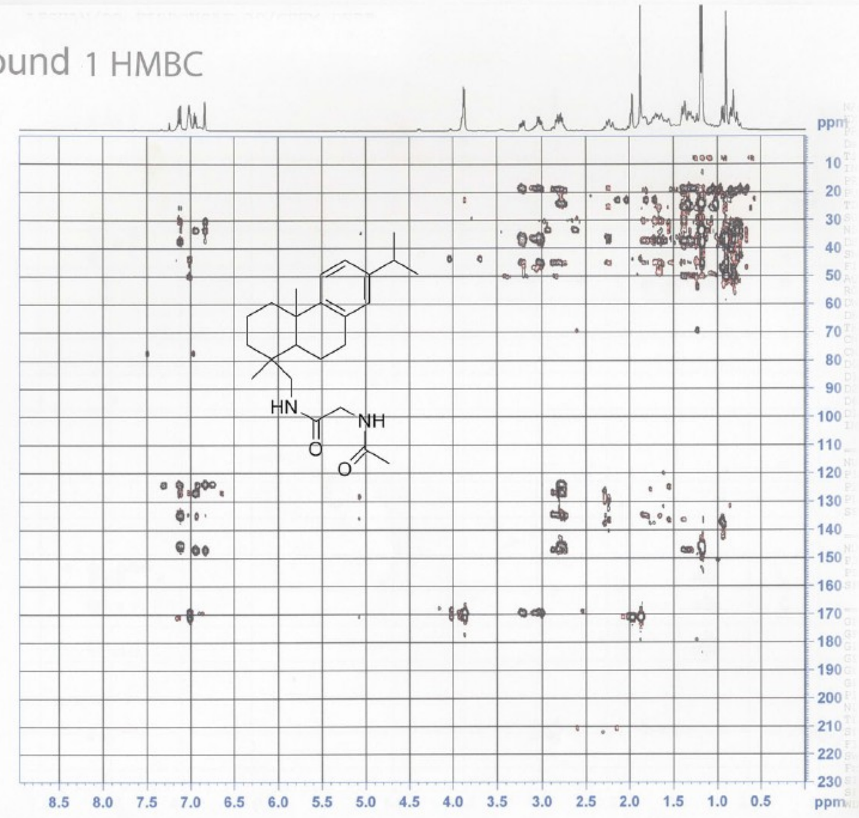

Compound 1 HSQC

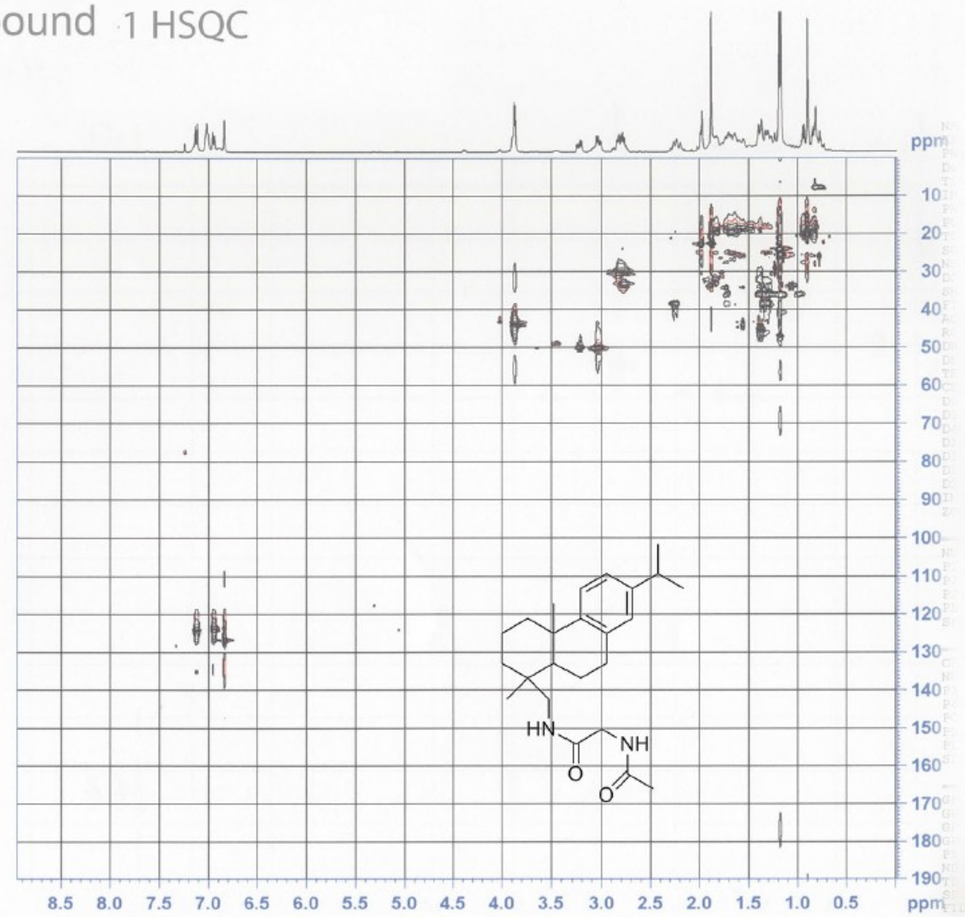

Compound 1 NOESY

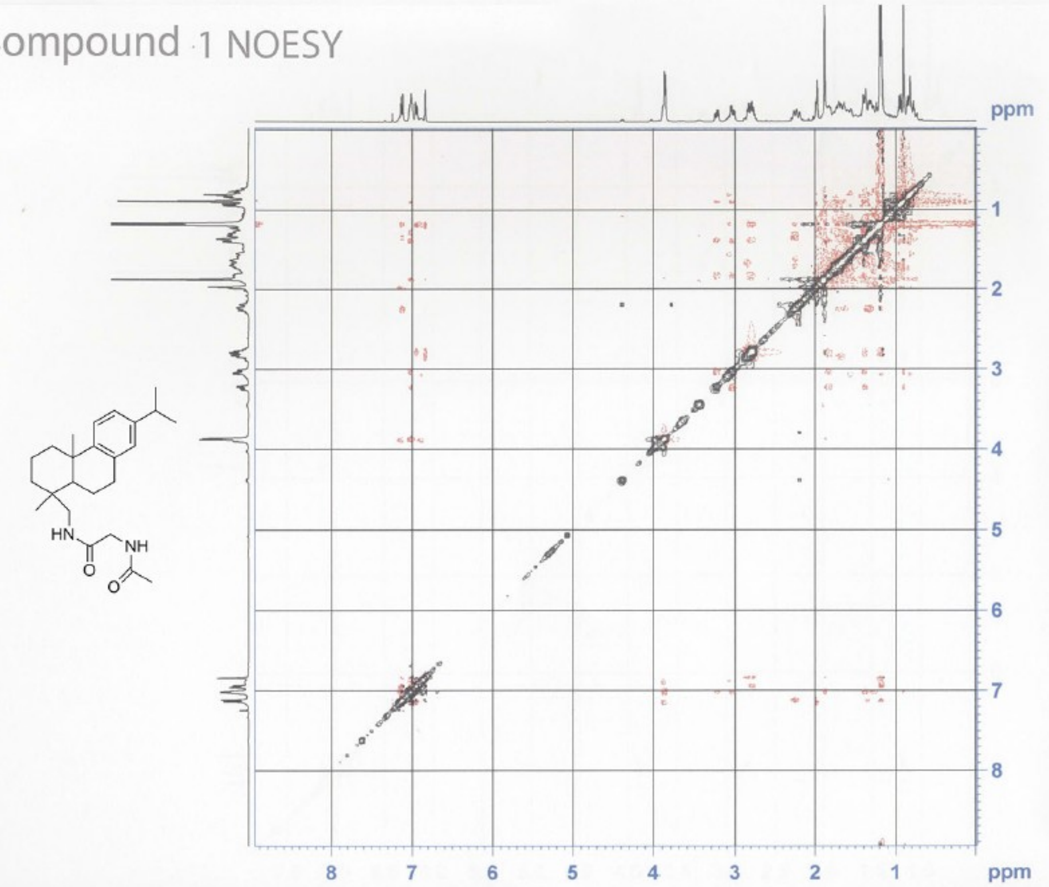

Compound 2 1H NMR

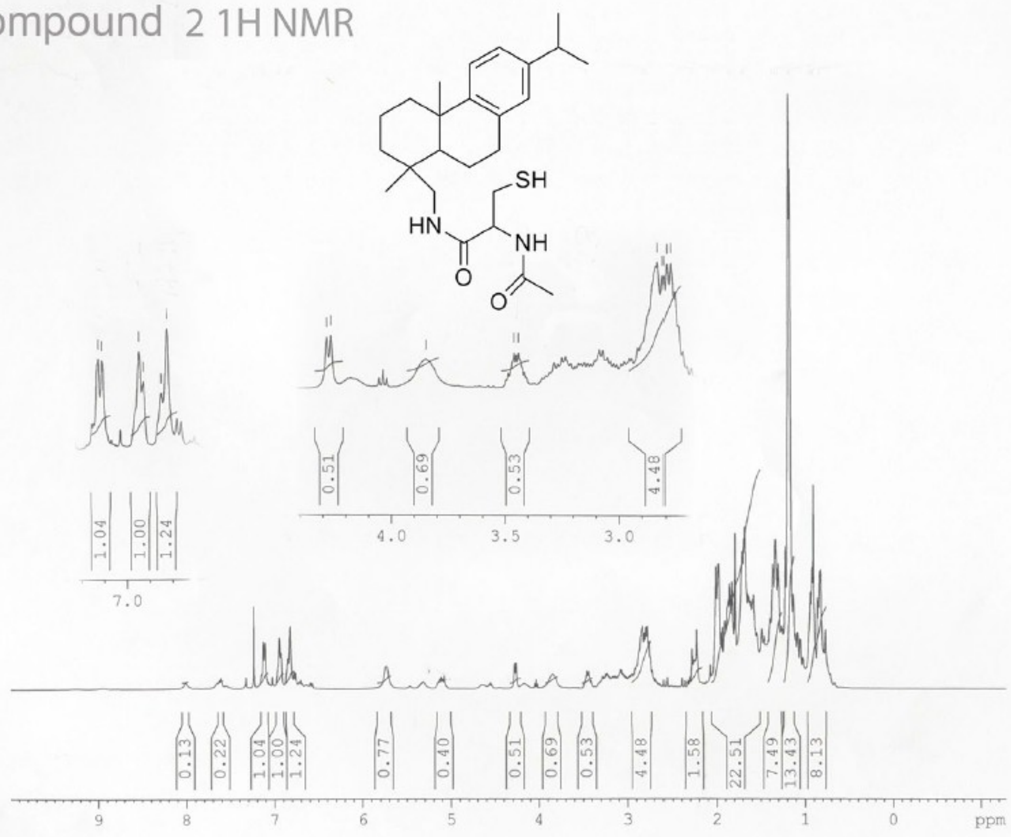

Compound 2 13C BB

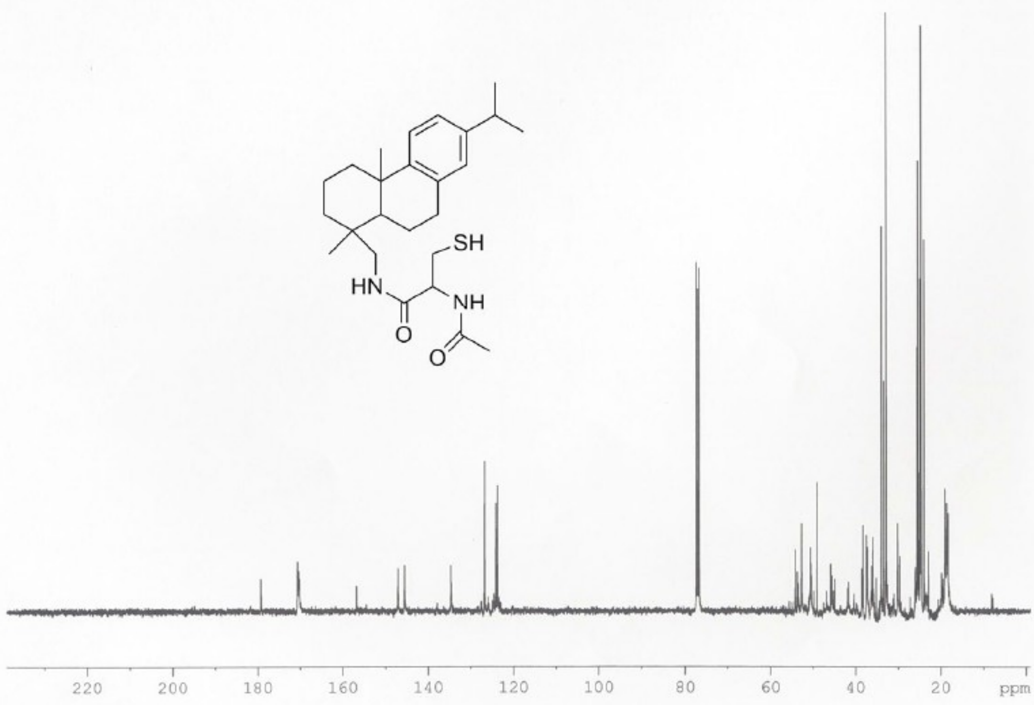

Compound -2 DEPT 90

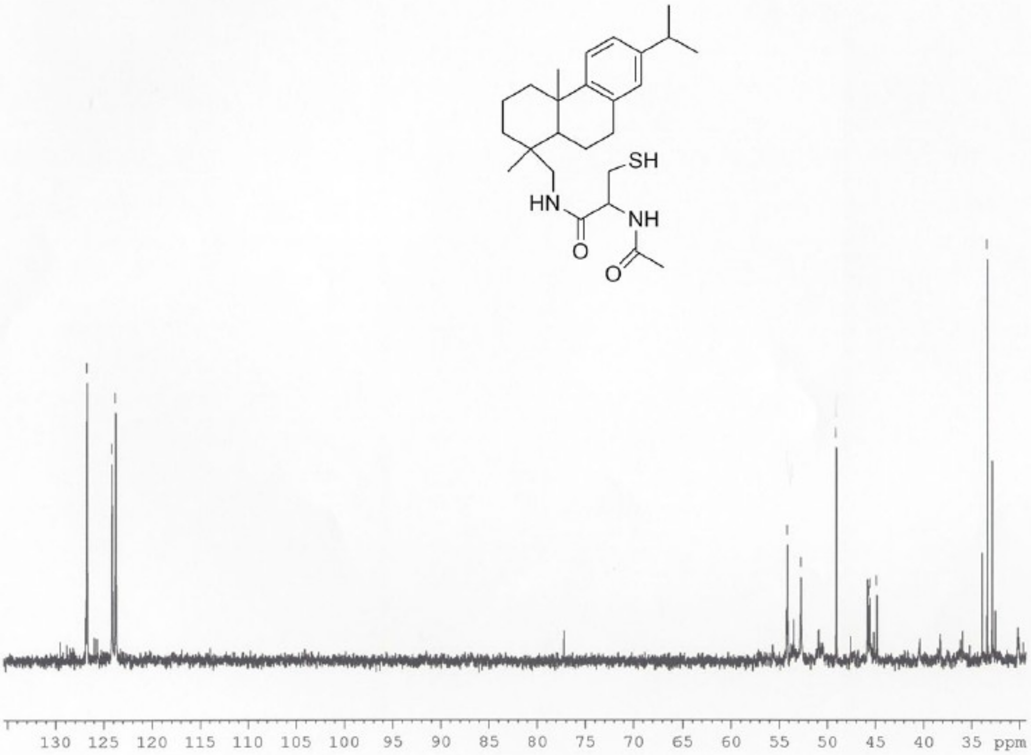

Compound-2 DEPT 135

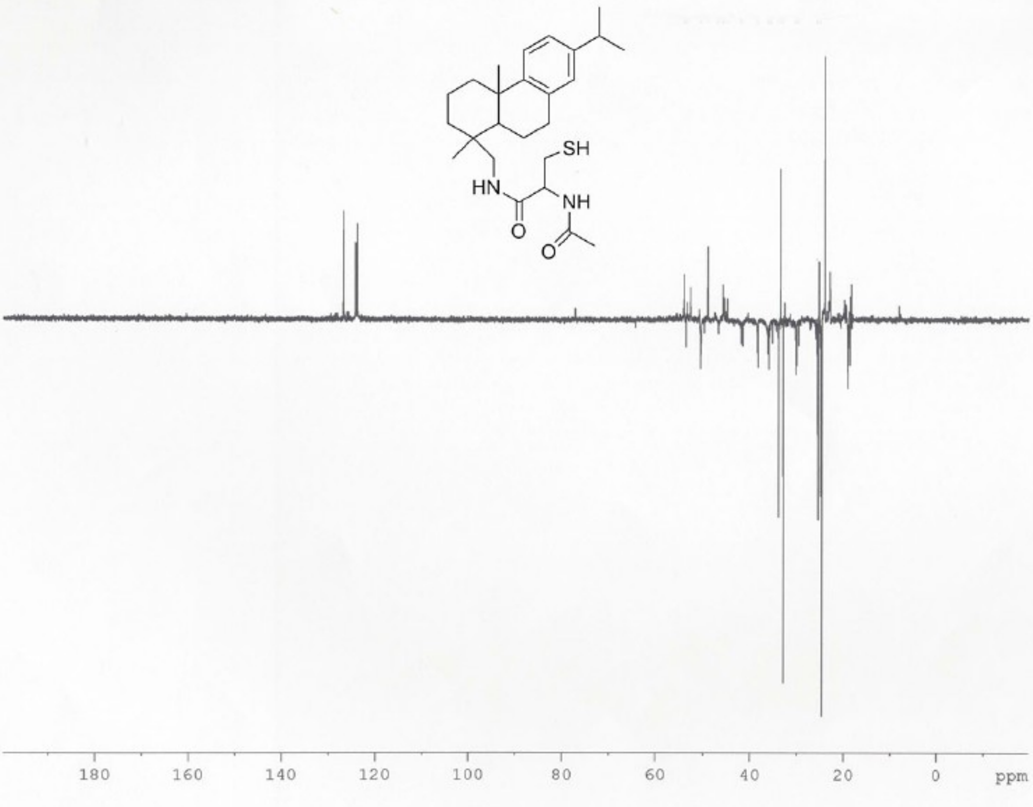

Compound -2 FTIR

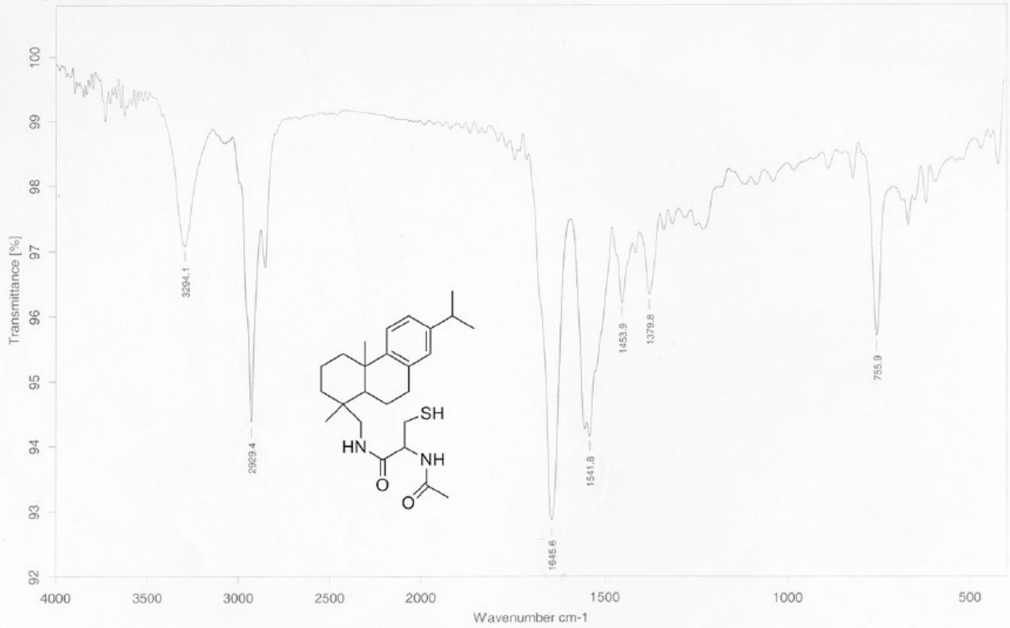

Compound -2 +TOF MS

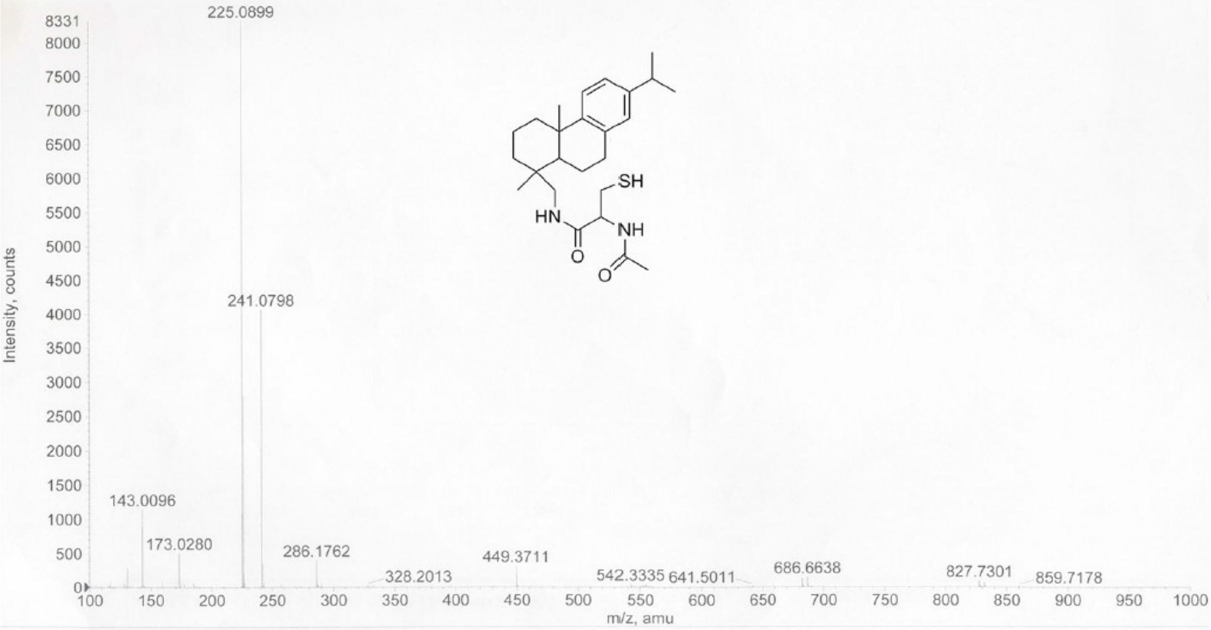

Compound 2 COSY

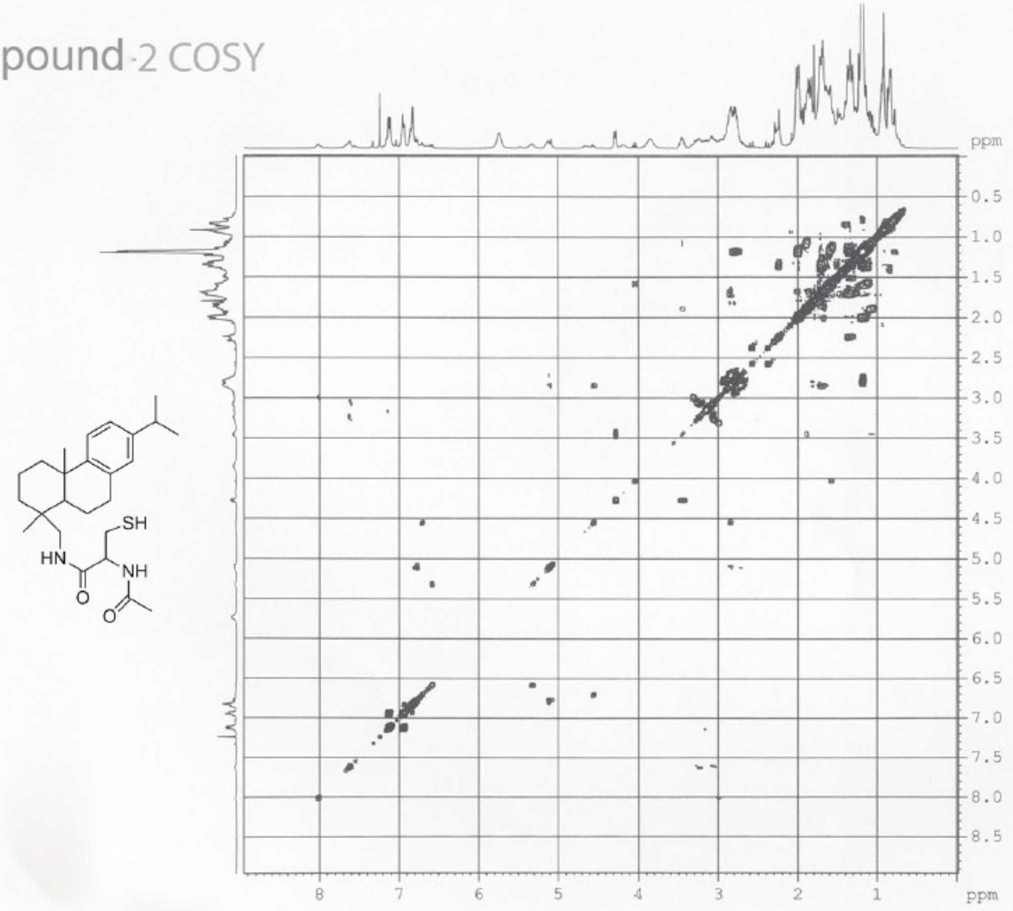

Compound 2 HMBC

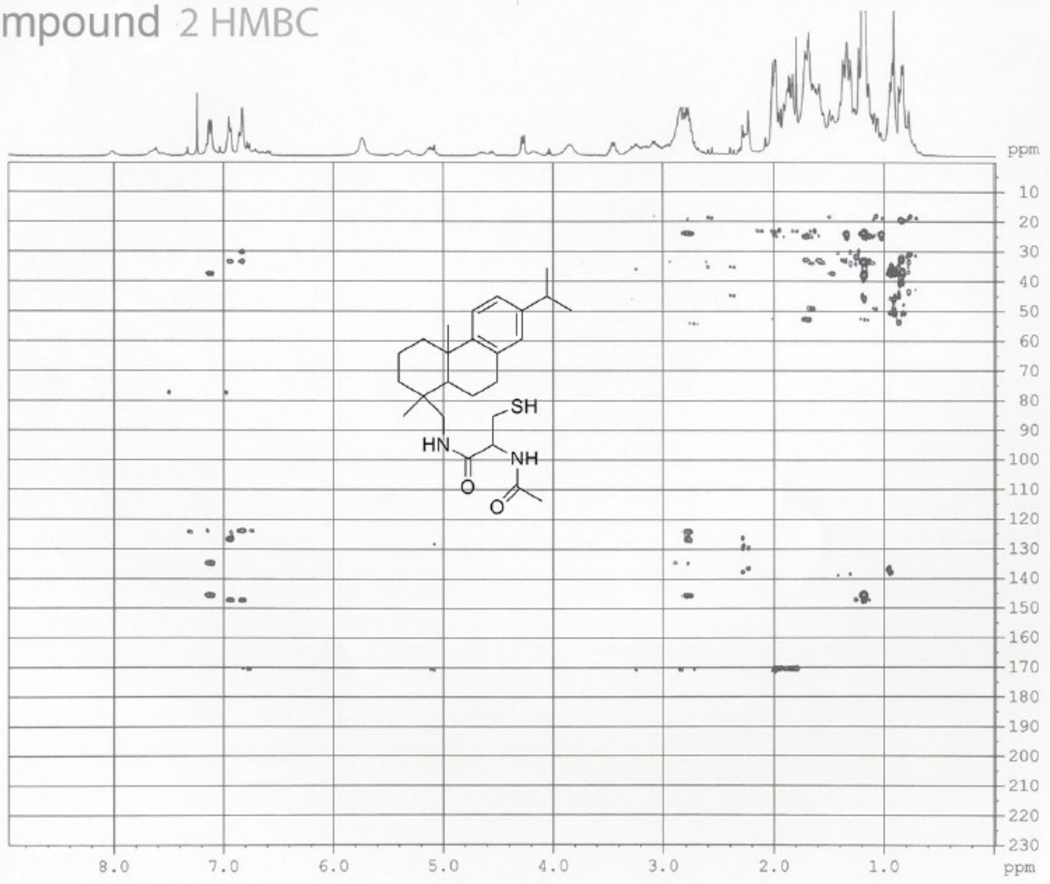

Compound 2 HSQC

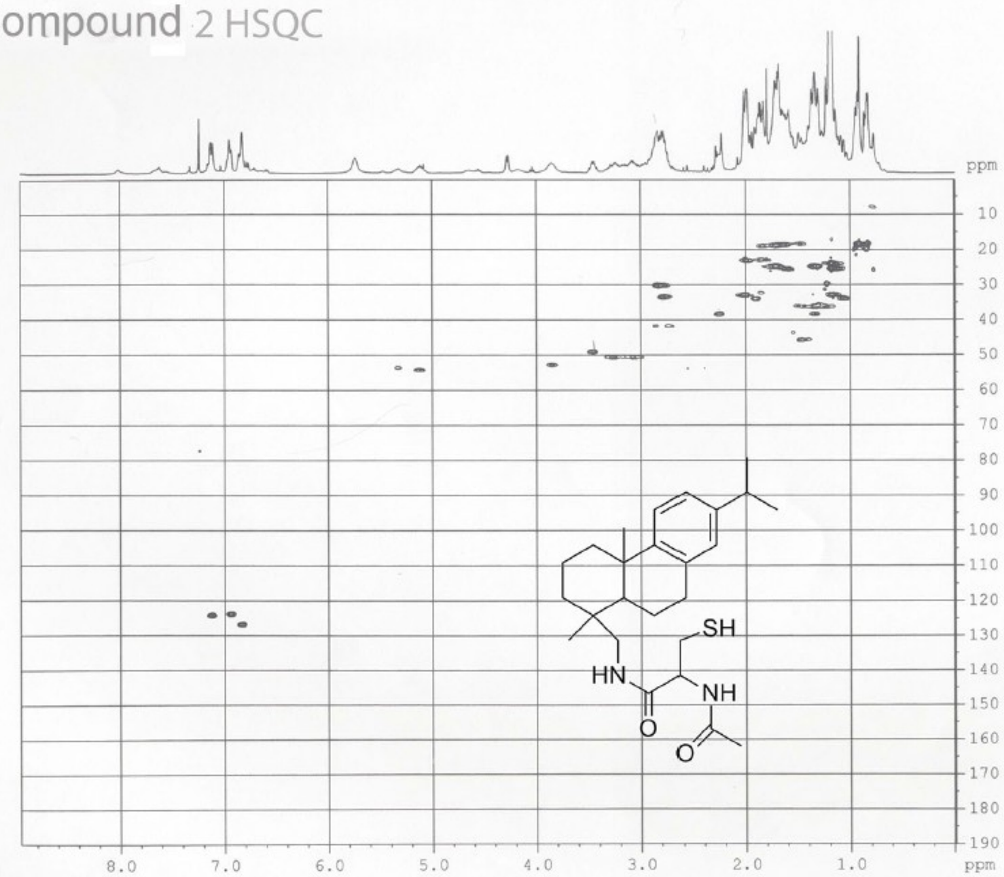

Compound 2 NOESY

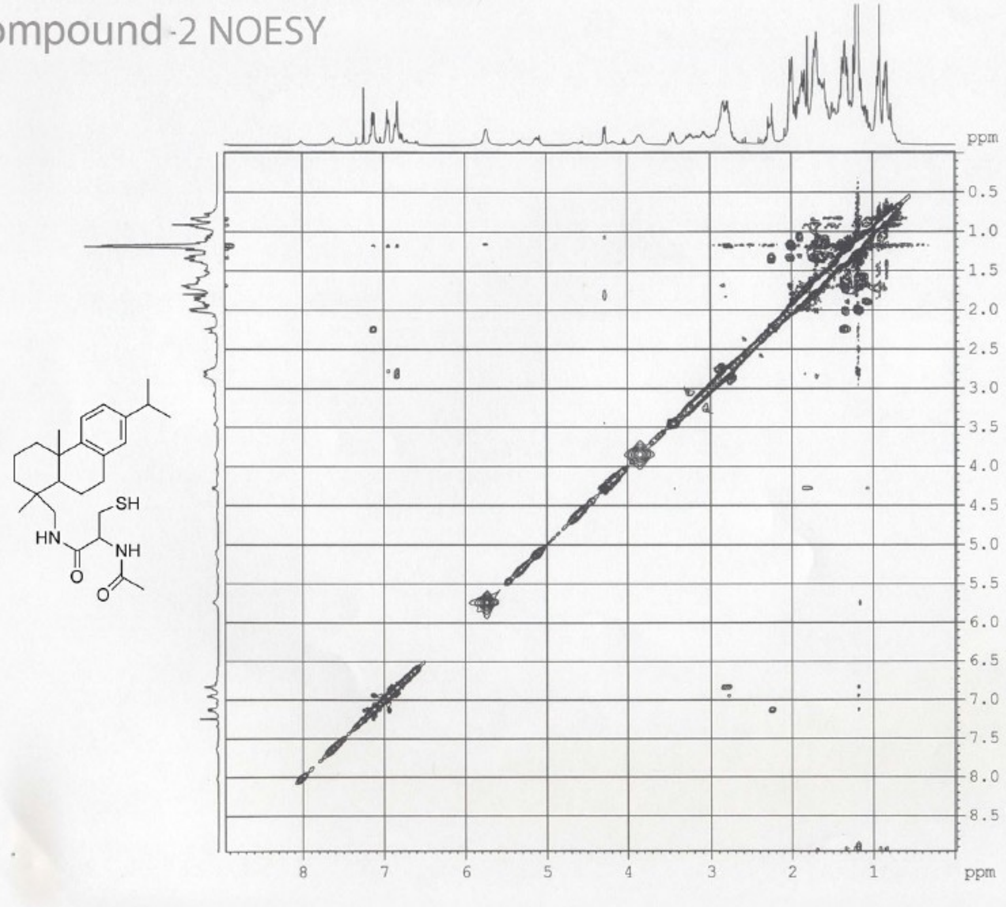

Compound 3 1H NMR

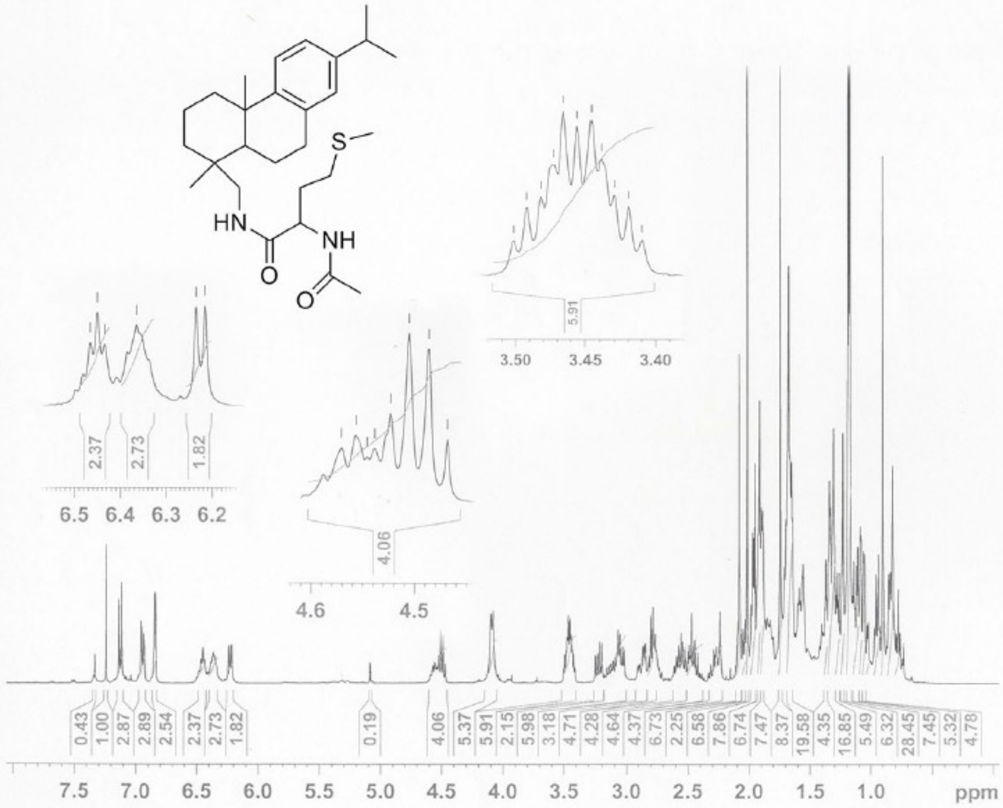

Compound 3 13C BB

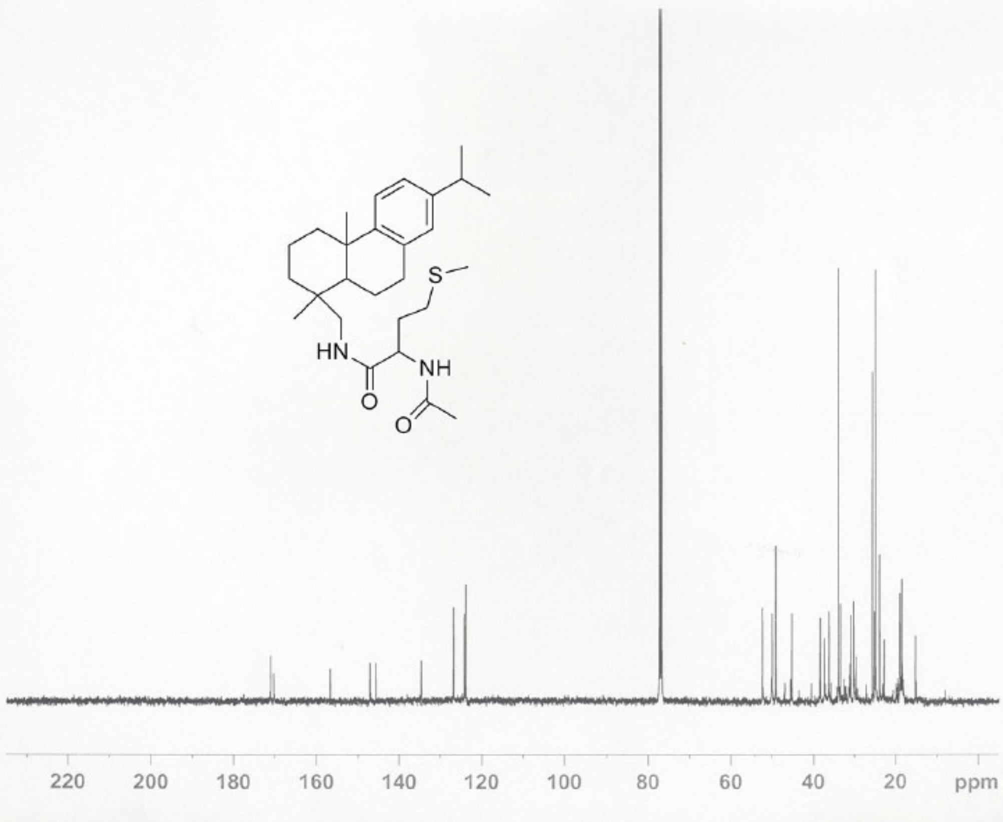

Compound 3 DEPT 90

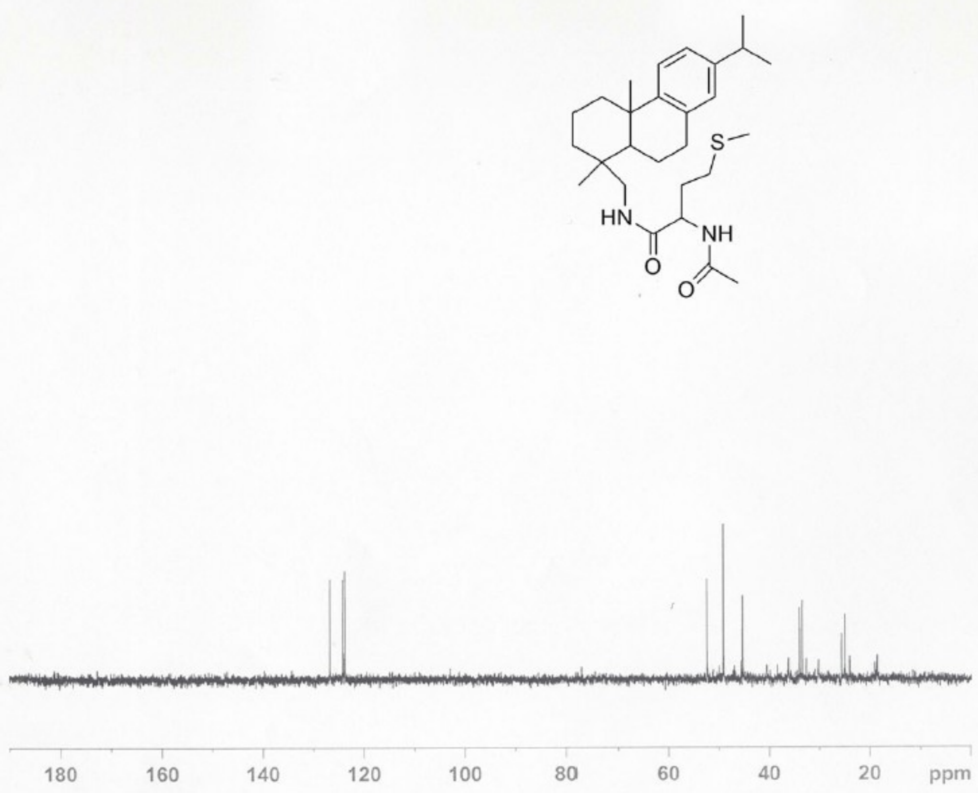

Compound 3 DEPT 135

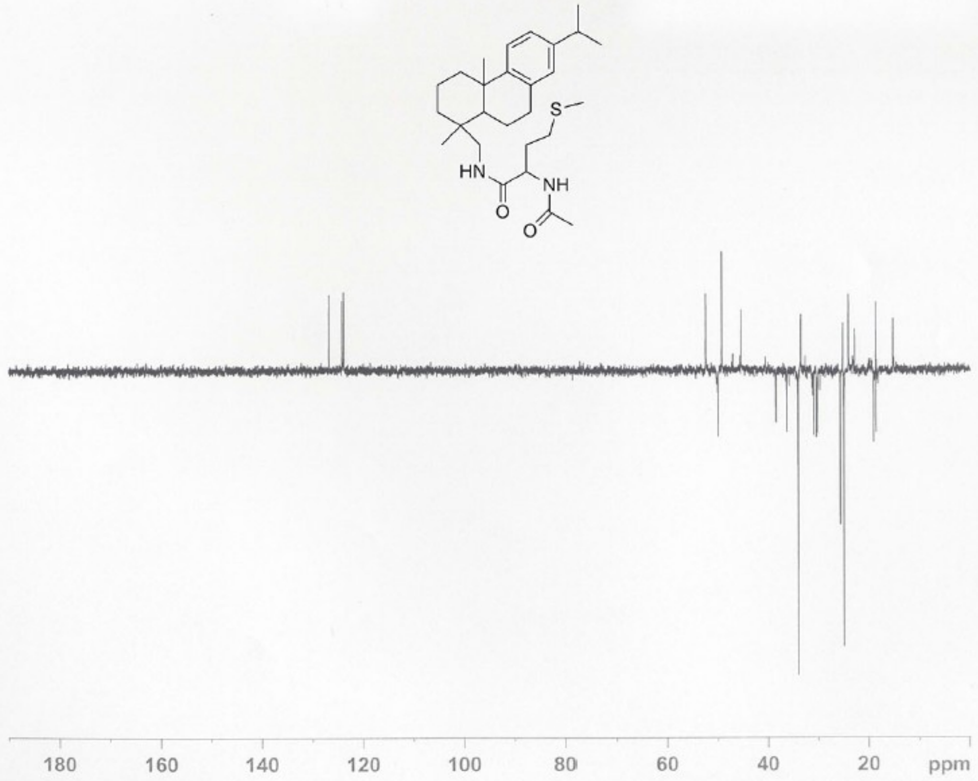

Compound 3 FTIR

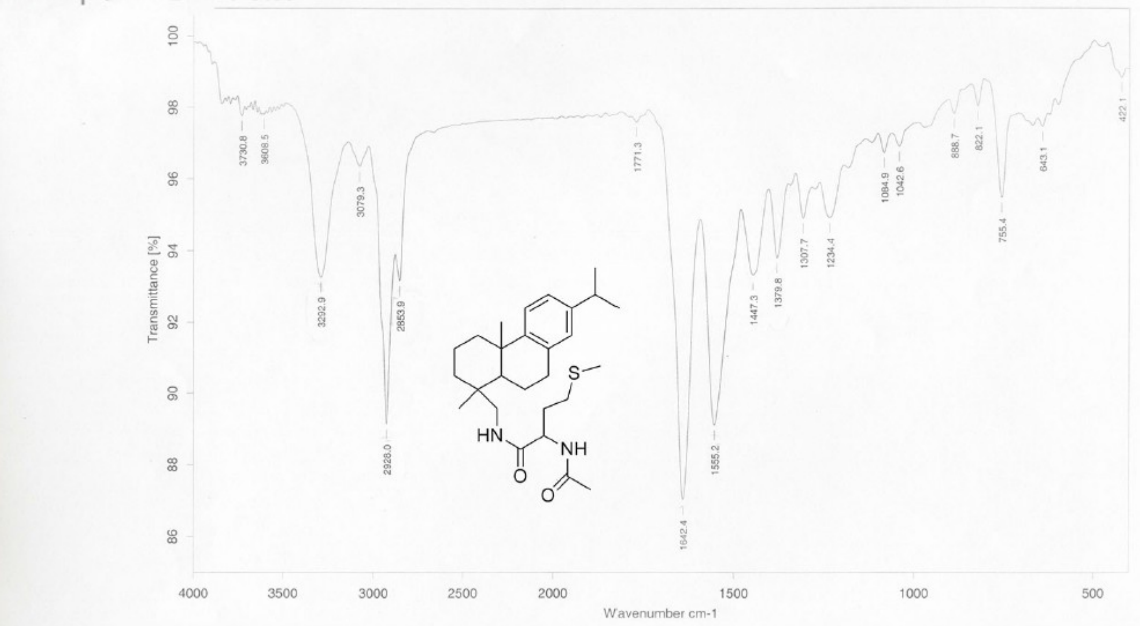

Compound 3 +TOF MS

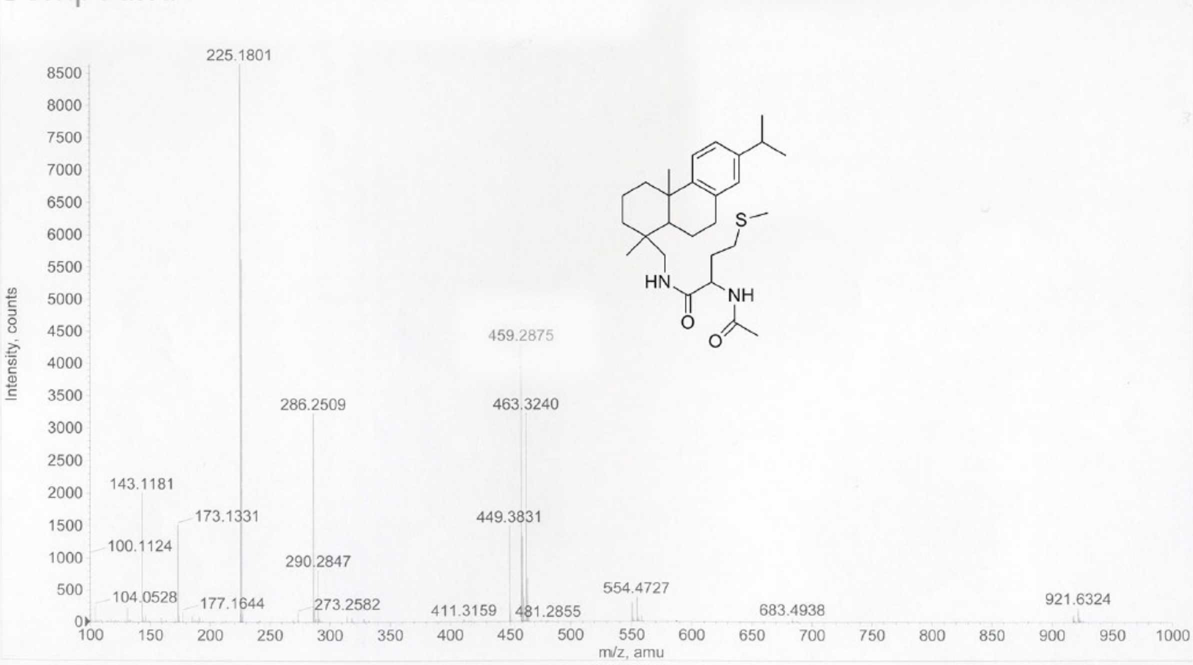

Compound 3 COSY

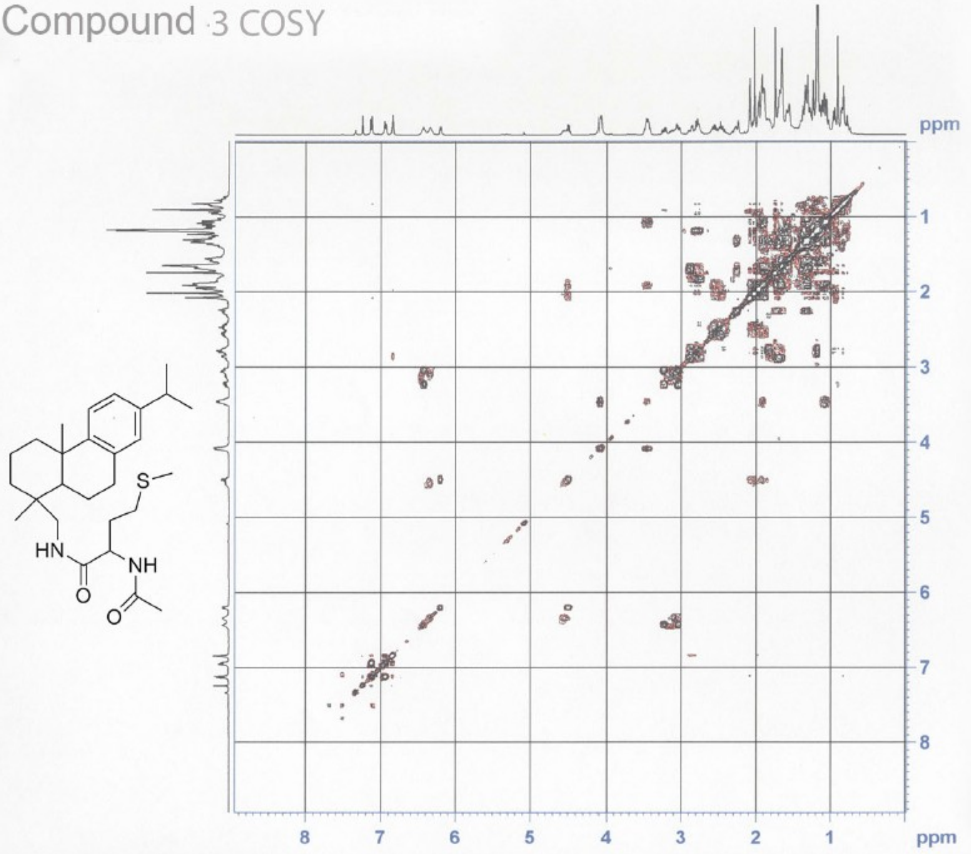

Compound 5 HMBC

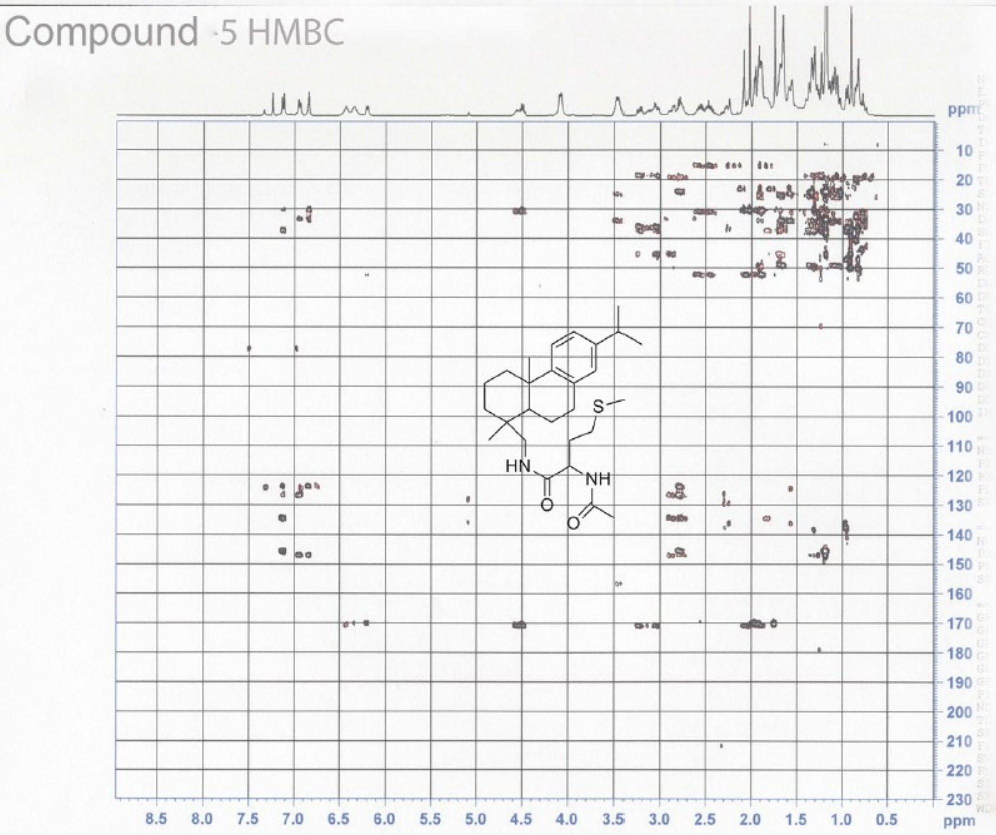

Compound-3 HSQC

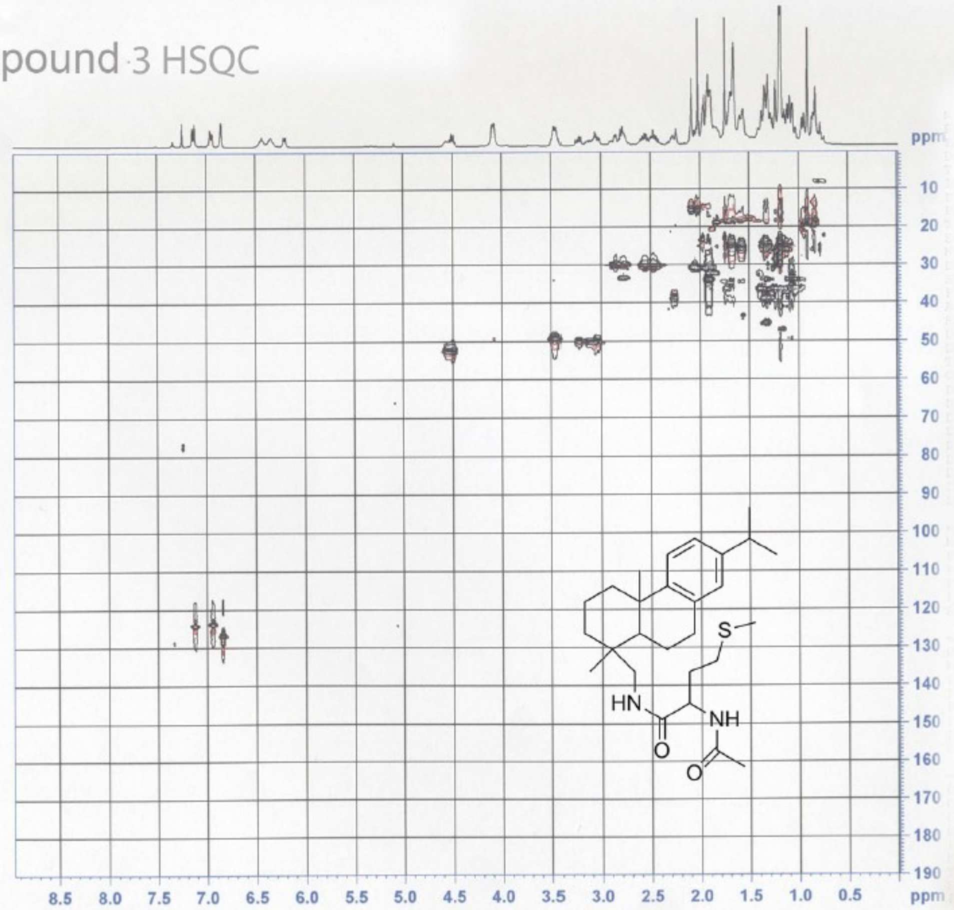

Compound-3 NOESY

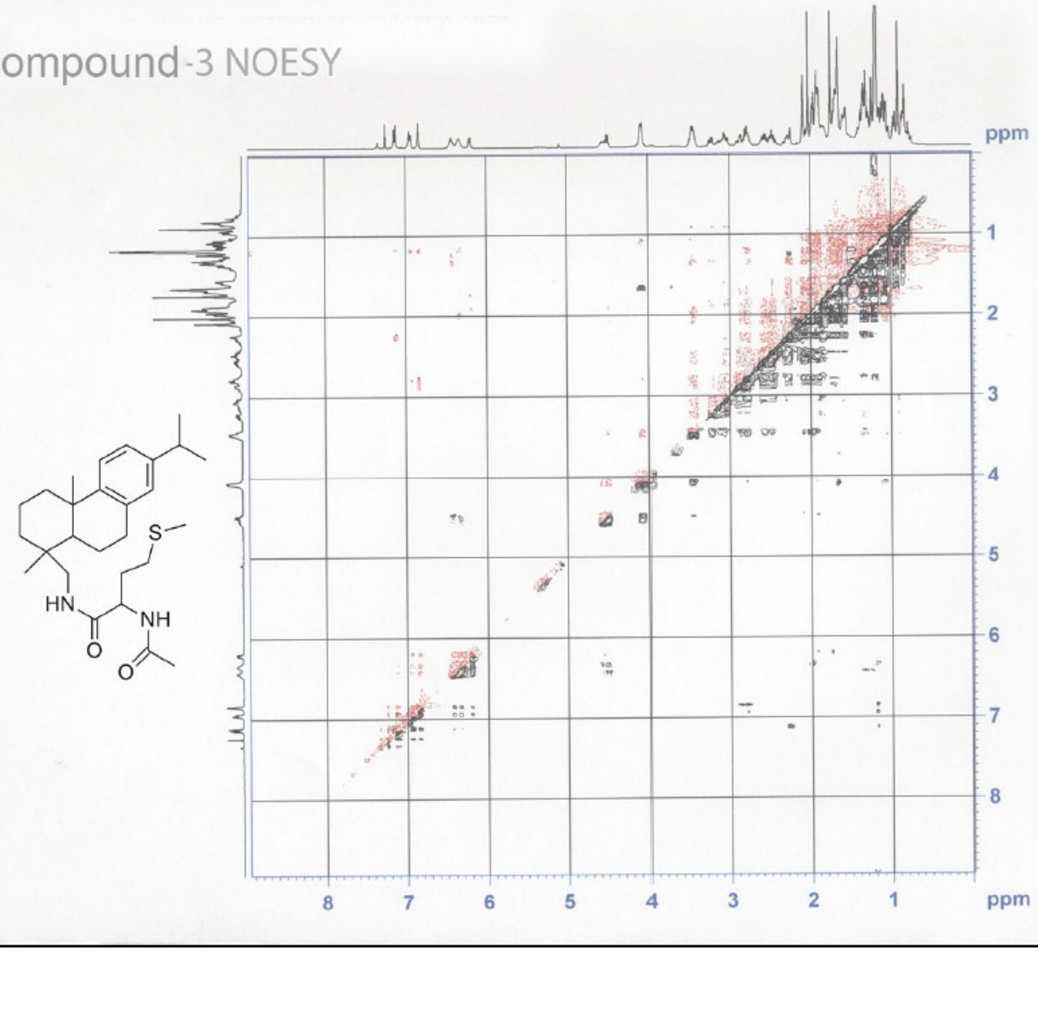

Compound 4 1H NMR

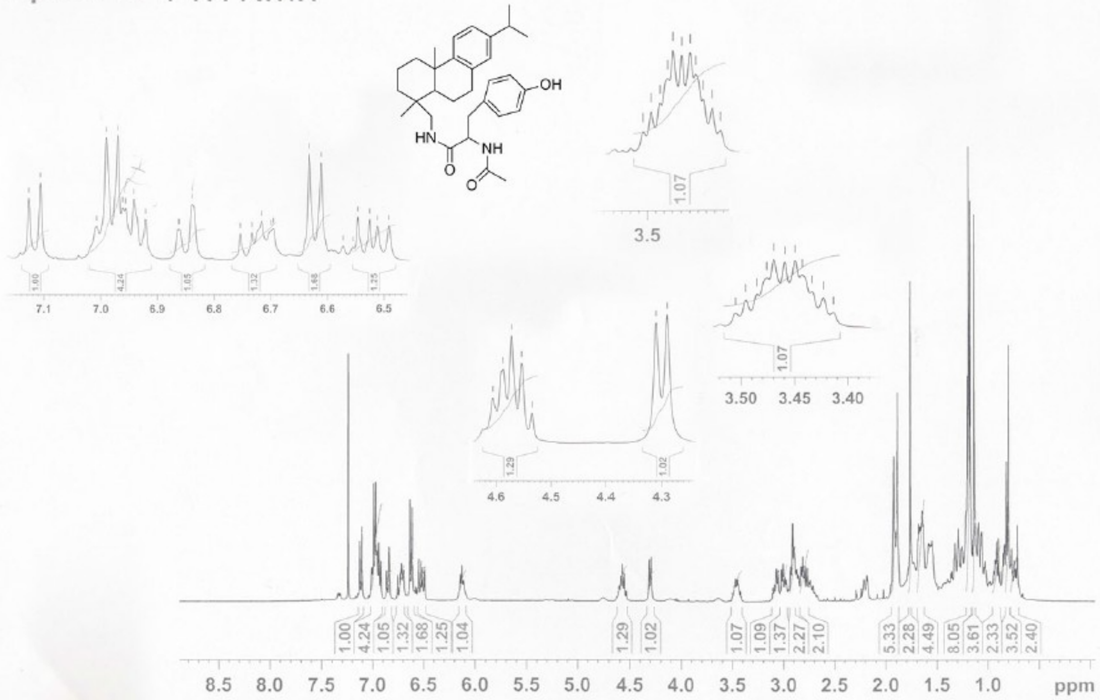

Compound 4 13C BB

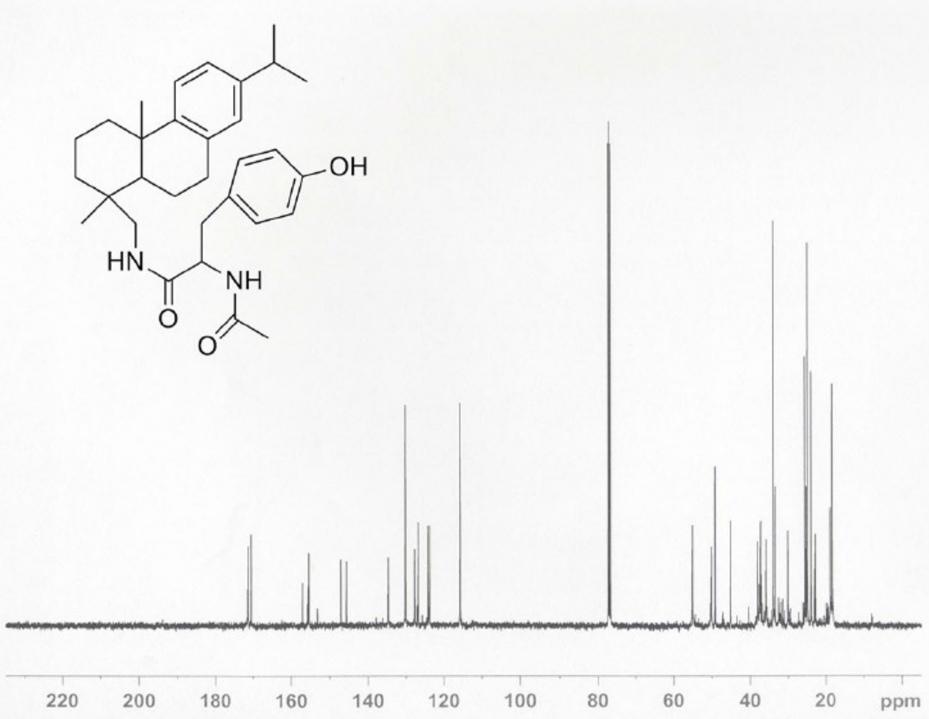

Compound 4 DEPT 90

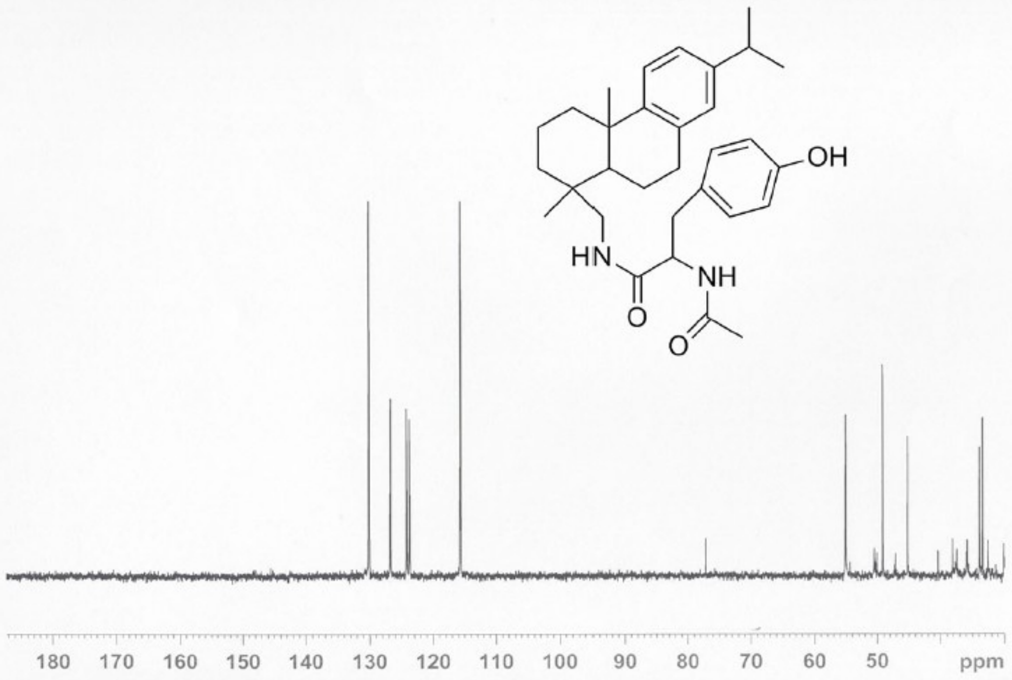

Compound 4 DEPT 135

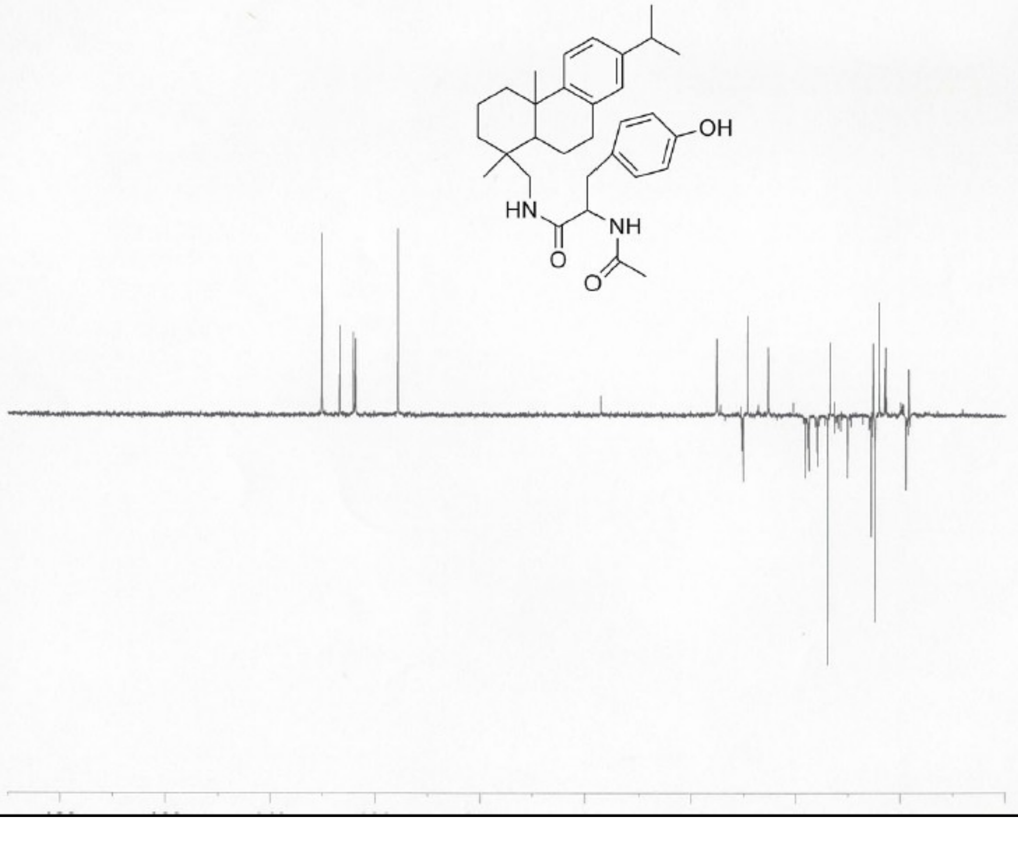

Compound -4 FTIR

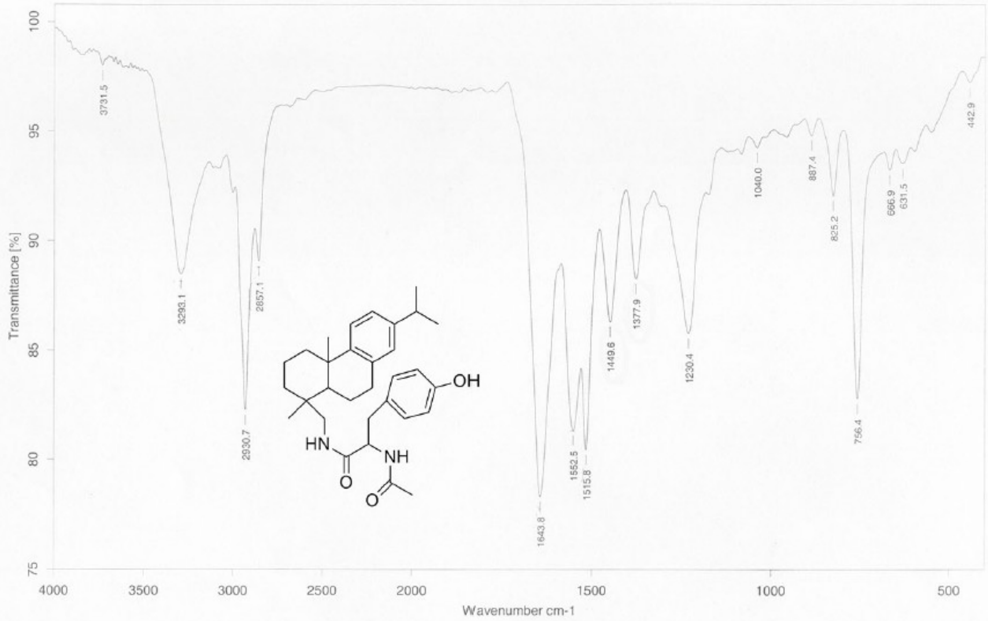

Compound -4 +TOF MS

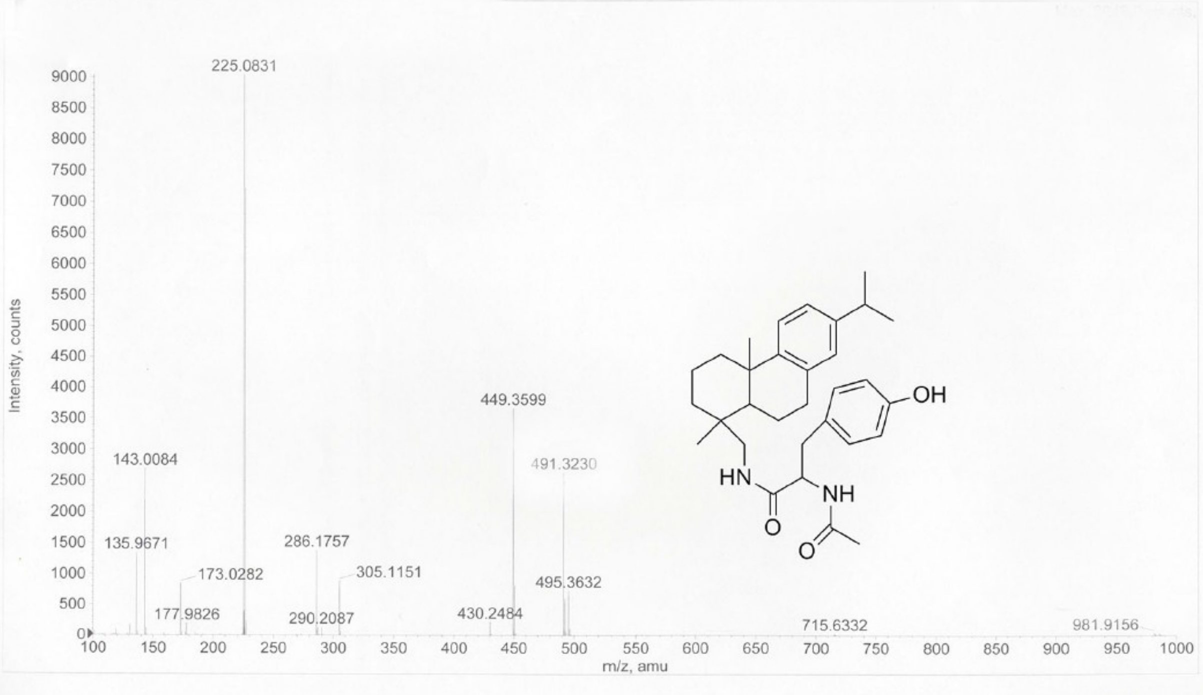

Compound 4 COSY

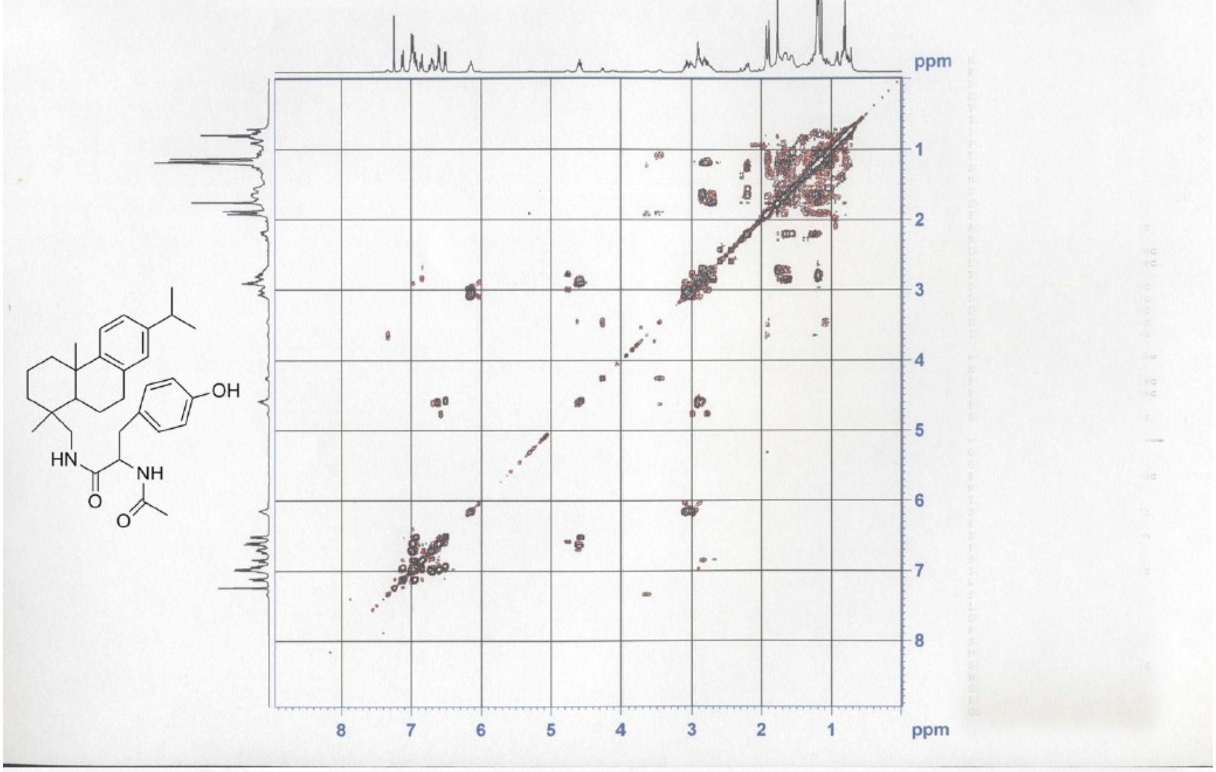

Compound 4 HMBC

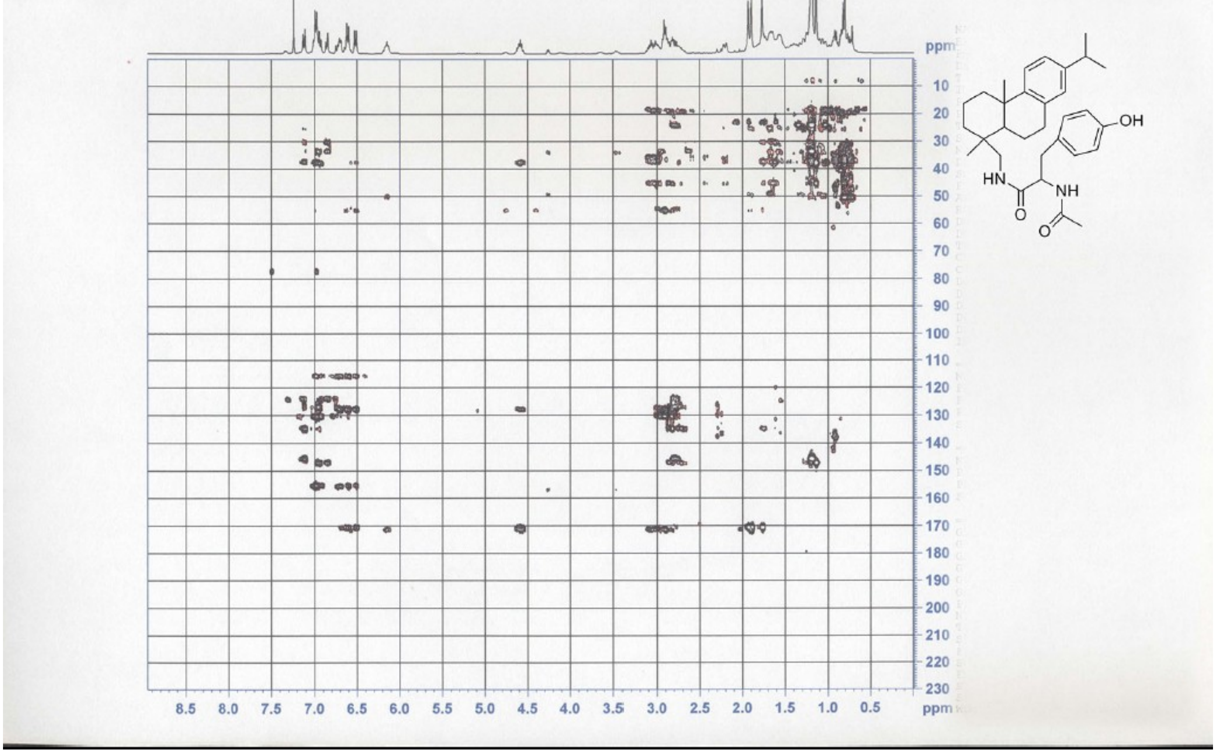

Compound -4 HSQC

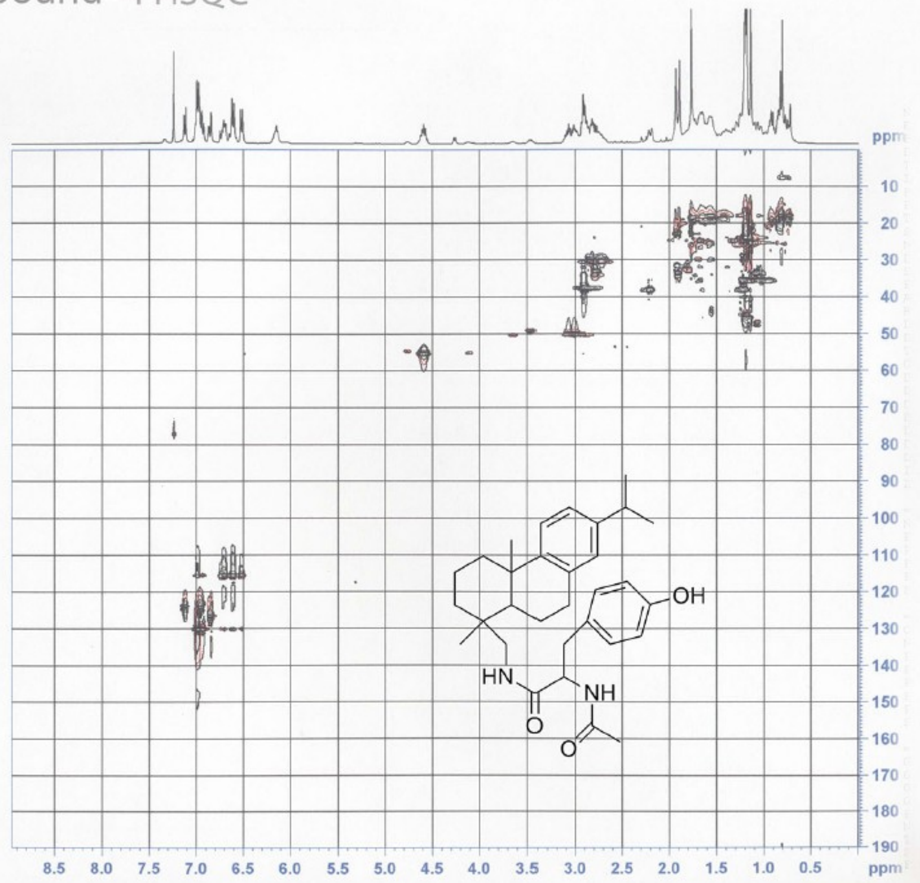

Compound 4 NOESY

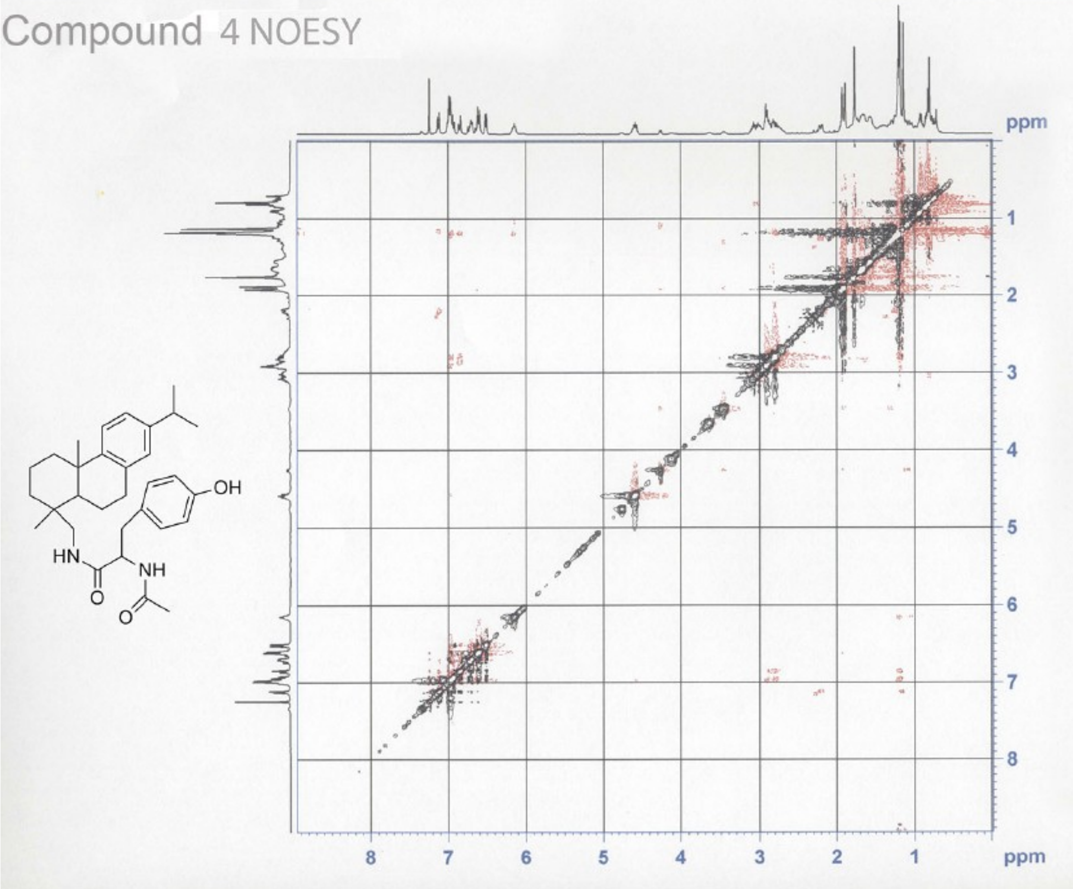

Compound 5 1H NMR

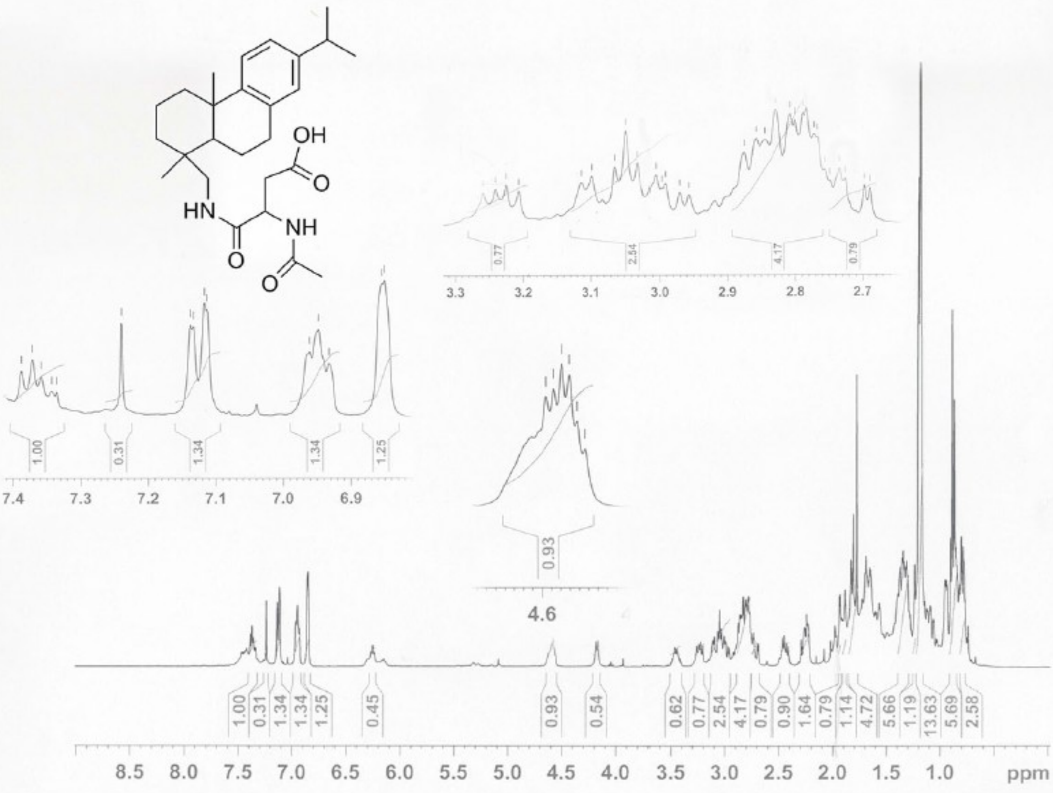

Compound 5 13C BB

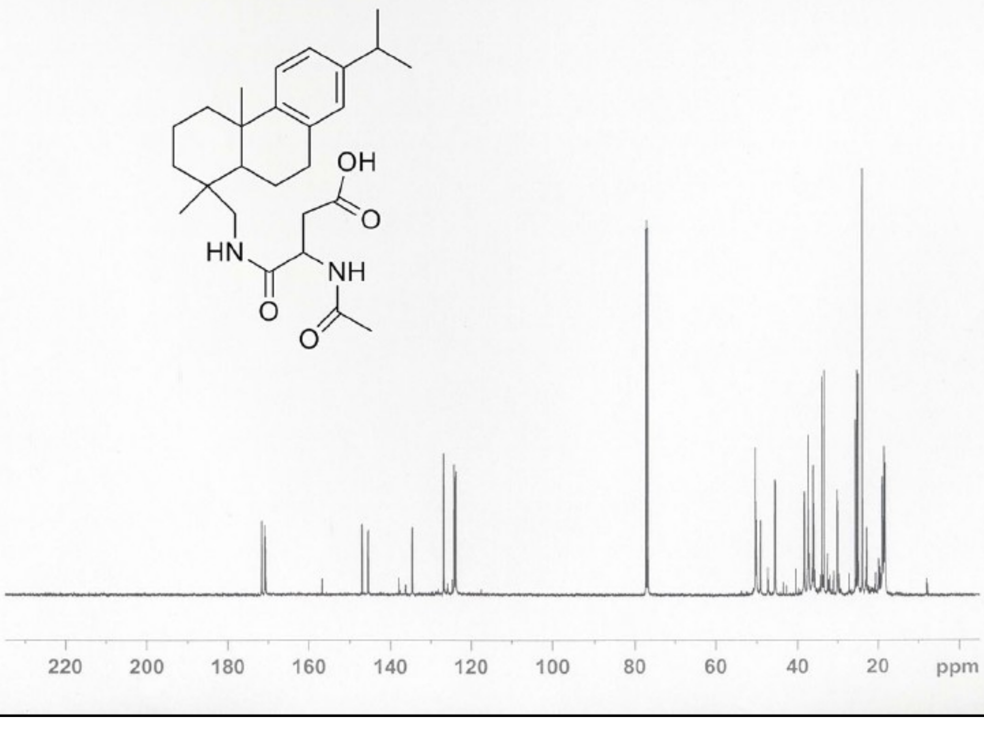

Compound -5 DEPT 90

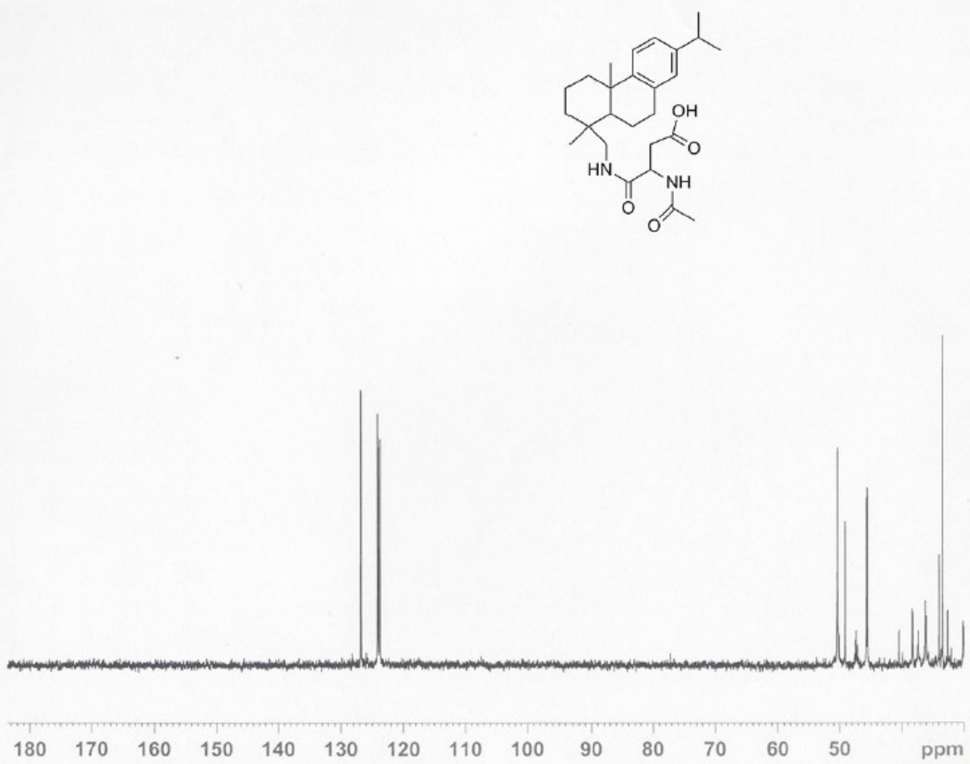

Compound 5 DEPT 135

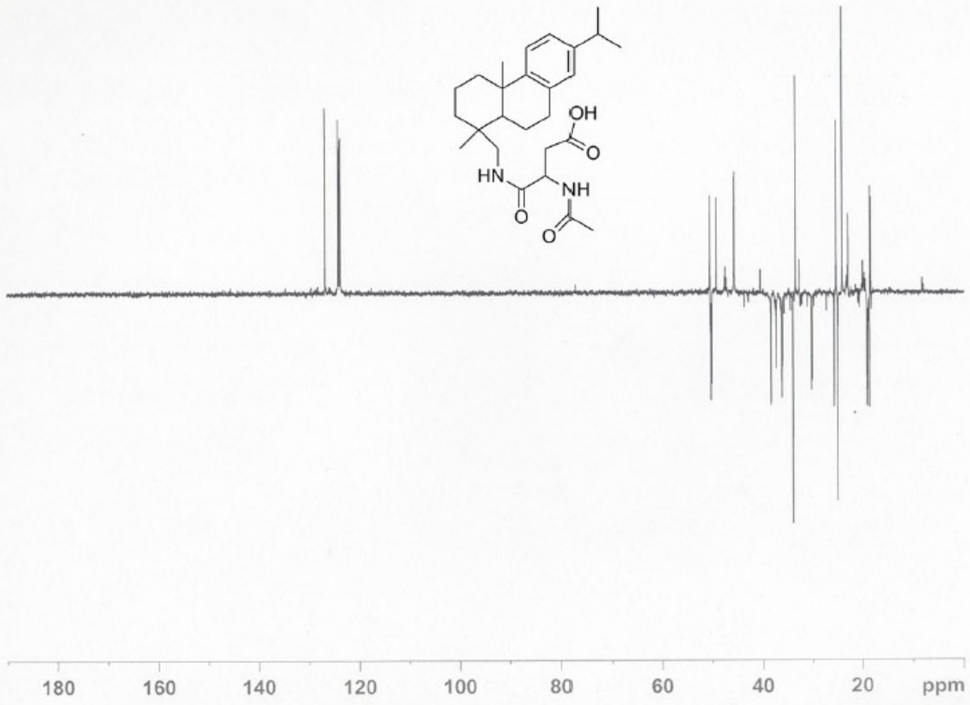

Compound 5 FTIR

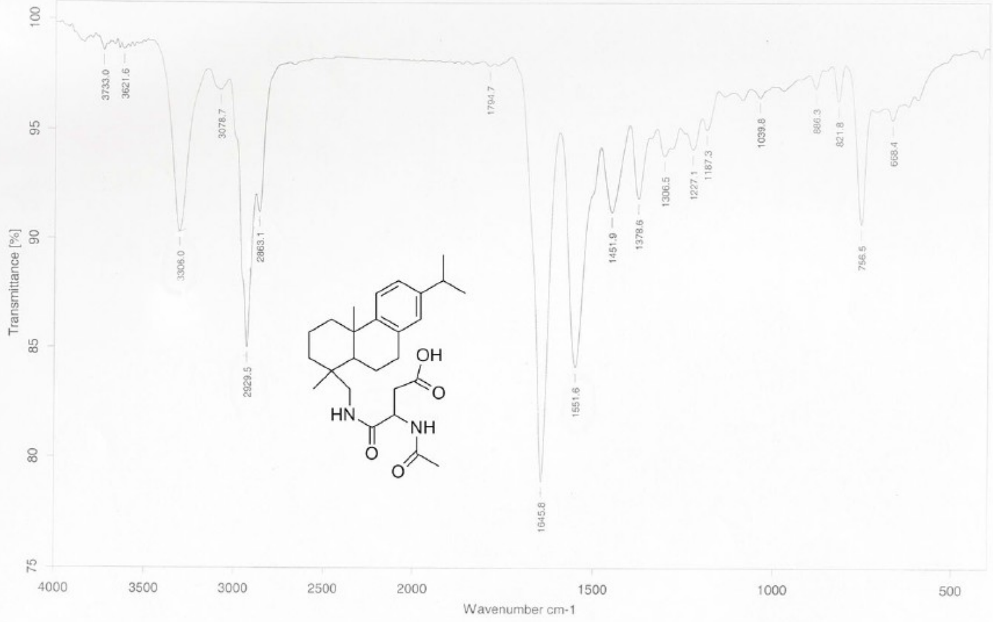

Compound 5 +TOF MS

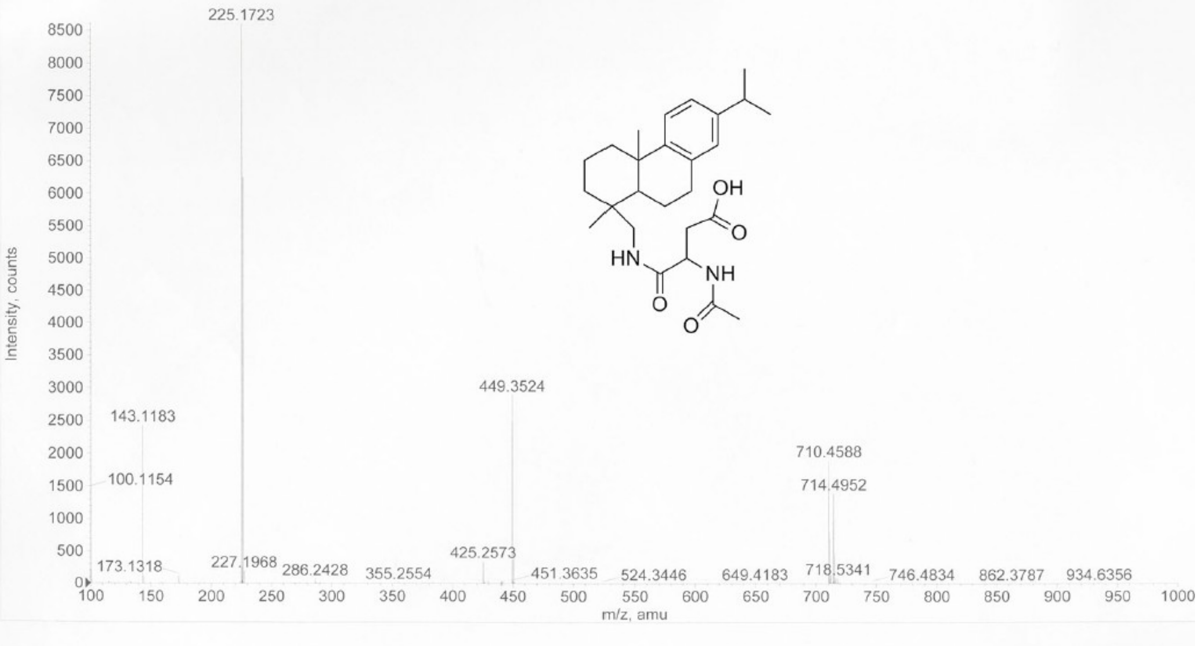

Compound 5 COSY

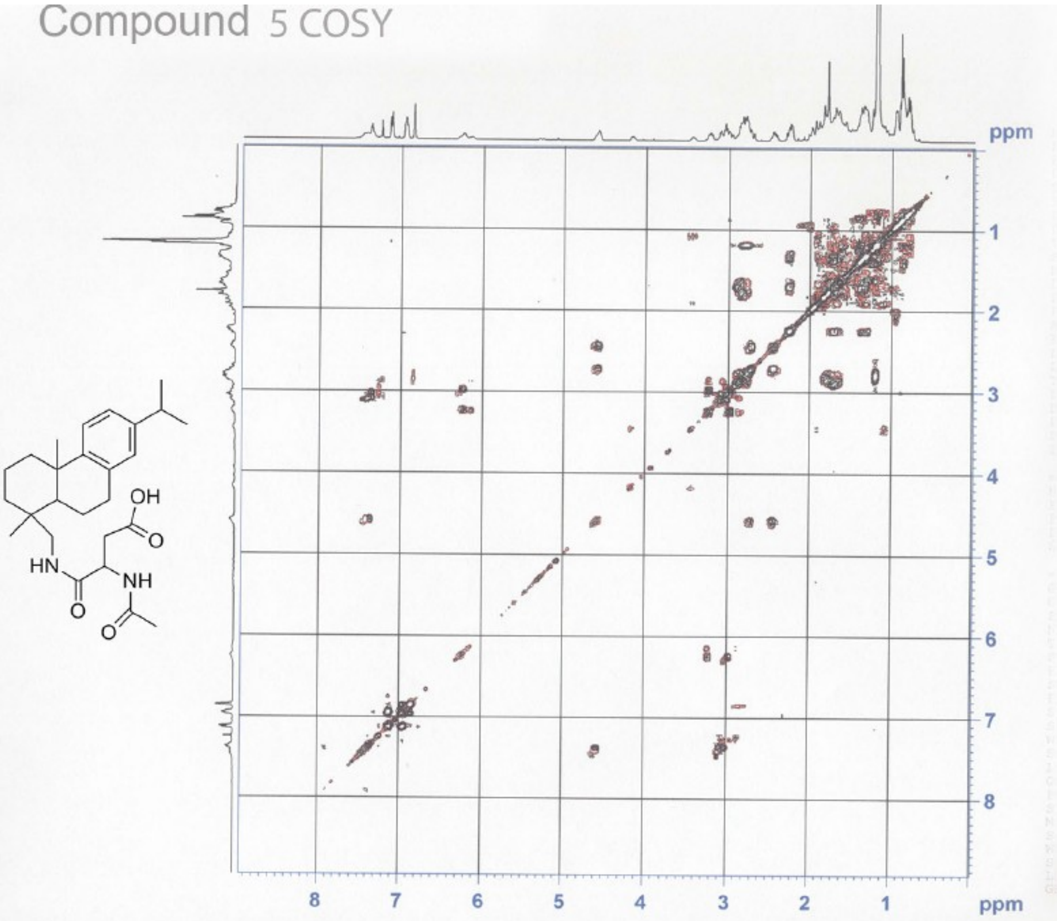

Compound 5 HMBC

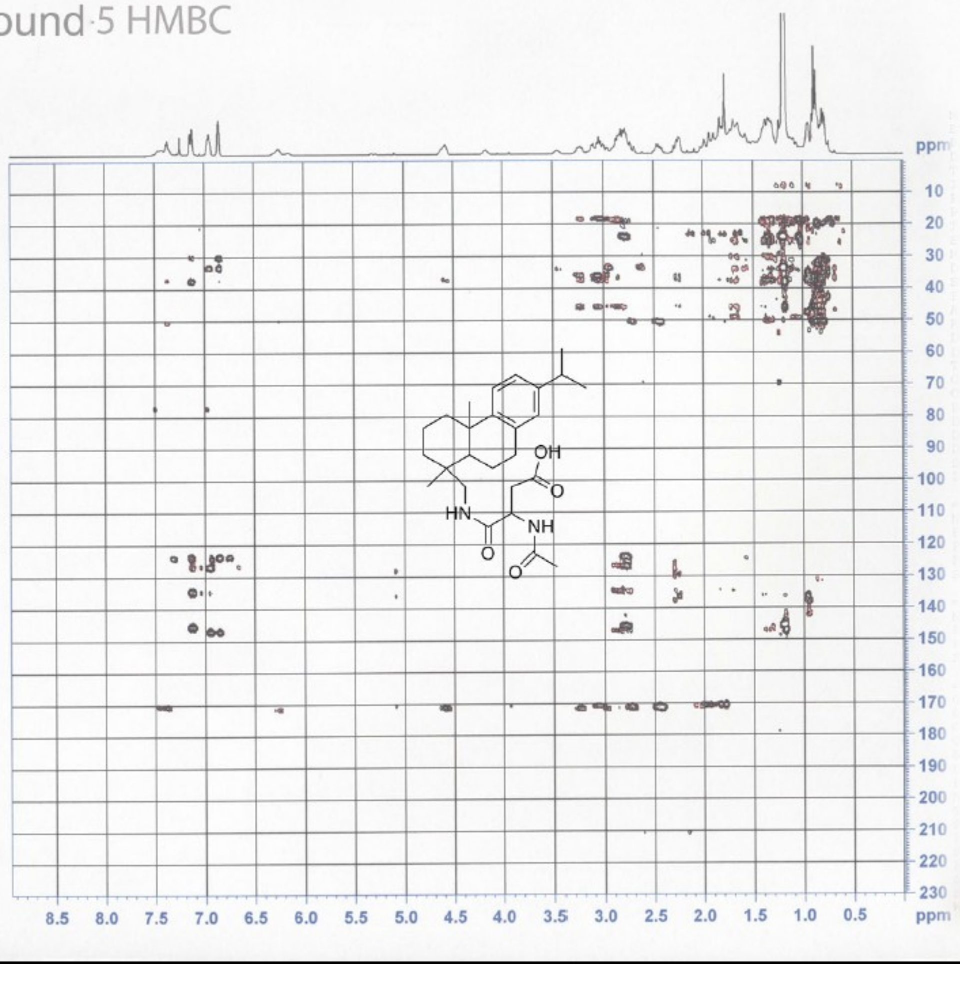

Compound 5 HSQC

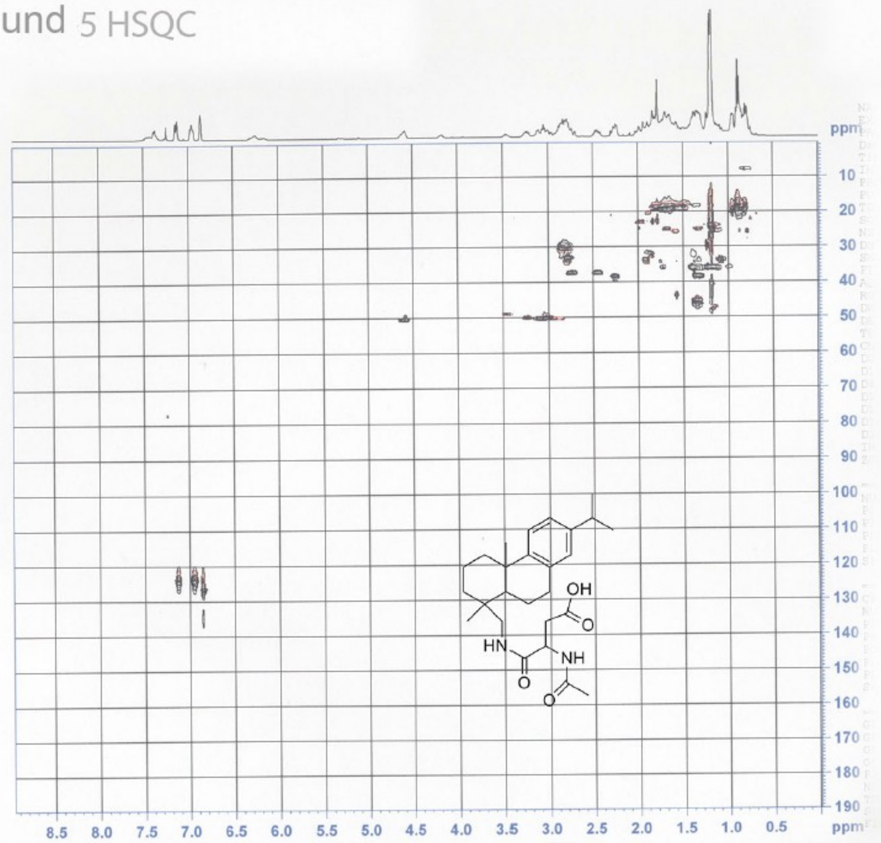

Compound 5 NOESY

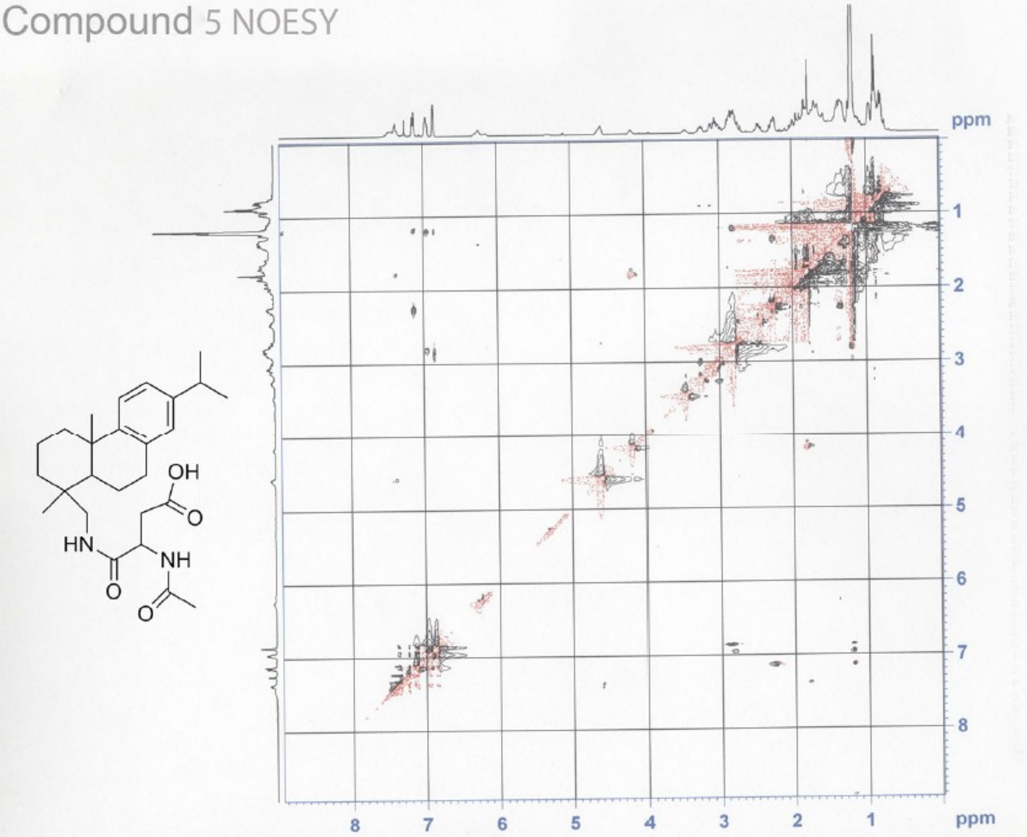

Compound 6 1H NMR

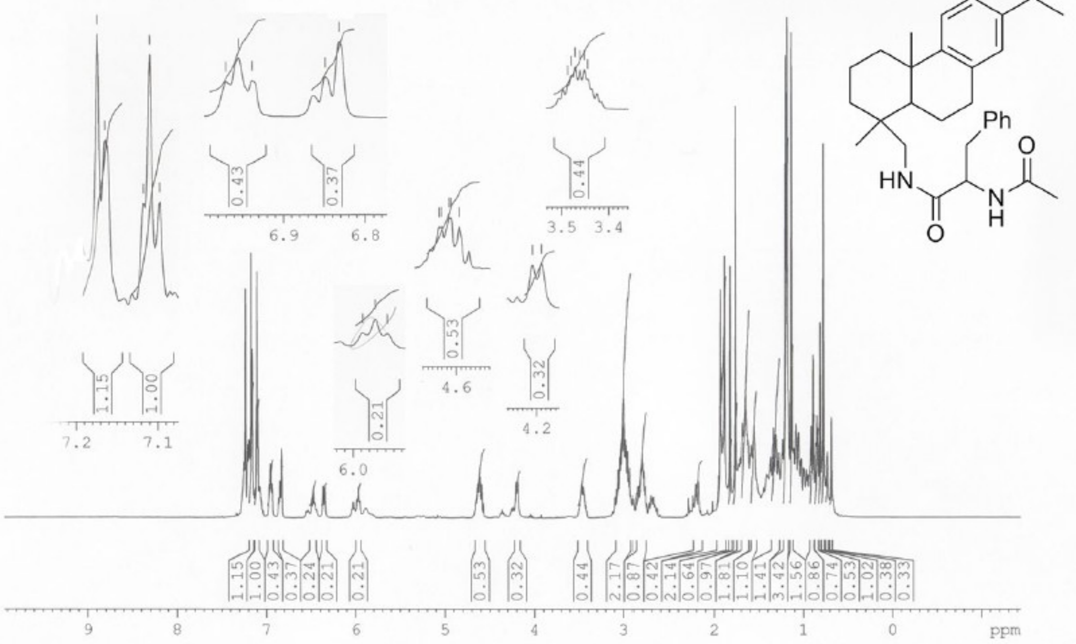

Compound 6 13C BB

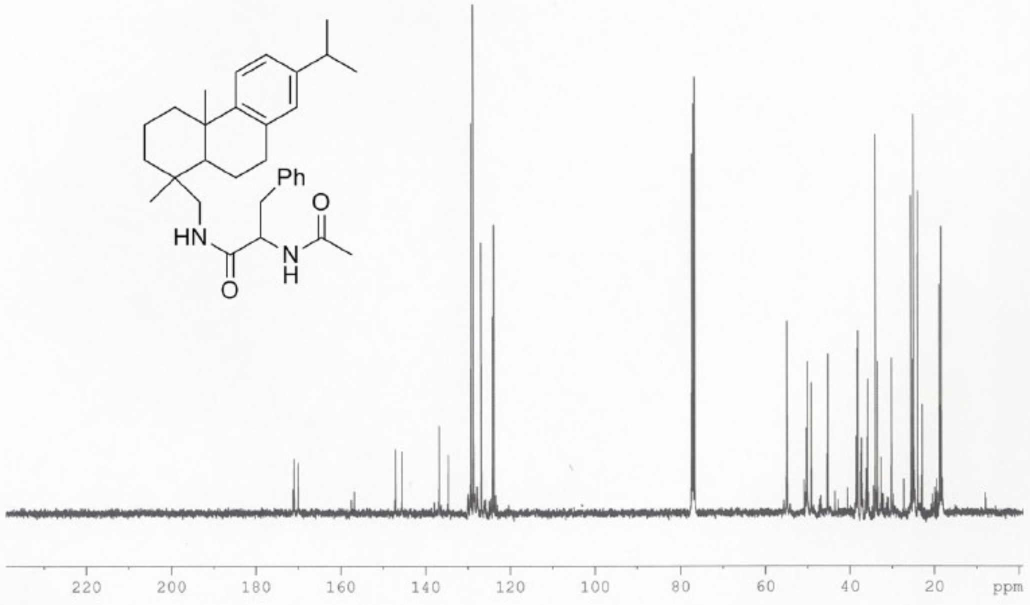

Compound 6 DEPT 90

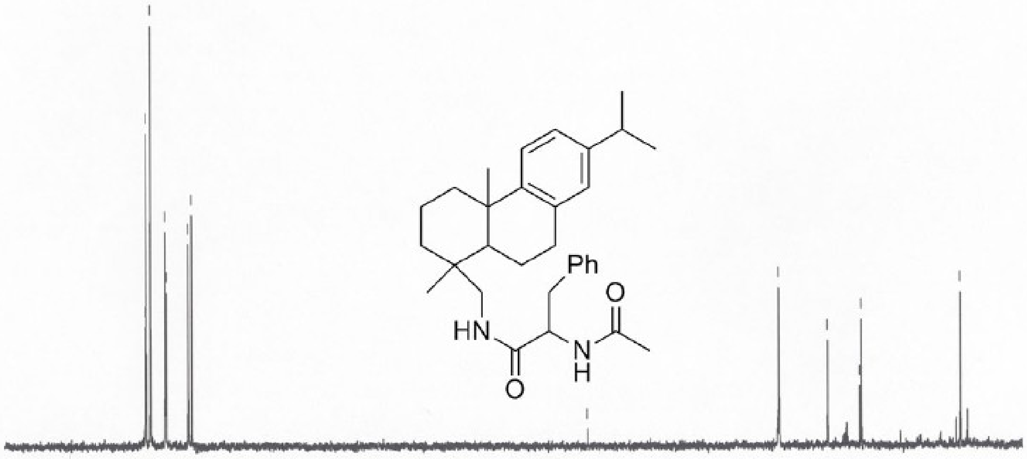

Compound 6 DEPT 135

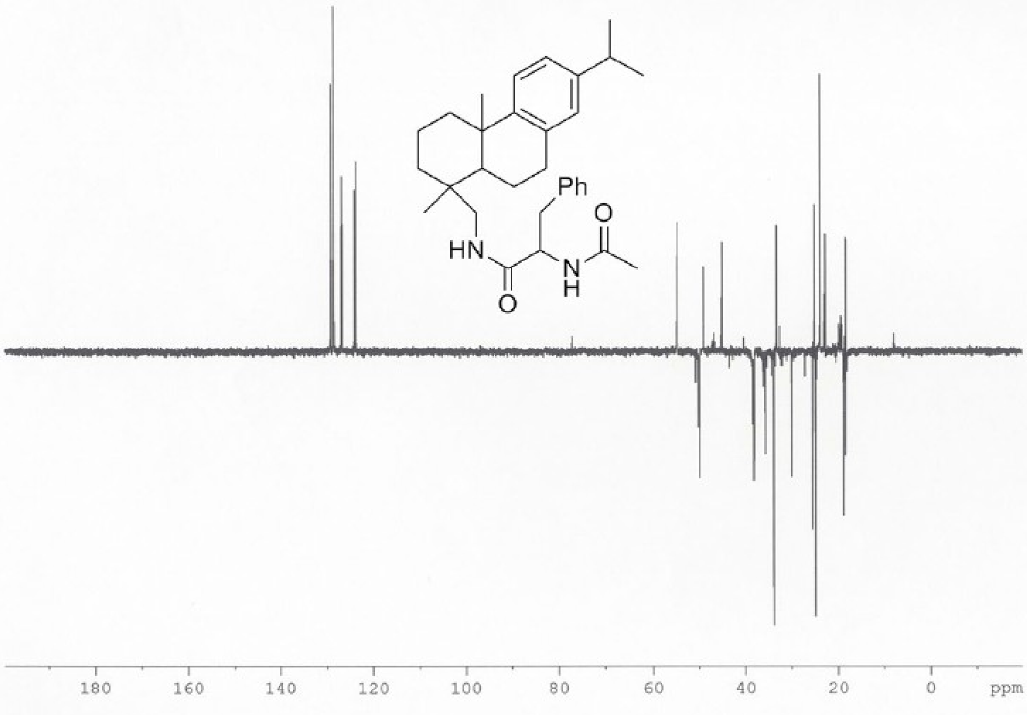

Compound -6 FTIR

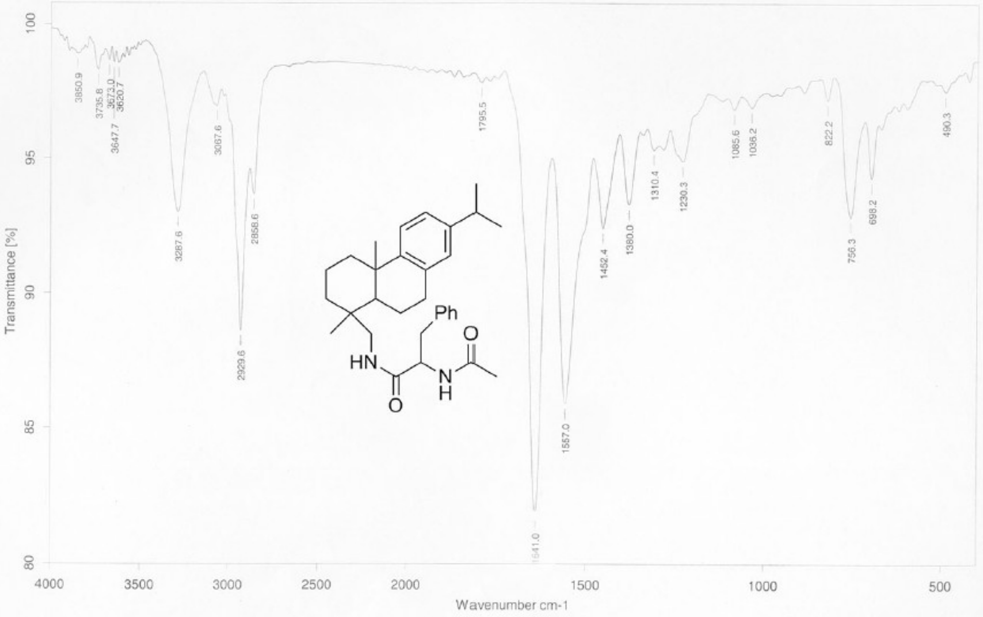

Compound-6 +TOF MS

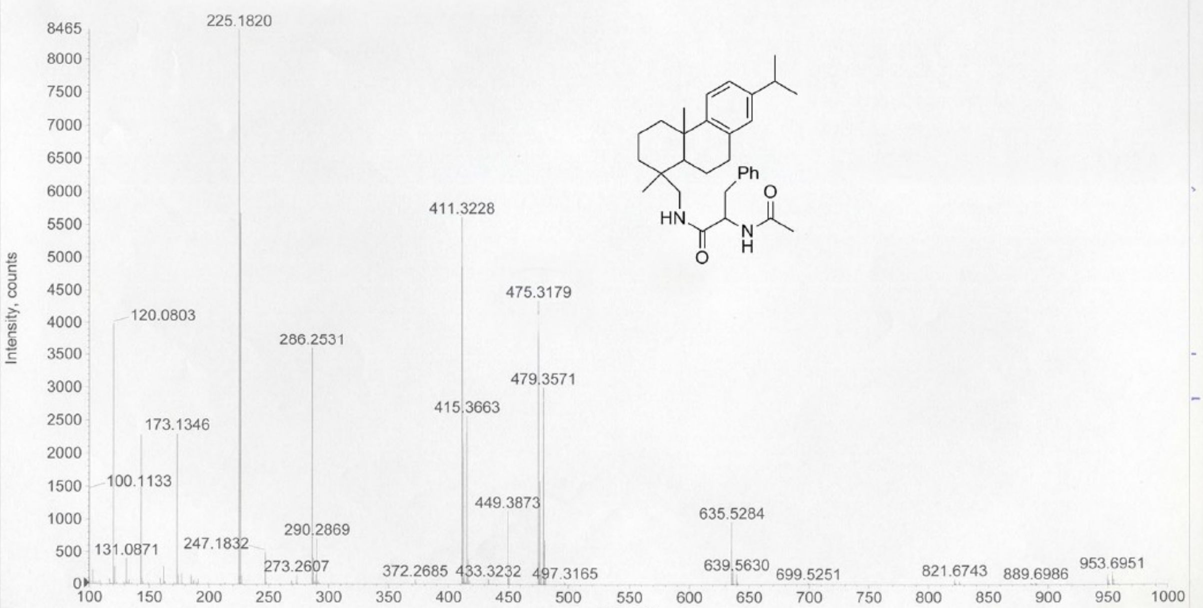

Compound 6 COSY

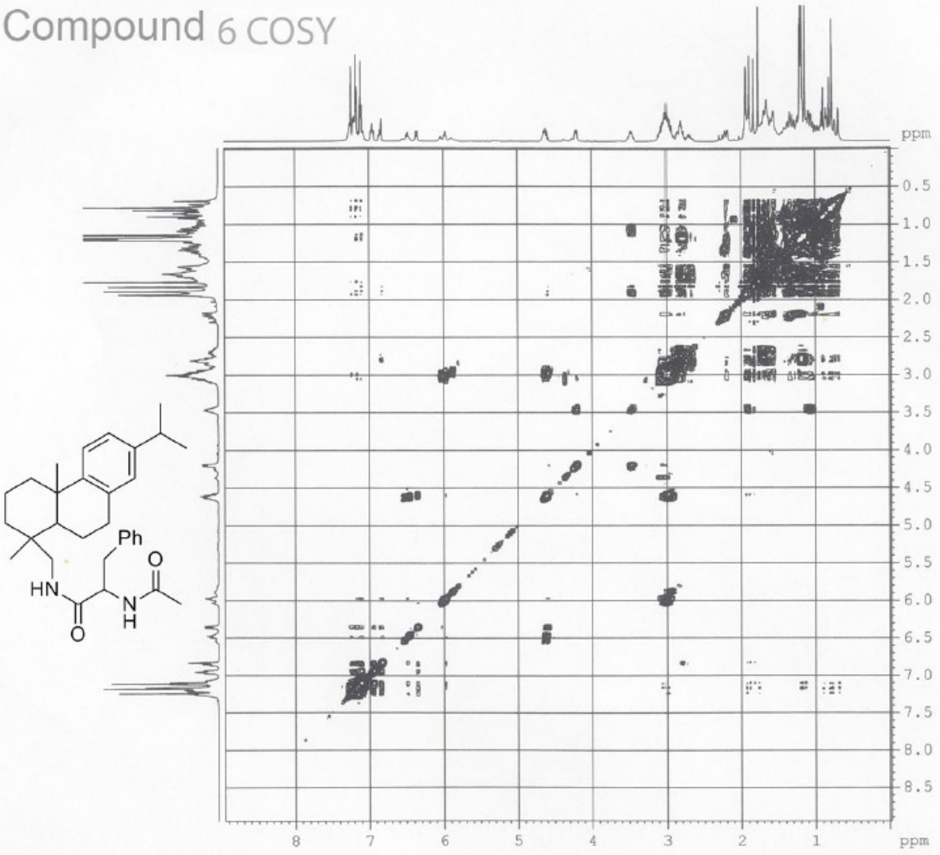

Compound 6 HMBC

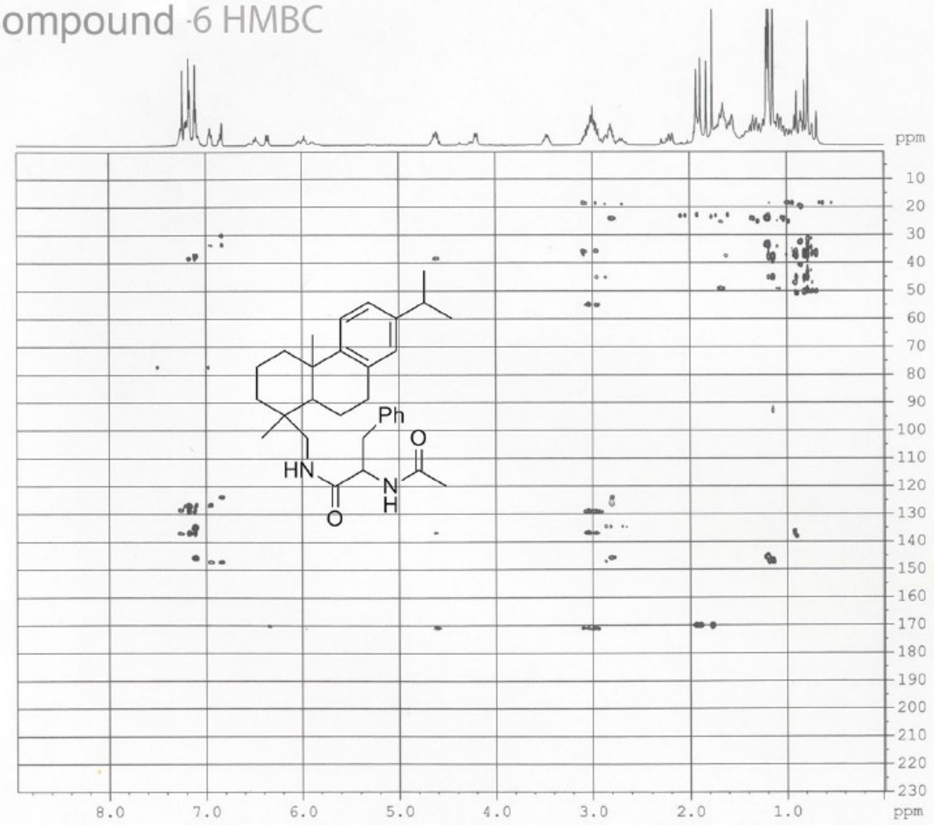

Compound 6 HSQC

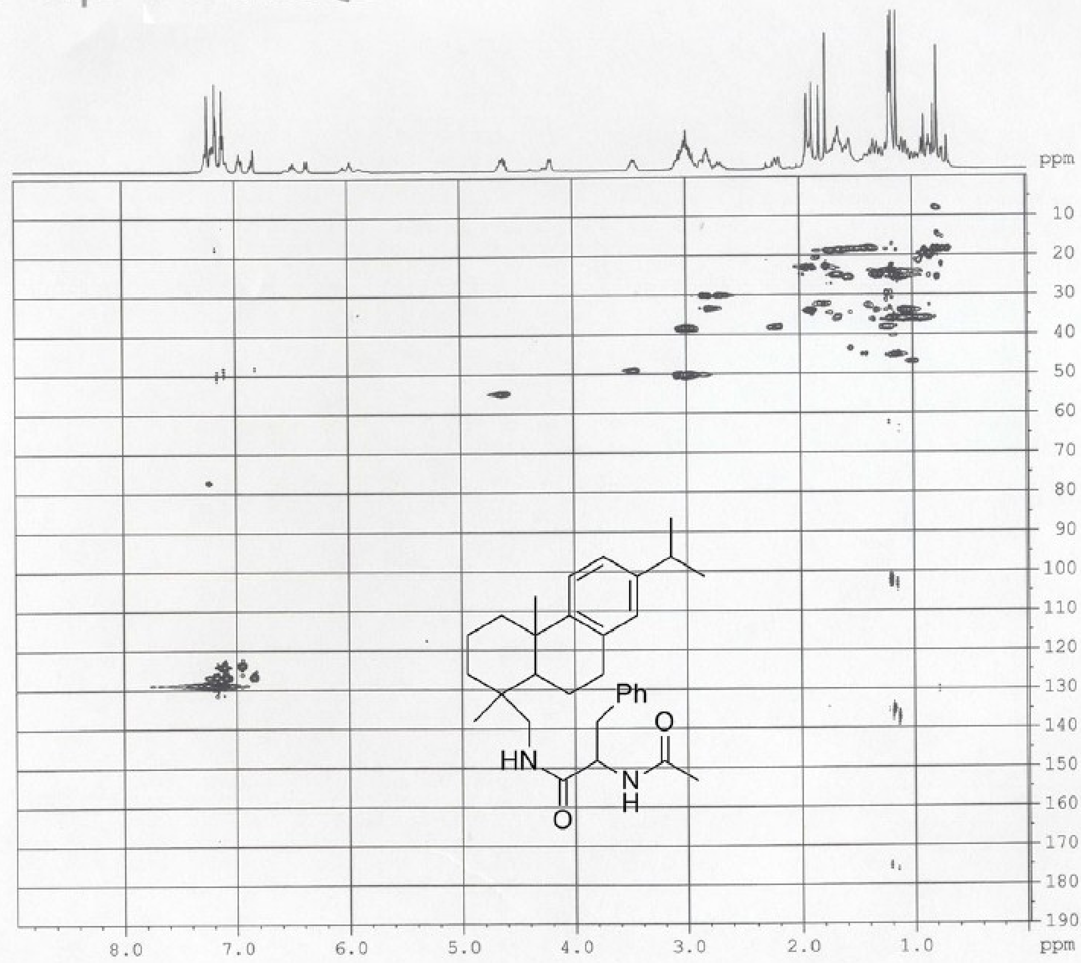

Compound 6 NOESY

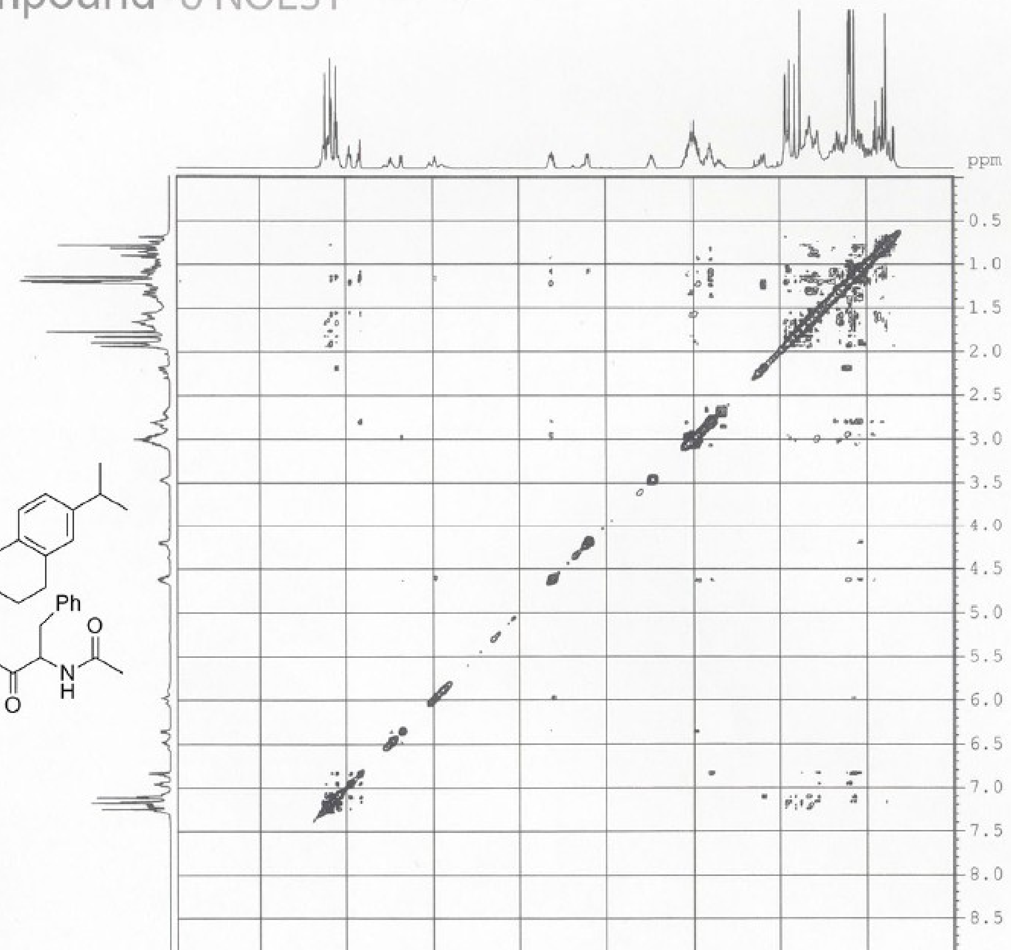

Compound 7 1H NMR

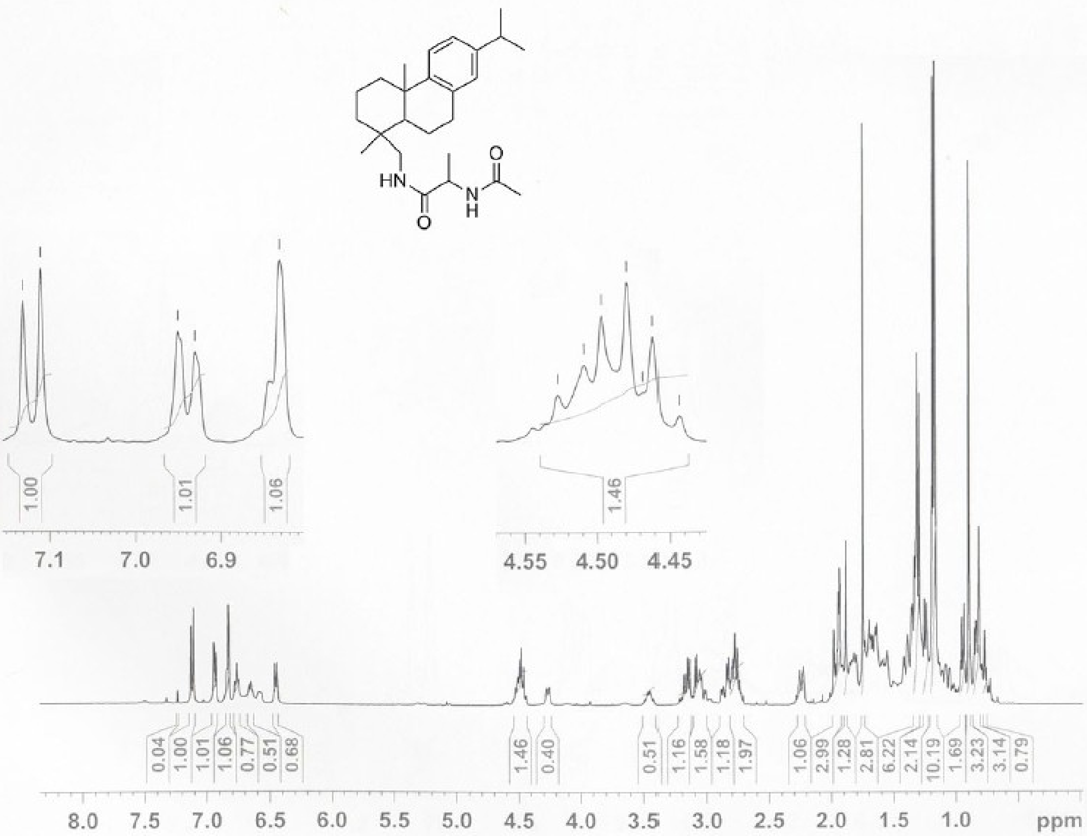

Compound 7 13C BB

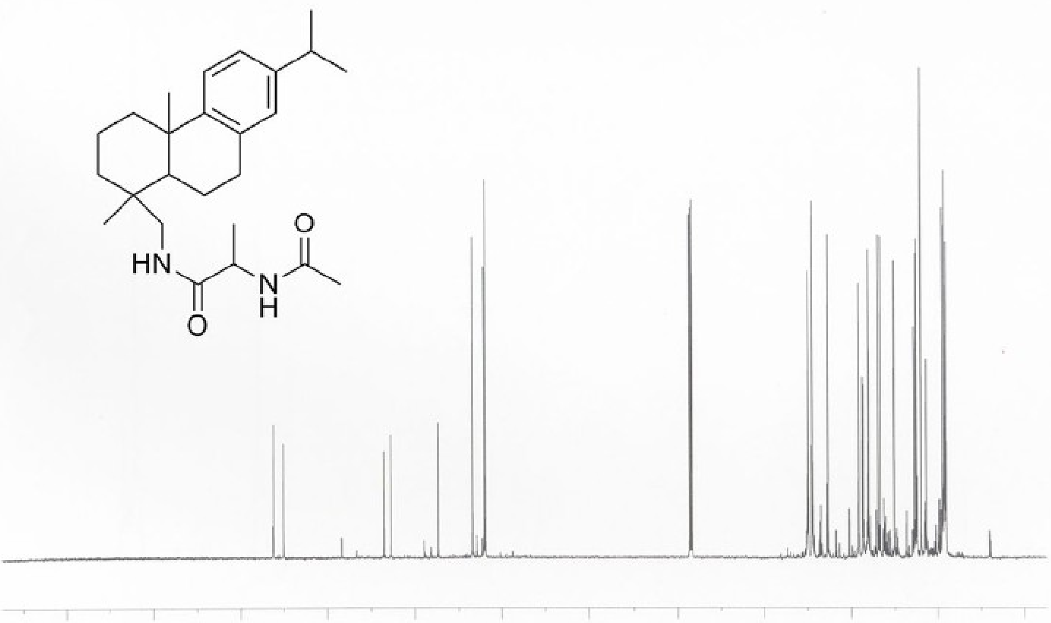

Compound 7 DEPT 90

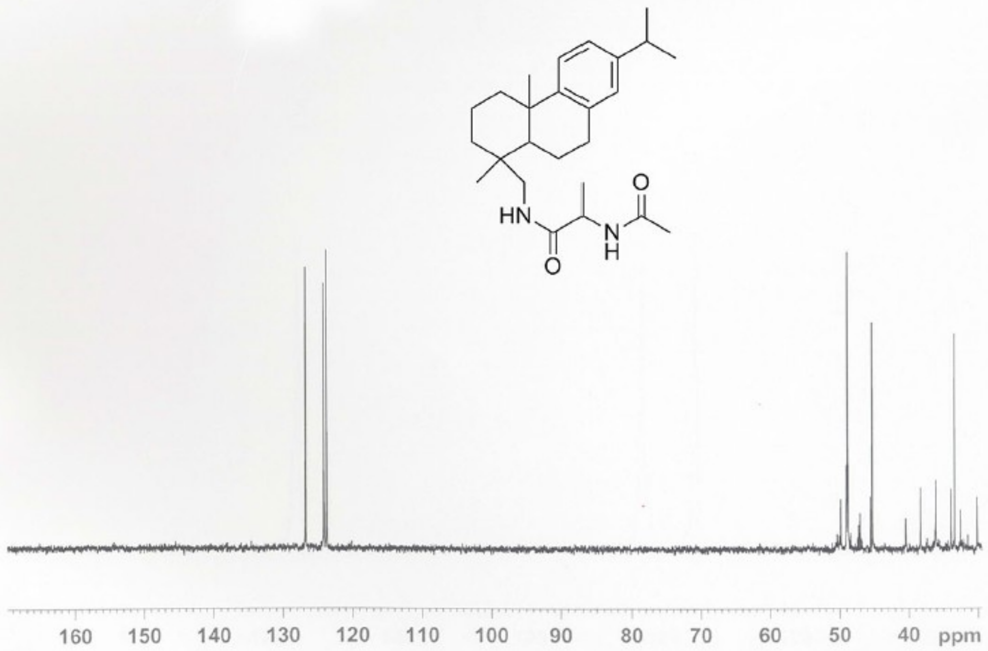

Compound 7 DEPT 135

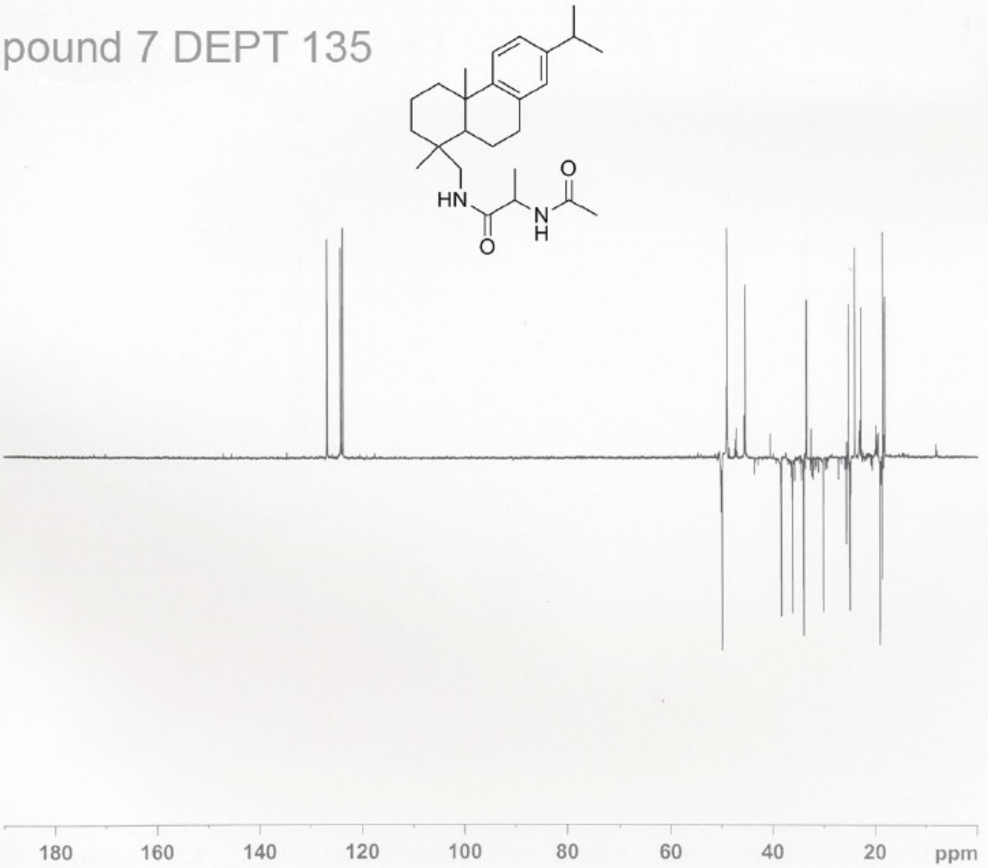

Compound 7 FTIR

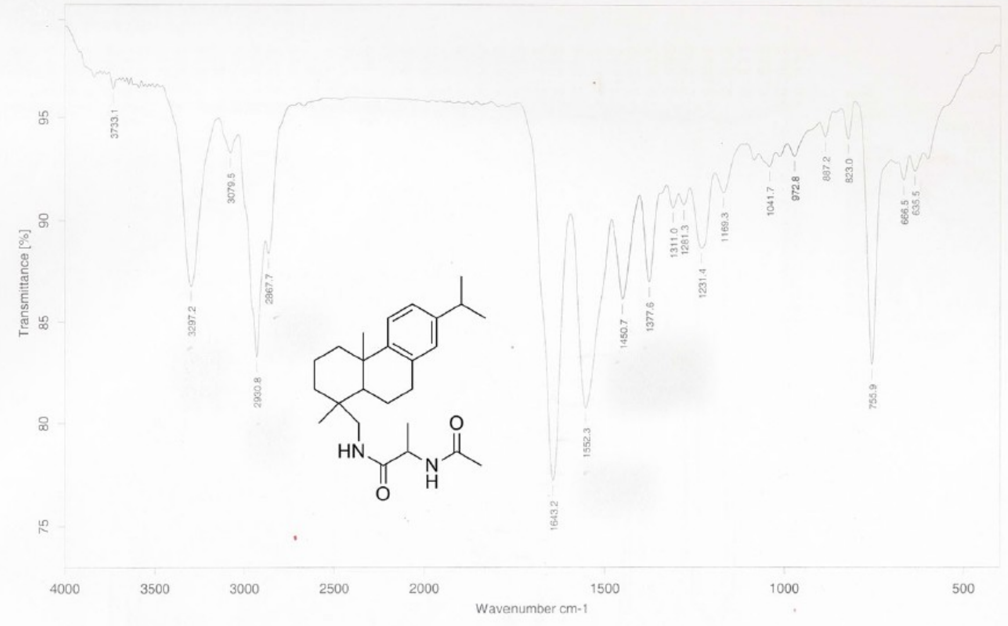

Compound 7 +TOF MS

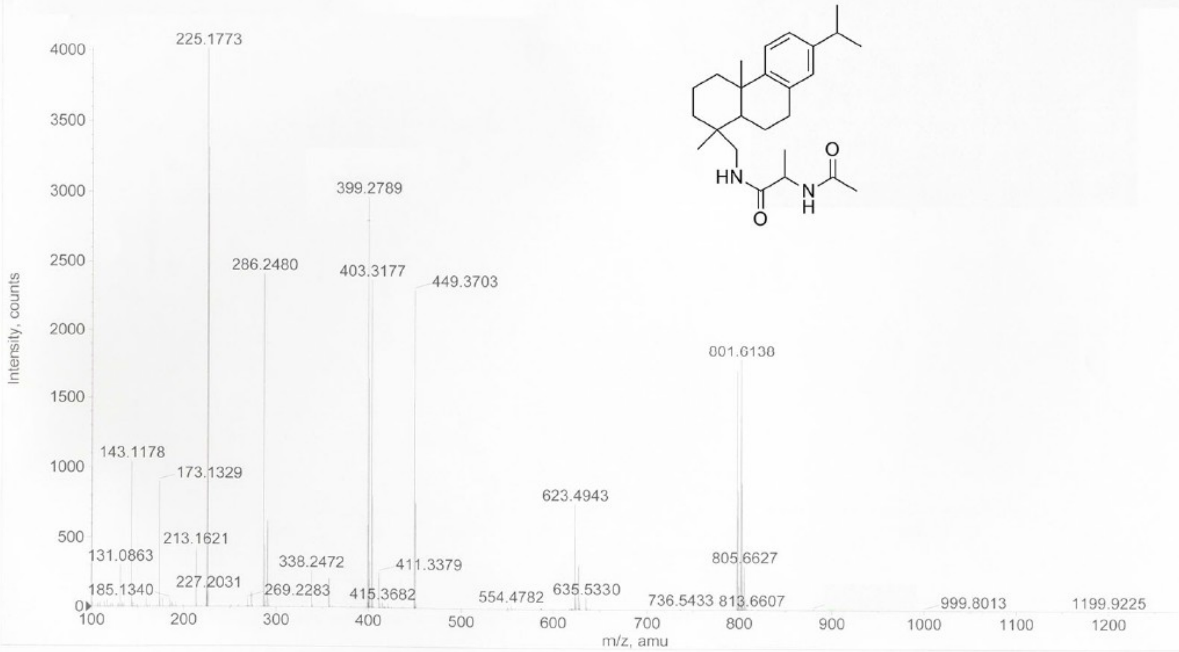

Compound 7 COSY

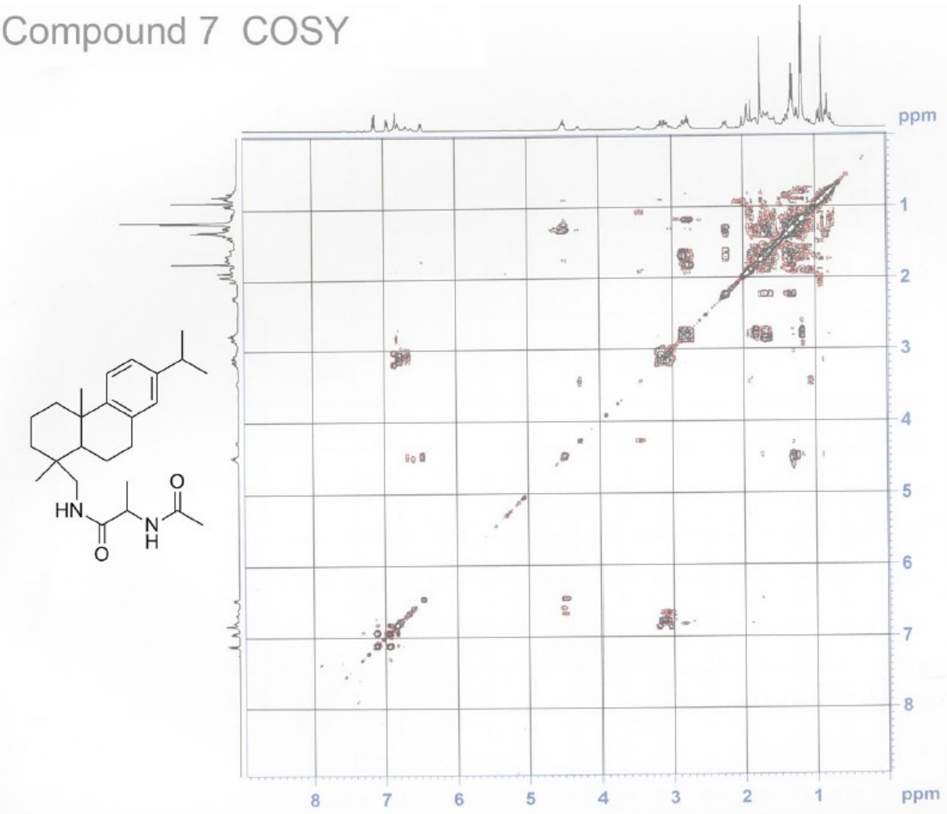

Compound 7 HMBC

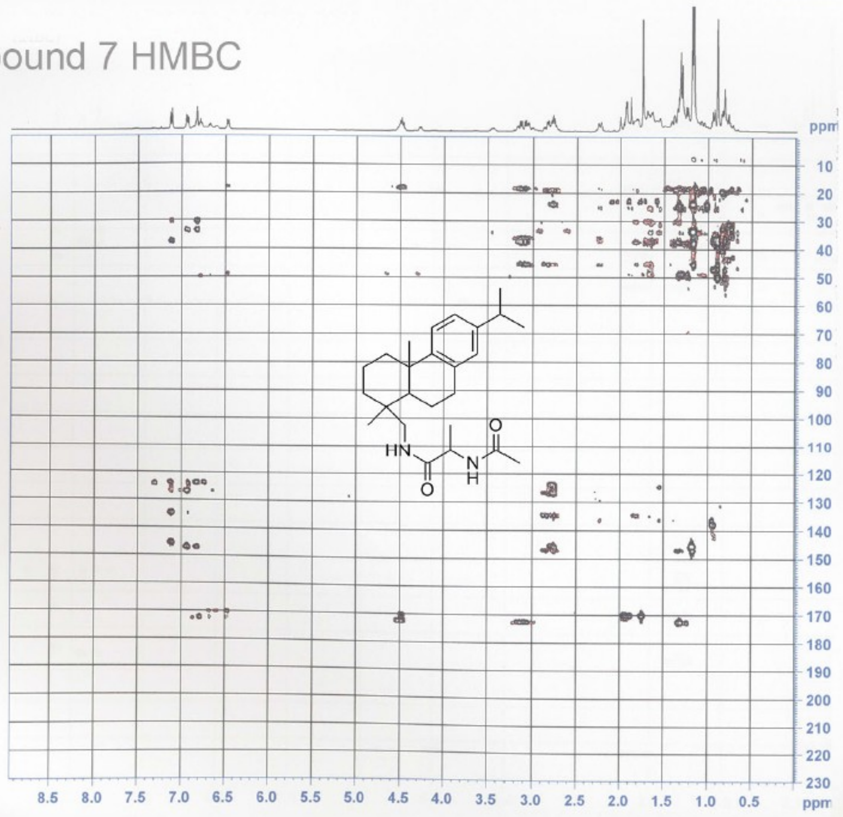

Compound 7 HSQC

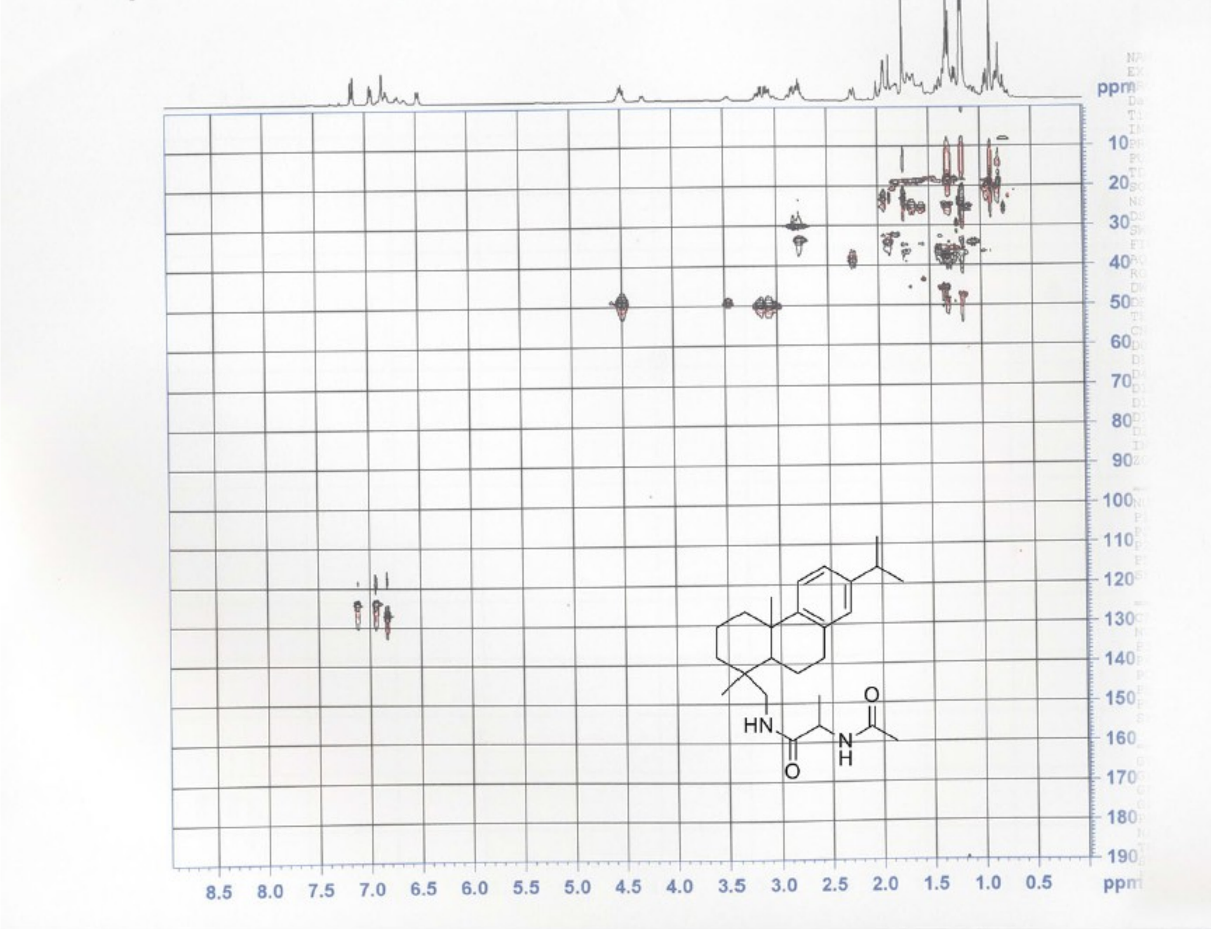

Compound 7 NOESY

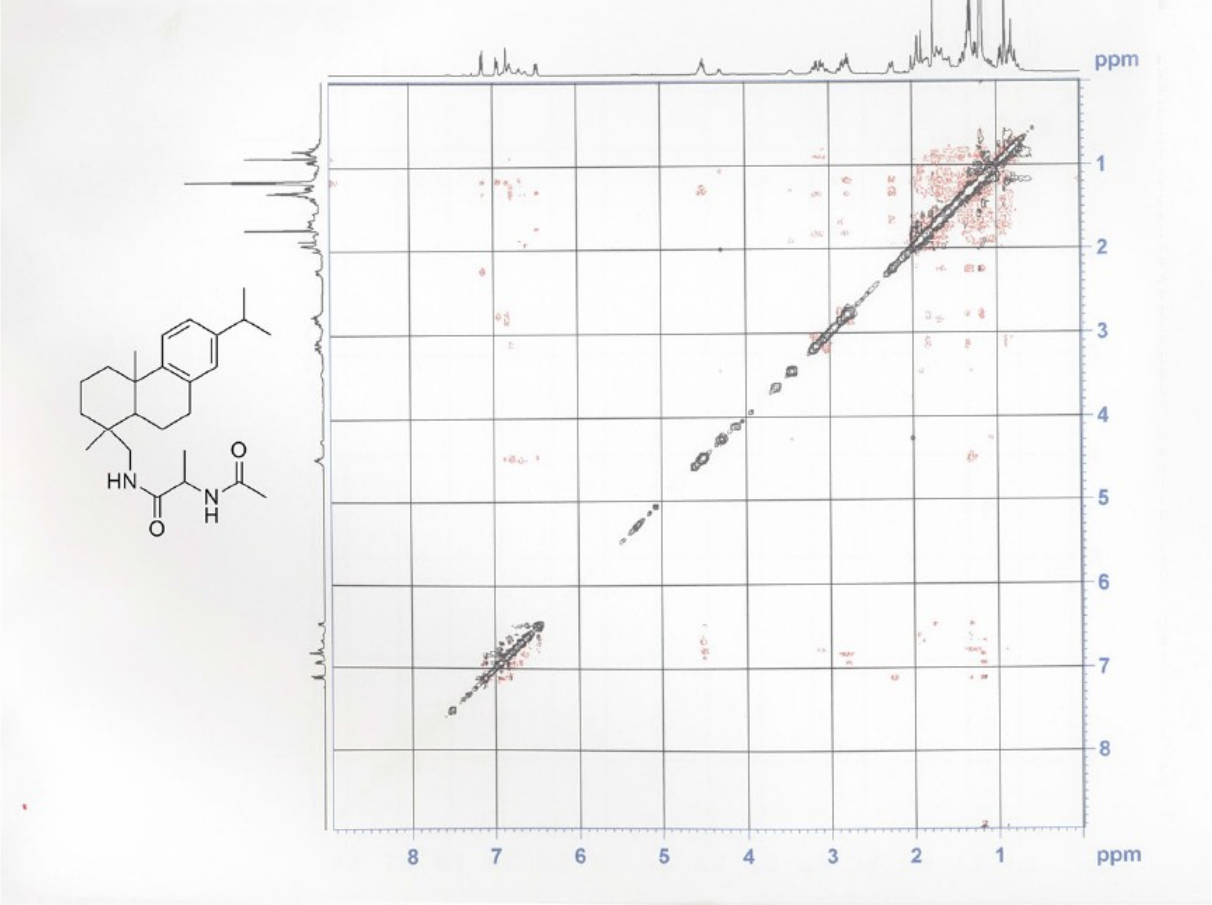

Supplement: Additional file 1: — Suppl. data Chemistry. Original spectra. Original spectra of ESI-MS, FTIR, 1H-NMR, 1D & 2D, 13C-NMR, COSY, HMBC, HSQC and NOESY spectroscopy of compounds 1–7. (PDF 5506 kb) [file 12885_2016_2942_MOESM1_ESM.pdf]
