# Supplementary material for: Synthesis and bio-molecular study of (+)-N-Acetyl-α-amino acid dehydroabietylamine derivative for the selective therapy of hepatocellular carcinoma
Source: BMC Cancer. 2016 Nov 14;16:883. doi: 10.1186/s12885-016-2942-5 (PMC5109647; doi:10.1186/s12885-016-2942-5)
Supplement: Additional file 2: — Characterization and Synthesis. Spectroscopic data. Characterization of compounds through spectral data. (PDF 366 kb) [file 12885_2016_2942_MOESM2_ESM.pdf]

Synthesis of 2-acetamido-N-((1,2,3,4,4a,9,10,10a-octahydro-7-isopropyl-1,4a-dimethylphenanthren-1-yl)methyl)ethanamide (DAAD1)

Yield: 82.85%; **$[\alpha]_D^{26}$:** 13.73 (C = 0.0288, $CHCl_3$); **ESI –MS:** $[M + H]^+$ at *m/z* 385 and $[2M + H]^+$ at *m/z* 769; **IR (cm^{-1}):** 3301 (N–H stretching), 2929 (C-H), 2856 (C-H), 1648 (C=O), 1554 (N–H bending), 1450 (CH_2 bending), 1381 (CH_3 bending); **^{1}HNMR (400 MHz, $CDCl_3$) δ ppm:** 0.898 (3H, s, H-19), 1.174 (3H, s, H-20), 1.192 (6H, d, *J* = 7.2 Hz, H-16 & 17), 1.399 (5H, m, H-1,3 & 5), 1.878 (5H, s, H-2 & 5'), 2.782 (1H, m, H-15), 2.884 (2H, m, H-7), 3.050 (1H, dd, *J* = 6.0 & 13.6 Hz, H-18a), 3.253 (1H, dd, *J* = 6.4 &13.2 Hz, H-18b), 3.875 (2H, d, *J* = 5.2 Hz, H-2'), 6.840 (1H, s, H-14), 6.956 (1H, d, *J* = 8.0 Hz, H-12), 7.134 (1H, d, *J* = 8.0 Hz, H-11); **^{13}CNMR (400 MHz, $CDCl_3$) δ ppm:** 18.51 (C-19), 18.91 (C-2 & 6), 22.53 (C-5'), 23.85 (C-16), 23.89 (C-17), 25.16 (C-20), 30.06 (C-7), 33.33 (C-15), 36.01 (C-3), 37.30 (C-4), 37.38 (C-10), 38.20 (C-1), 43.71 (C-2'), 45.12 (C-5), 49.92 (C-18), 123.72 (C-12), 124.06 (C-11), 126.75 (C-14), 134.59 (C-8), 145.48 (C-13), 147.04 (C-9), 169.33 (C-4'), 170.93 (C-1').

Synthesis of 2-acetamido-N-((1,2,3,4,4a,9,10,10a-octahydro-7-isopropyl-1,4a-dimethylphenanthren-1-yl)methyl)-3-mercaptopropanamide (DAAD2)

Yield: 82.01%; **$[\alpha]_D^{26}$:** 21.73 (C = 0.0342, $CHCl_3$); **ESI –MS:** $[M + H]^+$ at *m/z* 431; **IR (cm^{-1}):** 3294 (N–H stretching), 2929 (C-H), 2856 (C-H), 2550 (S-H), 1645 (C=O), 1541 (N–H bending), 1453 (CH_2 bending), 1379 (CH_3 bending); **^{1}HNMR (400 MHz, $CDCl_3$) δ ppm:** 0.910 (3H, s, H-19), 1.172 (6H, s, H-20 & H-5'), 1.187 (6H, d, *J* = 6.0 Hz, H-16 & 17), 1.370 (5H, m, H-1,3 & 5), 1.717 (4H, m, H-2 & 6), 2.833 (5H, m, H-7,15 & 6'),), 3.050 (1H, dd, *J* = 6.0 & 13.6 Hz, H-18a), 3.253 (1H, dd, *J* = 6.4 & 13.2 Hz, H-18b), 5.081 (1H, t, *J* = 6.8 Hz, H-2'), 6.827 (1H, s, H-14), 6.950 (1H, d, *J* = 8.0 Hz, H-12), 7.131 (1H, d, *J* = 8.0 Hz, H-11); **^{13}CNMR (400 MHz, $CDCl_3$) δ ppm:** 18.34 (C-19), 18.76 (C-2), 19.08 (C-6), 22.96 (C-5'), 24.00 (C-16 & 17), 25.32 (C-20), 29.7 (C-6'), 30.23 (C-7), 33.44 (C-15), 36.20 (C-3), 37.31

(C-4), 37.53 (C-10), 38.30 (C-1), 49.10 (C-5), 50.68 (C-18), 54.19 (C-2'), 123.83 (C-12), 124.20 (C-11), 126.81 (C-14), 134.75 (C-8), 145.60 (C-13), 147.15 (C-9), 170.23 (C - 4'), 170.70 (C-1').

Synthesis of 2-acetamido-N-((1,2,3,4,4a,9,10,10a-octahydro-7-isopropyl-1,4a-dimethylphenanthren-1-yl)methyl)-4-(methylthio)butanamide (DAAD3)

Yield: 79.65%; **$[\alpha]_D^{26}$:** -20.94 (C = 0.0042, $CHCl_3$); **ESI –MS:** $[M + H]^+$ at *m/z* 459

IR (cm^{-1}): 3292 (N–H stretching), 2928 (C-H), 2853 (C-H), 1642 (C=O), 1555 (N–H bending), 1447 (CH_2 bending), 1379 (CH_3 bending); **^{1}HNMR (400 MHz, $CDCl_3$) δ ppm:** 0.906 (3H, s, H-19), 1.189 (6H, d, J = 6.0 Hz, H-16 & 17), 1.195 (3H, s, H-20), 1.232 (2H, m, H-7), 1.351 (5H, m, H-1,3 & 5),1.678 (2H, m, H-2), 1.746 (3H, s, H-5'), 1.889 (2H, m, H-6), 1.916 (2H, m, H-6'), 2.018 (3H, s, H-8'), 2.558 (2H, t, J = 6.0 Hz, H-7'), 2.783 (1H, m, H-15), 2.864 (2H, m, H-7), 3.067 (1H, dd, J = 6.0 & 13.6 Hz, H-18a), 3.252 (1H, dd, J = 6.8 & 13.6 Hz, H-18b), 4.524 (1H, t, J = 7.2 Hz, H-2'), 6.841 (1H, s, H-14), 6.954 (1H, d, J = 8.0 Hz, H-12), 7.135 (1H, d, J = 8.0 Hz, H-11); **^{13}CNMR (400 MHz, $CDCl_3$) δ ppm:** 15.17 (C-8'), 18.56 (C-2), 18.62 (C-19), 19.03 (C-6), 22.77 (C-5'), 23.95 (C-16 & 17), 25.61 (C-20), 30.16 (C-7'), 30.30 (C-7), 30.91 (C-6'), 33.43 (C-15), 36.17 (C-3), 37.35 (C-4), 37.44 (C-10), 38.35 (C-1), 45.28 (C-5), 49.95 (C-18), 52.36 (C-2'), 123.80 (C-12), 124.19 (C-11), 126.77 (C-14), 134.60 (C-8), 145.64 (C-13), 147.13 (C-9), 170.23 (C - 4'), 171.01 (C-1').

Synthesis of 2-acetamido-N-((1,2,3,4,4a,9,10,10a-octahydro-7-isopropyl-1,4a-dimethylphenanthren-1-yl)methyl)-3-(4-hydroxyphenyl)propanamide (DAAD4)

Yield: 77.94%; **$[\alpha]_D^{26}$:** 7.53 (C = 0.019, $CHCl_3$); **ESI –MS:** $[M + H]^+$ at *m/z* 491 and $[2M + H]^+$ at *m/z* 981; **IR (cm^{-1}):** 3293 (N–H stretching), 2930 (C-H), 2857 (C-H), 1643 (C=O), 1552 (N–H bending), 1449 (CH_2 bending), 1377 (CH_3 bending); **^{1}HNMR (400 MHz, $CDCl_3$) δ ppm:** 0.799 (3H, s, H-19), 1.136 (3H, s, H-20), 1.191 (6H, d, J = 6.8 Hz, H-16 & 17), 1.212 (5H, m, H-1,3 & 5), 1.668 (4H, m, H-2 & 6), 1.762 (3H, s, H-5'), 2.831 (3H, m, H-7 &

15), 2.912 (2H, d, J = 6.2 Hz, H-6'), 3.022 (1H, dd, J = 6.4 & 13.6 Hz, H-18a), 3.104 (1H, dd, J = 6.4 & 13.6 Hz, H-18b), 4.606 (1H, t, J = 6.0 Hz, H-2'), 6.632 (2H, d, J = 8.4 Hz, H-9' & 11'), 6.839 (1H, s, H-14), 6.941 (1H, d, J = 8.0 Hz, H-12), 6.990 (2H, d, J = 8.4 Hz, H-8' & 12'), 7.126 (1H, d, J = 8.0 Hz, H-11); **^{13}CNMR (400 MHz, $CDCl_3$) δ ppm:** 18.38 (C-19), 18.48 (C-6), 18.92 (C-2), 22.77 (C-5'), 23.97 (C-16 & 17), 25.16 (C-20), 30.10 (C-7), 33.41 (C-15), 35.82 (C-3), 37.25 (C-4), 37.32 (C-10), 37.45 (C-6'), 38.13 (C-1), 45.21 (C-5), 50.07 (C-18), 55.08 (C-2'), 115.71 (C-9' & 11'), 123.77 (C-12), 124.21 (C-11), 126.76 (C-14), 127.70 (C-7'), 130.16 (C-8' & 12'), 134.68 (C-8), 145.62 (C-13), 147.14 (C-9), 155.46 (C-10'), 170.49 (C - 4'), 171.32 (C-1').

Synthesis of 3-((1,2,3,4,4a,9,10,10a-octahydro-7-isopropyl-1,4a-dimethylphenanthren-1-yl)methylcarbamoyl)-3-acetamidopropanoic acid (DAAD5)

Yield: 78.66%; **$[\alpha]_D^{26}$:** 8.03 (C = 0.0183, $CHCl_3$); **ESI –MS:** $[M + H]^+$ at *m/z* 443

IR (cm^{-1}): 3306 (N–H stretching), 2929 (C-H), 2863 (C-H), 1645 (C=O), 1551 (N–H bending), 1451 (CH_2 bending), 1378 (CH_3 bending); **^{1}HNMR (400 MHz, $CDCl_3$) δ ppm:** 0.887 (3H, s, H-19), 1.177 (3H, s, H-20), 1.999 (6H, d, J = 6.8 Hz, H-16 & 17), 1.350 (5H, m, H-1,3 & 5), 1.778 (3H, s, H-5'), 2.293 (2H, m, H-1), 2.734 (2H, d, J = 6.2 Hz, H-6'), 2.783 (1H, m, H-15), 2.828 (2H, m, H-7), 3.064 (1H, dd, J= 6.4 & 13.2 Hz, H-18a), 3.259 (1H, dd, J = 7.2 & 13.6 Hz, H-18b), 4.596 (1H, t, J = 6.4 Hz, H-2'), 6.851 (1H, s, H-14), 6.961 (1H, d, J = 8.0 Hz, H-12), 7.137 (1H, d, J = 8.0 Hz, H-11); **^{13}CNMR (400 MHz, $CDCl_3$) δ ppm:** 18.42 (C-19), 18.58 (C-6), 19.02 (C-2), 22.79 (C-5'), 23.95 (C-16 & 17), 25.22 (C-20), 30.16 (C-7), 33.39 (C-15), 36.12 (C-3), 37.31 (C-6'), 37.40 (C-4), 37.44 (C-10), 38.34 (C-1), 45.53 (C-5), 50.39 (C-2'), 50.48 (C-18), 123.75 (C-12), 124.11 (C-11), 126.79 (C-14), 134.63 (C-8), 145.51 (C-13), 147.08 (C-9), 170.67 (C - 4'), 170.92 (C-1'), 171.67 (C-7').

Synthesis of 2-acetamido-N-((1,2,3,4,4a,9,10,10a-octahydro-7-isopropyl-1,4a-dimethylphenanthren-1-yl)methyl)-3-phenylpropanamide (DAAD6)

Yield: 76.18%; **[α]$_D^{26}$:** 5.34 (C = 0.0189, $CHCl_3$); **ESI –MS:** $[M + H]^+$ at *m/z* 475

IR (cm^{-1}): 3287 (N–H stretching), 2929 (C-H), 2856 (C-H), 1641 (C=O), 1557 (N–H bending), 1452 (CH_2 bending), 1380 (CH_3 bending); **^{1}HNMR (400 MHz, $CDCl_3$) *δ* ppm:** 0.778 (3H, s, H-19), 1.138 (3H, s, H-20), 1.208 (6H, d, *J* = 6.8 Hz, H-16 & 17), 1.3414 (2H, m, H-1), 1.559 (2H, m, H-6), 1.654 (2H, m, H-2), 1.766 (3H, s, H-5'), 2.819 (3H, m, H-7 & 15), 2.970 (1H, dd, *J* = 6.8 & 14.0 Hz, H-18a), 3.062 (1H, dd, *J* = 6.4 & 13.6 Hz, H-18b), 4.636 (1H, t, *J* = 6.8 Hz, H-2'), 6.832 (1H, s, H-14), 6.955 (1H, d, *J* = 8.0 Hz, H-12), 7.117 (4H, d, *J* = 8.4 Hz, H-11, 8', 10' & 12'), 7.174 (2H, d, *J* = 8.4 Hz, H-9' & 11'); **^{13}CNMR (400 MHz, $CDCl_3$) *δ* ppm:** 18.50 (C-6), 18.52 (C-19), 18.93 (C-2), 22.86 (C-5'), 23.99 (C-16 & 17), 25.19 (C-20), 30.13 (C-7), 33.47 (C-15), 35.81 (C-3), 37.21 (C-4), 37.33 (C-10), 38.18 (C-6'), 38.29 (C-1), 45.15 (C-5), 50.01 (C-18), 54.88 (C-2'), 123.80 (C-12), 124.19 (C-11), 126.78 (C-10'), 126.91 (C-14), 128.65 (C-8' & 12'), 129.15 (C-9' & 11'), 134.63 (C-8), 136.77 (C-7'), 145.63 (C-13), 147.13 (C-9), 170.01 (C - 4'), 171.04 (C-1').

Synthesis of 2-acetamido-N-((1,2,3,4,4a,9,10,10a-octahydro-7-isopropyl-1,4a-dimethylphenanthren-1-yl)methyl)propanamide (DAAD7)

Yield: 82.63%; **[α]$_D^{26}$:** -28.70(C = 0.0154, $CHCl_3$); **ESI –MS:** $[M + H]^+$ at *m/z* 399

IR (cm^{-1}): 3297 (N–H stretching), 2929 (C-H), 2867 (C-H), 1643 (C=O), 1552 (N–H bending), 1450 (CH_2 bending), 1377 (CH_3 bending); **^{1}HNMR (400 MHz, $CDCl_3$) *δ* ppm:** 0.898 (3H, s, H-19), 1.187 (9H, d, *J* = 6.8 Hz, H-16,17 & 20), 1.254 (2H, m, H-3) , 1.316 (3H, d, *J* = 6.8 Hz, H-6'), 1.36 (3H, m, H-1 & 5), 1.67 (4H, m, H-2 & 6), 1.748 (3H, s, H-5'), 2.795 (1H, m, H-15), 2.888 (2H, m, H-7), 3.099 (1H, dd, *J* = 6.4 & 13.6 Hz, H-18a), 3.190 (1H, dd, *J* = 6.8 & 14.0 Hz, H-18b), 4.479 (1H, m, H-2'), 6.830 (1H, s, H-14), 6.950 (1H, d, *J* = 8.0 Hz, H-12), 7.130 (1H, d, *J* = 8.0 Hz, H-11); **^{13}CNMR (400 MHz, $CDCl_3$) *δ* ppm:** 18.09 (C-6'), 18.48 (C-19), 18.56 (C-6), 18.99 (C-2), 22.71 (C-5'), 23.93 (C-16 & 17), 25.18 (C-20), 30.15 (C-7), 33.39 (C-15), 36.09 (C-3), 37.35 (C-4), 37.50 (C-10), 38.29 (C-1), 45.31 (C-5),

48.87 (C-2'), 49.88 (C-18), 123.75 (C-12), 124.16 (C-11), 126.76 (C-14), 134.64 (C-8), 145.55 (C-13), 147.14 (C-9), 170.13 (C - 4'), 172.44 (C-1').
