# Supplementary figures and images for: Synthesis and bio-molecular study of (+)-N-Acetyl-α-amino acid dehydroabietylamine derivative for the selective therapy of hepatocellular carcinoma
Source: BMC Cancer. 2016 Nov 14;16:883. doi: 10.1186/s12885-016-2942-5 (PMC5109647; doi:10.1186/s12885-016-2942-5)

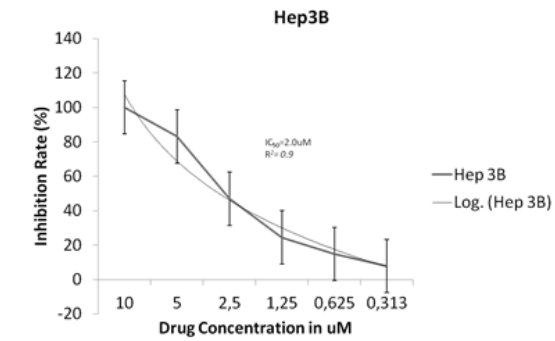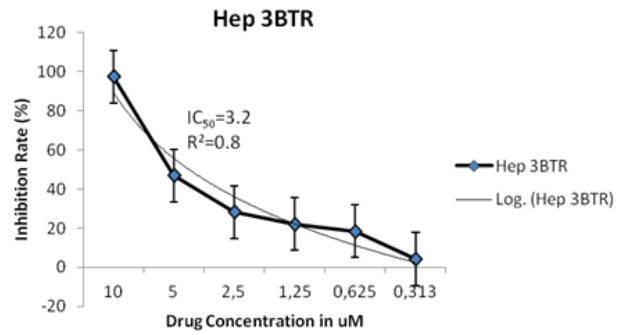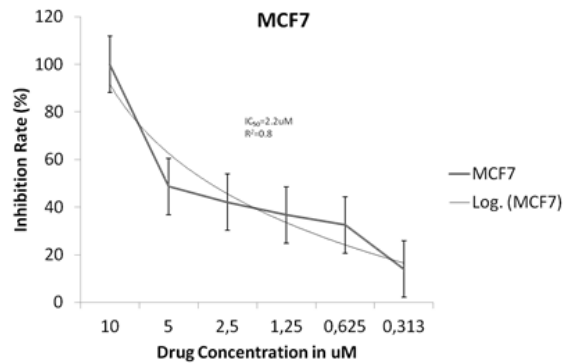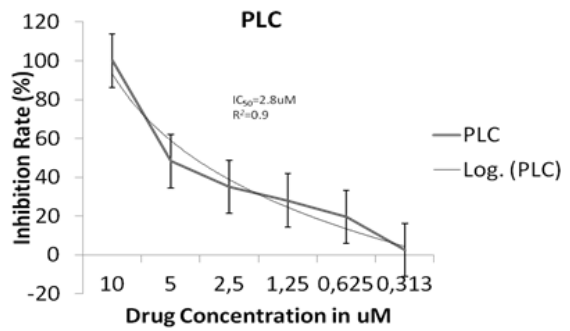

**Figure S1 Inhibition Curves against DAAD-2**

Supplement: Additional file 3: — Figure S1. Inhibition Curves against DAAD-2. Inhibition curves (ranging from 0.313 to 10 μM concentration) of Hep3B, Hep3B-TR, PLC and MCF7 cell lines against DAAD-2. (PDF 144 kb) [file 12885_2016_2942_MOESM3_ESM.pdf]
