# Supplementary material for: Synthesis and bio-molecular study of (+)-N-Acetyl-α-amino acid dehydroabietylamine derivative for the selective therapy of hepatocellular carcinoma
Source: BMC Cancer. 2016 Nov 14;16:883. doi: 10.1186/s12885-016-2942-5 (PMC5109647; doi:10.1186/s12885-016-2942-5)
Supplement: Additional file 6: — Figure S3. Connectivity map of DAAD2 treated SNU449 cells. DAAD-2 treatments caused highly different gene expression alterations in drug-resistant SNU449 cells. (PDF 242 kb) [file 12885_2016_2942_MOESM6_ESM.pdf]

total instances: 6100, *signature*: SNU449_Drug-Control, *export:* Excel

permuted results | isolate shaded

barview	rank	batch	cmap name		dose	cell	score	up	down	ATC	instance_id
	6082	670	merbromin		5 µM	MCF7	-.833	-.107	.282		3439
	6083	767	estradiol		10 nM	MCF7	-.833	-.152	.237	G03CA03	6957
	6084	771	diethylcarbamazine		10 µM	MCF7	-.835	-.082	.308	P02CB02	7425
	6085	630	3-hydroxy-DL-kynurenine		18 µM	HL60	-.837	-.208	.184		1300
	6086	665	betulin		9 µM	HL60	-.838	-.221	.170		2952
	6087	649	atractyloside		5 µM	HL60	-.848	-.146	.251		2573
	6088	771	enalapril		8 µM	MCF7	-.852	-.105	.293	C09AA02	7428
	6089	665	proguanil		14 µM	HL60	-.854	-.176	.223	P01BB01	2944
	6090	747	streptozocin		15 µM	MCF7	-.857	-.104	.296	L01AD04	7193
	6091	627	homatropine		11 µM	MCF7	-.866	-.104	.301	S01FA05	1684
	6092	665	levopropoxyphene		7 µM	HL60	-.870	-.182	.225		2980
	6093	513	rosiglitazone		10 µM	MCF7	-.880	-.110	.301	A10BG02	1071
	6094	631	chlorogenic acid		11 µM	HL60	-.886	-.158	.257		1346
	6095	631	dl-alpha tocopherol		9 µM	HL60	-.887	-.167	.247		1320
	6096	665	lansoprazole		11 µM	HL60	-.908	-.241	.184	A02BC03	2967
	6097	771	dimenhydrinate		9 µM	MCF7	-.913	-.115	.312		7431
	6098	648	streptozocin		15 µM	HL60	-.931	-.204	.231	L01AD04	2535
	6099	665	cyclic adenosine monophosphate		12 µM	HL60	-.978	-.207	.250		2969
	6100	764	vinburnine		14 µM	PC3	-1	-.120	.348	C04AX17	7154

Figure S3: Connectivity map of DAAD2 treated SNU449 cells
