# Supplementary material for: Synthesis and bio-molecular study of (+)-N-Acetyl-α-amino acid dehydroabietylamine derivative for the selective therapy of hepatocellular carcinoma
Source: BMC Cancer. 2016 Nov 14;16:883. doi: 10.1186/s12885-016-2942-5 (PMC5109647; doi:10.1186/s12885-016-2942-5)
Supplement: Additional file 7: — Figure S2. Connectivity map of DAAD2 treated Hep3B cells. DAAD-2 treatments caused gene expression changes in drug-sensitive Hep3B cells similar to other drug molecules. (PDF 163 kb) [file 12885_2016_2942_MOESM7_ESM.pdf]

total instances: 6100, *signature*: HEP3B_Drug-Control_List, *export:* Excel

permuted results | isolate shaded

barview	rank	batch ▲▼	cmap name ▲▼		dose	cell	score ▲▼	up ▲▼	down ▲▼	ATC	instance_id
	1	771	diethylcarbamazine		10 µM	MCF7	1	.161	-.606	P02CB02	7425
	2	720	spiradoline		1 µM	MCF7	.940	.174	-.547		4375
	3	688	brompheniramine		9 µM	PC3	.938	.235	-.485	R06AB01	4013
	4	764	vinburnine		14 µM	PC3	.925	.266	-.444	C04AX17	7154
	5	680	levomepromazine		9 µM	PC3	.925	.179	-.530	N05AA02	3701
	6	632	cimetidine		16 µM	MCF7	.913	.155	-.546	A02BA01	1464
	7	761	adiphenine		11 µM	PC3	.904	.193	-.500		7279
	8	731	racecadotril		10 µM	PC3	.899	.256	-.433	A07XA04	5755
	9	681	myosmine		27 µM	PC3	.894	.227	-.459		3737
	10	735	vigabatrin		31 µM	MCF7	.893	.217	-.468	N03AG04	5415
	11	661	isometheptene		8 µM	HL60	.892	.119	-.565	A03AX10	3145
	12	734	gentamicin		3 µM	PC3	.887	.175	-.505	J D S S S	5883
	13	616	midodrine		14 µM	PC3	.879	.201	-.473	C01CA17	2087
	14	628	diphenhydramine		14 µM	PC3	.876	.273	-.399	D04AA32 R06AA02	1830
	15	628	thiamphenicol		11 µM	PC3	.875	.248	-.423	J01BA02	1826
	16	747	isoniazid		29 µM	MCF7	.872	.163	-.506	J04AC01	7197
	17	661	flucloxacillin		8 µM	HL60	.871	.123	-.545	J01CF05	3128
	18	683	PHA-00745360		1 µM	PC3	.871	.199	-.468		3827
	19	747	doxycycline		8 µM	MCF7	.868	.143	-.523	J01AA02 A01AB22	7195
	20	628	isoflupredone		10 µM	PC3	.865	.224	-.440		1832

Figure S2: Connectivity map of DAAD2 treated Hep3B cells
